# The functional connectome in obsessive-compulsive disorder: resting-state mega-analysis and machine learning classification for the ENIGMA-OCD consortium

**DOI:** 10.1038/s41380-023-02077-0

**Published:** 2023-05-02

**Authors:** Willem B. Bruin, Yoshinari Abe, Pino Alonso, Alan Anticevic, Lea L. Backhausen, Srinivas Balachander, Nuria Bargallo, Marcelo C. Batistuzzo, Francesco Benedetti, Sara Bertolin Triquell, Silvia Brem, Federico Calesella, Beatriz Couto, Damiaan A. J. P. Denys, Marco A. N. Echevarria, Goi Khia Eng, Sónia Ferreira, Jamie D. Feusner, Rachael G. Grazioplene, Patricia Gruner, Joyce Y. Guo, Kristen Hagen, Bjarne Hansen, Yoshiyuki Hirano, Marcelo Q. Hoexter, Neda Jahanshad, Fern Jaspers-Fayer, Selina Kasprzak, Minah Kim, Kathrin Koch, Yoo Bin Kwak, Jun Soo Kwon, Luisa Lazaro, Chiang-Shan R. Li, Christine Lochner, Rachel Marsh, Ignacio Martínez-Zalacaín, Jose M. Menchon, Pedro S. Moreira, Pedro Morgado, Akiko Nakagawa, Tomohiro Nakao, Janardhanan C. Narayanaswamy, Erika L. Nurmi, Jose C. Pariente Zorrilla, John Piacentini, Maria Picó-Pérez, Fabrizio Piras, Federica Piras, Christopher Pittenger, Janardhan Y. C. Reddy, Daniela Rodriguez-Manrique, Yuki Sakai, Eiji Shimizu, Venkataram Shivakumar, Blair H. Simpson, Carles Soriano-Mas, Nuno Sousa, Gianfranco Spalletta, Emily R. Stern, S. Evelyn Stewart, Philip R. Szeszko, Jinsong Tang, Sophia I. Thomopoulos, Anders L. Thorsen, Tokiko Yoshida, Hirofumi Tomiyama, Benedetta Vai, Ilya M. Veer, Ganesan Venkatasubramanian, Nora C. Vetter, Chris Vriend, Susanne Walitza, Lea Waller, Zhen Wang, Anri Watanabe, Nicole Wolff, Je-Yeon Yun, Qing Zhao, Wieke A. van Leeuwen, Hein J. F. van Marle, Laurens A. van de Mortel, Anouk van der Straten, Ysbrand D. van der Werf, ENIGMA-OCD Working Group, Honami Arai, Irene Bollettini, Rosa Calvo Escalona, Ana Coelho, Federica Colombo, Leila Darwich, Martine Fontaine, Toshikazu Ikuta, Jonathan C. Ipser, Asier Juaneda-Seguí, Hitomi Kitagawa, Gerd Kvale, Mafalda Machado-Sousa, Astrid Morer, Takashi Nakamae, Jin Narumoto, Joseph O’Neill, Sho Okawa, Eva Real, Veit Roessner, Joao R. Sato, Cinto Segalàs, Roseli G. Shavitt, Dick J. Veltman, Kei Yamada, Wieke A. van Leeuwen, Hein J. F. van Marle, Laurens A. van de Mortel, Anouk van der Straten, Ysbrand D. van der Werf, Odile A. van den Heuvel, Guido A. van Wingen, Paul M. Thompson, Dan J. Stein, Odile A. van den Heuvel, Guido A. van Wingen

**Affiliations:** 1 Amsterdam UMC location University of Amsterdam, Department of Psychiatry, Meibergdreef 9, Amsterdam, The Netherlands; 2 Amsterdam Neuroscience, Amsterdam, The Netherlands; 3 Department of Psychiatry, Graduate School of Medical Science, Kyoto Prefectural University of Medicine, Kyoto, Japan; 4 Department of Psychiatry, Bellvitge University Hospital, Barcelona, Spain; 5 Department of Clinical Science, Faculty of Medicine, University of Barcelona, Barcelona, Spain; 6 IDIBELL, Bellvitge University Hospital, Barcelona, Spain; 7 CIBERSAM, Instituto de Salud Carlos III, Madrid, Spain; 8 Department of Psychiatry, Yale University, New Haven, CT USA; 9 Department of Child and Adolescent Psychiatry, Faculty of Medicine, Technische Universität Dresden, Dresden, Germany; 10 Department of Psychiatry, National Institute of Mental Health And Neurosciences (NIMHANS), Bangalore, India; 11 Radiology Service, Diagnosis Image Center, Hospital Clinic de Barcelona, Barcelona, Spain; 12 Magnetic Resonance Image Core Facility, Institut d’Investigacions Biomèdiques August Pi i Sunyer (IDIBAPS), Barcelona, Spain; 13 Department of Psychiatry, University of Sao Paulo School of Medicine, Sao Paulo, Brazil; 14 Department of Methods and Techniques in Psychology, Pontifical Catholic University, Sao Paulo, Brazil; 15 Vita-Salute San Raffaele University, Milano, Italy; 16 Psychiatry & Clinical Psychobiology, Division of Neuroscience, IRCCS Scientific Institute Ospedale San Raffaele, Milano, Italy; 17 Bellvitge Biomedical Research Insitute-IDIBELL, Bellvitge University Hospital, Barcelona, Spain; 18 Department of Child and Adolescent Psychiatry and Psychotherapy, University Hospital of Psychiatry, University of Zurich, Zurich, Switzerland; 19 Neuroscience Center Zurich, University of Zurich and ETH Zurich, Zurich, Switzerland; 20 Life and Health Sciences Research Institute (ICVS), School of Medicine, University of Minho, Braga, Portugal; 21 ICVS/3B’s, PT Government Associate Laboratory, Braga/Guimarães, Portugal; 22 Clinical Academic Center—Braga, Braga, Portugal; 23 Department of Psychiatry, New York University Grossman School of Medicine, New York, NY USA; 24 Nathan Kline Institute for Psychiatric Research, Orangeburg, NY USA; 25 Department of Psychiatry, University of Toronto, Toronto, ON Canada; 26 General Adult Psychiatry & Health Systems, Centre for Addiction and Mental Health, Toronto, ON Canada; 27 Women’s and Children’s Health, Karolinska Institutet, Stockholm, Sweden; 28 University of California, San Diego, CA USA; 29 Molde Hospital, Møre og Romsdal Hospital Trust, Molde, Norway; 30 Bergen Center for Brain Plasticity, Haukeland University Hospital, Bergen, Norway; 31 Department of Mental Health, Norwegian University of Science and Technology, Trondheim, Norway; 32 Center for Crisis Psychology, University of Bergen, Bergen, Norway; 33 Research Center for Child Mental Development, Chiba University, Chiba, Japan; 34 Imaging Genetics Center, Stevens Neuroimaging & Informatics Institute, Keck School of Medicine, University of Southern California, Los Angeles, CA USA; 35 Department of Psychiatry, University of British Columbia, Vancouver, BC Canada; 36 Amsterdam UMC, location Vrije Universiteit Amsterdam, Department of Psychiatry, De Boelelaan 1117, Amsterdam, The Netherlands; 37 Amsterdam UMC, location Vrije Universiteit Amsterdam, Department of Anatomy and Neurosciences, De Boelelaan 1117, Amsterdam, The Netherlands; 38 Department of Neuropsychiatry, Seoul National University Hospital, Seoul, Republic of Korea; 39 Department of Psychiatry, Seoul National University College of Medicine, Seoul, Republic of Korea; 40 Department of Neuroradiology, School of Medicine, Klinikum Rechts der Isar, Technical University of Munich, Munich, Germany; 41 Department of Brain and Cognitive Sciences, Seoul National University College of Natural Sciences, Seoul, Republic of Korea; 42 Department of Child and Adolescent Psychiatry and Psychology, Hospital Clinic of Barcelona, Barcelona, Spain; 43 Department of Medicine, University of Barcelona, Barcelona, Spain; 44 SA MRC Unit on Risk and Resilience in Mental Disorders, Department of Psychiatry, Stellenbosch University, Stellenbosch, South Africa; 45 Department of Psychiatry, Columbia University Irving Medical Center, New York, NY USA; 46 Department of Clinical Sciences, University of Barcelona, Barcelona, Spain; 47 Psychological Neuroscience Lab, CIPsi, School of Psychology, University of Minho, Braga, Portugal; 48 Graduate School of Medical Sciences, Kyushu University, Fukuoka-shi, Japan; 49 National Institute of Mental Health And Neurosciences (NIMHANS), Bangalore, India; 50 GVAMHS, Goulburn Valley Health, Shepparton, VIC Australia; 51 Department of Psychiatry and Biobehavioral Sciences, University of California at Los Angeles, Los Angeles, CA USA; 52 Division of Child and Adolescent Psychiatry, UCLA Semel Institute for Neuroscience, Los Angeles, CA USA; 53 Departamento de Psicología Básica, Clínica y Psicobiología, Universitat Jaume I, Castelló de la Plana, Spain; 54 Laboratory of Neuropsychiatry, Department of Clinical and Behavioral Neurology, IRCCS Santa Lucia Foundation, Rome, Italy; 55 Department of Diagnostic and Interventional Neuroradiology, School of Medicine, Technical University of Munich, Munich, Germany; 56 TUM-Neuroimaging Center (TUM-NIC) of Klinikum rechts der Isar, Technische Universität München, Munich, Germany; 57 Graduate School of Systemic Neurosciences (GSN), Ludwig-Maximilians-Universität, Munich, Germany; 58 ATR Brain Information Communication Research Laboratory Group, Kyoto, Japan; 59 United Graduate School of Child Development, Osaka University, Kanazawa University, Hamamatsu University School of Medicine, Chiba University and University of Fukui, Fukui, Japan; 60 Department of Cognitive Behavioral Physiology Graduate School of Medicine, Chiba University, Chiba, Japan; 61 Department of Integrative Medicine, National Institute of Mental Health And Neurosciences (NIMHANS), Bangalore, India; 62 Department of Social Psychology and Quantitative Psychology, Universitat de Barcelona-UB, Barcelona, Spain; 63 Department of Psychiatry and Behavioral Sciences, Baylor College of Medicine, Houston, TX USA; 64 British Columbia Children’s Hospital Research Institute, Vancouver, BC Canada; 65 British Columbia Mental Health and Substance Use Services Research Institute, Vancouver, BC Canada; 66 Department of Psychiatry and Neuroscience, Icahn School of Medicine at Mount Sinai, New York, NY USA; 67 Mental Illness Research, Education and Clinical Center (MIRECC), James J. Peters VA Medical Center, Bronx, NY USA; 68 Department of Psychiatry, Sir Run Run Shaw Hospital, Zhejiang University School of Medicine, Hangzhou, Zhejiang Province China; 69 Department of Developmental Psychology, University of Amsterdam, Amsterdam, The Netherlands; 70 Department of Psychology, Faculty of Natural Sciences, MSB Medical School Berlin, Berlin, Germany; 71 Amsterdam Neuroscience, Compulsivity, Impulsivity & Attention program, Amsterdam, The Netherlands; 72 Amsterdam Neuroscience, Brain Imaging program, Amsterdam, The Netherlands; 73 Department of Psychiatry and Neurosciences CCM, Charité Universitätsmedizin Berlin, corporate member of Freie Universität Berlin and Humboldt-Universität zu Berlin, Berlin, Germany; 74 Shanghai Mental Health Center, Shanghai Jiao Tong University School of Medicine, Shanghai Jiao, China; 75 Yeongeon Student Support Center, Seoul National University College of Medicine, Seoul, Republic of Korea; 76 Amsterdam Neuroscience, Mood Anxiety Psychosis Stress Sleep, Amsterdam, The Netherlands; 77 SA MRC Unit on Risk and Resilience in Mental Disorders, Department of Psychiatry, Neuroscience Institute, University of Cape Town, Cape Town, South Africa; 78 Center for Research on Counseling and Support Services, Tokyo University, Tokyo, Japan; 79 Department of Communication Sciences and Disorders, University of Mississippi, Oxford, MS USA; 80 Department of Psychiatry and Mental Health, Neuroscience Institute, University of Cape Town, Cape Town, South Africa; 81 Department of Clinical Psychology, University of Bergen, Bergen, Norway; 82 UCLA Brain Research Institute, Los Angeles, CA USA; 83 Center of Mathematics, Computing and Cognition, Universidade Federal do ABC, Santo Andre, Brazil; 84 Big Data Sector, Hospital Israelita Albert Einstein, Sao Paulo, Brazil; 85 Department of Radiology, Graduate School of Medical Science, Kyoto Prefectural University of Medicine, Kyoto, Japan

**Keywords:** Neuroscience, Psychiatric disorders, Diagnostic markers

## Abstract

Current knowledge about functional connectivity in obsessive-compulsive disorder (OCD) is based on small-scale studies, limiting the generalizability of results. Moreover, the majority of studies have focused only on predefined regions or functional networks rather than connectivity throughout the entire brain. Here, we investigated differences in resting-state functional connectivity between OCD patients and healthy controls (HC) using mega-analysis of data from 1024 OCD patients and 1028 HC from 28 independent samples of the ENIGMA-OCD consortium. We assessed group differences in whole-brain functional connectivity at both the regional and network level, and investigated whether functional connectivity could serve as biomarker to identify patient status at the individual level using machine learning analysis. The mega-analyses revealed widespread abnormalities in functional connectivity in OCD, with global hypo-connectivity (Cohen’s *d*: -0.27 to -0.13) and few hyper-connections, mainly with the thalamus (Cohen’s *d*: 0.19 to 0.22). Most hypo-connections were located within the sensorimotor network and no fronto-striatal abnormalities were found. Overall, classification performances were poor, with area-under-the-receiver-operating-characteristic curve (AUC) scores ranging between 0.567 and 0.673, with better classification for medicated (AUC = 0.702) than unmedicated (AUC = 0.608) patients versus healthy controls. These findings provide partial support for existing pathophysiological models of OCD and highlight the important role of the sensorimotor network in OCD. However, resting-state connectivity does not so far provide an accurate biomarker for identifying patients at the individual level.

## Introduction

Obsessive-compulsive disorder (OCD) is a debilitating disorder with an estimated lifetime prevalence of 1–3% worldwide [1]. It is characterized by intrusive, irrational and distressing thoughts (obsessions) and repetitive physical or mental acts (compulsions) [2]. Prevailing models of OCD propose that symptomatology is associated with structural and functional brain abnormalities within cortico-striato-thalamo-cortical (CSTC) circuits related to motor, cognitive, affective, and motivational processes [3–8]. These CSTC circuits form parallel, partly segregated feedback loops, projecting from different cortical regions through specific striatal regions to the thalamus with recurrent connections back to the cortex [7–11]. The most recent addition to the CSTC disease model for OCD is the sensorimotor circuit [7, 8]. This circuit includes cortical and subcortical regions involved in the generation and control of motor behaviors and integration of sensory information [7, 8]. The sensorimotor circuit is particularly relevant to OCD given its role in habit formation, sensory motor gating, and inhibitory control processes that could be related to the inability of patients to suppress internally triggered repetitive and intrusive thoughts and behavior [12–16]. It is now recognized that brain regions beyond the CSTC circuitry, including those in frontolimbic, frontoparietal, and cerebellar networks are also involved in OCD [6, 7, 16].

Studies have used resting-state functional connectivity (FC) analyses to investigate the pathophysiology of OCD, which examine the statistical dependencies between the fluctuations in blood oxygenation level–dependent (BOLD) signal of anatomically separated brain regions or networks [17]. Abnormalities in FC have been proposed as candidate markers of psychopathological conditions and as potential predictor of therapeutic outcomes [18, 19]. The majority of OCD resting-state studies have investigated FC in large-scale networks and a priori selected brain regions (seeds) involved in CSTC circuits. A recent meta-analysis evaluated 34 seed-based FC studies in OCD by categorizing seed regions into predefined networks [20]. Results indicated lower connectivity between frontoparietal (FPN; also referred to as “central-executive control”), salience (SN), and default-mode (DMN) networks in line with the proposed “triple network” model of psychopathology [21], as well as altered connectivity (no specific direction of connectivity change) within FPN and striatal regions [20]. Another more recent meta-analysis specifically focused on FC of seed regions that were consistently used across 47 included studies [22]. Here the authors reported altered connectivity between the striatum and cortical networks (caudate hypo-connectivity with FPN regions; caudate hyper-connectivity and nucleus accumbens hypo-connectivity with frontolimbic regions), hypo-connectivity between the striatum and thalamus, and altered connectivity between cingulate and frontolimbic regions (i.e. hyper-connectivity with the ventromedial prefrontal cortex [vmPFC] and hypo-connectivity with the dorsolateral PFC [dlPFC]) [22]. These meta-analyses provide evidence for regions within the CSTC circuitry and large-scale brain networks to play a role in OCD. However, findings are only partially consistent and are primarily based on small, single center studies that do not reflect the wide range of clinical heterogeneity in OCD and may have poor generalizability [23, 24], and have been prone to publication bias [22]. Additionally, the majority of FC studies have tested a limited set of hypotheses and have used seed-based FC of predefined brain regions and appointed networks rather than connectivity throughout the brain.

Against this background, we investigated FC differences across the entire brain using mega-analysis of resting-state functional MRI data of 1024 OCD patients and 1028 healthy controls (HC) from 28 independent samples of the Enhancing Neuro-Imaging and Genetics through Meta-Analysis (ENIGMA) OCD consortium. We assessed group differences in whole-brain FC (i.e., the functional connectome) at both the regional and network level. A whole-brain seed-based approach was chosen to potentially identify altered FC related to OCD in regions and networks that might have been overlooked in previous hypothesis-driven FC studies. Therefore, we did not test specific hypotheses in this study. Recent studies from the ENIGMA-OCD working group have shown distinct alterations in brain structure for different age groups [3, 25]. Pediatric (<18 years) patients showed larger thalamic volume, thinner superior and inferior parietal cortices compared to HC, whereas adult (≥18 years) patients were found to have larger pallidal and smaller hippocampal volumes, lower surface area for the transverse temporal cortex and a thinner inferior parietal cortex [3, 25]. We therefore performed our analyses separately for adult and pediatric participants and also aimed to establish the potential modulating effects of clinical characteristics (i.e., disease severity, age of onset, and medication use) consistent with previous studies from the working group [3, 25, 26]. Additionally, we investigated whether FC could serve as a biomarker to identify patients at the individual level using machine learning analysis [27].

## Methods

### Study population

Data were provided by the ENIGMA-OCD working group and initially comprised 36 independent samples from 24 research institutes around the globe, with neuroimaging and clinical data from adult (≥18 years) and pediatric (<18 years) samples. We considered data from 2895 participants, including 1495 OCD patients (1279 adult, 216 pediatric) and 1400 HC (free of psychopathology and psychotropic medication; 1220 adult, 180 pediatric). Diagnosis was determined in accordance with DSM-IV(-TR) or DSM-5 criteria using structured interviews (see Supplementary Methods for an overview of the questionnaires used). Illness severity was measured using the Yale-Brown Obsessive Compulsive Scale (Y-BOCS) and the Children’s Y-BOCS [28, 29]. We excluded two HC who were using psychotropic medication, 264 participants whose data failed neuroimaging quality control, 111 participants due to excessive motion, 315 participants with insufficient brain coverage and 151 participants from samples with <10 participants per group (see Supplementary Methods and Supplementary Figure 1 for flowchart), resulting in a final sample of 2052 participants from 28 samples including 1024 OCD patients (912 adult, 112 pediatric) and 1028 HC (923 adult, 105 pediatric). An overview of demographic and clinical characteristics can be found in Supplementary Table S1. All participating sites obtained permission from their local institutional review boards or ethics committees to provide coded, de-identified data for analysis, and all study participants or caregivers provided written informed consent.

### Image acquisition and processing

Structural T1-weighted (T1w) and resting-state functional brain MRI data were acquired at 1.5 or 3 tesla and preprocessed locally at each site. rs-fMRI data were obtained for 4–12 min with a repetition time ranging between 700 and 3500 ms (see Supplementary Table S2). The images were analyzed using HALFpipe (Harmonized AnaLysis of Functional MRI pipeline) versions 1.0.0 to 1.2.1 [30], which is based on fMRIPrep [31], following standardized protocols to harmonize analysis and quality control across multiple sites (see http://enigma.ini.usc.edu/protocols/functional-protocols/). Preprocessing included motion correction, slice timing and susceptibility distortion correction (if slice timing details and field maps were available), and spatial normalization. Denoising was performed after resampling the images to standard space and included spatial smoothing, grand mean scaling, and ICA-AROMA to regress out motion artifacts related to head motion, white matter (WM), and cerebrospinal fluid (CSF) [32, 33], and temporal filtering using either band- or high pass filtering (using a Gaussian-weighted high-pass width of 125 s for FC, and a frequency-based band pass filter with a low cut-off of 0.01 Hz and a high cut-off of 0.1 Hz for measures of local brain activity). Additionally, physiological nuisance regressors were extracted for anatomical component correction (aCompCor) using the top five principal components of CSF signal [34]. Images were smoothed with a 6 mm full-width at half-maximum kernel. More details on preprocessing are provided in Supplementary Methods.

### Feature extraction

**Fig. 1:**
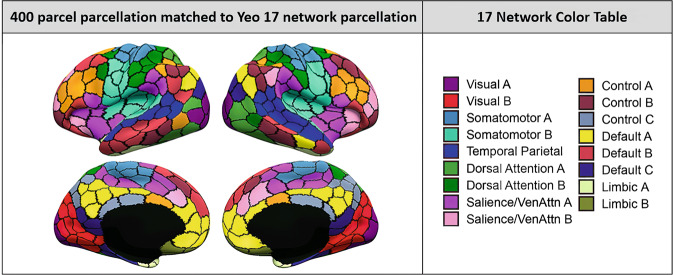
Functional parcellation atlas used to extract time series. Parcels depict the 400 regions-of-interest (ROI) matched to 17 large scale resting-state networks from Schaefer and colleagues [35].

Time series from 434 regions-of-interest (ROIs) were extracted using a combination of functional and structural atlases: 400 ROIs matched to 17 large-scale resting-state networks from the Schaefer atlas (Fig. 1; [35]), 17 subcortical ROIs from the Harvard-Oxford Atlas [36] and 17 cerebellar ROIs from the Buckner 17-network atlas [37]. Time series from ROIs of participants with less than 80% voxel coverage were excluded, reducing the number of ROIs available for analysis (see Supplementary Methods). This procedure resulted in the exclusion of the amygdala and accumbens (ventral striatum) that are of particular interest for OCD. To incorporate these ROIs in the analysis, time series for these ROIs were extracted using 6-mm spheres around peak coordinates in NeuroSynth (neurosynth.org; [38]). A total of 2052 participants with time series from 318 ROIs with sufficient EPI coverage remained, which were used to extract different rs-fMRI features, including pairwise ROI-to-ROI functional connectivity (FC) and measures of local brain activity for each ROI: regional homogeneity (ReHo) measuring the temporal similarity of voxels with their neighbors [39], and fractional amplitude of low frequency fluctuations (fALFF), reflecting the intensity of spontaneous local brain activity [40]. In addition to these ROI-level features, we also calculated network-level FC (between-networks FC after averaging time series of ROIs in each network, and within-network FC for networks that included more than one ROI), and network-level ReHo and fALLF by taking the mean across ROIs from each network (see Supplementary Methods and Supplementary Table S3 for the included regions and network labels).

### Mega-analyses

Linear Mixed Effect (LME) models were used to assess between-group differences, accounting for data clustering within samples with sample-varying effects, with diagnosis (fixed factor, OCD versus HC) as the variable of interest, age, sex, and head motion as covariates, and sample ID as a random intercept. Effect sizes (Cohen’s *d*) and significance (*p* values) were calculated for each ROI and network-level feature [41] (see Supplementary Methods). In our main analysis, we compared all OCD patients versus HC and compared adult and pediatric samples separately. Additionally, we performed stratified group analyses by comparing HC versus patients with and without current use of psychotropic medication at time of scanning, patients with lower severity (Y-BOCS < = 25; mild-moderate [42]) or higher severity of symptoms (Y-BOCS > 25; moderate-severe) based on the median, and adult patients with early (<18 years) and late (≥18 years) age of onset (AO), in line with prior ENIGMA-OCD mega-analyses [3, 25]. Samples with <10 participants per group were excluded for each analysis. Multiple comparisons correction (MCP) was applied for each modality separately (i.e., FC, ReHo and fALFF) using the two-stage Benjamini-Hochberg false discovery rate (FDR) procedure. qFDR for each modality was Bonferroni corrected (0.05/24 (12 contrasts x (ROI + network-wise features)) = 0.0020833) to account for the number of features and contrasts tested simultaneously.

### Machine learning classification

Multivariate classifications were performed using linear Support Vector Machine (SVM) models implemented in scikit-learn (v1.0.2, in Python v3.9.5). Performance was evaluated using 20 times repeated stratified fivefold cross-validation (CV) and measured as the average area-under-the-receiver-operating-characteristic-curve (AUC). Stratified K-fold splits were made by preserving the proportion of patients and controls from each sample. Performance metrics were calculated for each CV iteration on the testing set and averaged across CV iterations. Hyper-parameters were optimized via nested grid-search across different values of C (0.001, 0.01, 0.1, 1, 10) using stratified fivefold CV on training data. Classifications were performed separately for all measures and group comparisons, consistent with previous mega-analyses. Statistical significance of classification performance was assessed using permutation testing with 1000 iterations [43], with Bonferroni correction for the number of classification contrasts for each measure separately (*p* < 0.05/24). Finally, we explored the influence of ComBat harmonization for removing site-effects in our best performing classifier (see Supplementary) [44].

## Results

There was a significant difference in age (mean(SD) = 29.55(10.70) years for OCD; 27.98(9.97) for HC; *t* = 3.42, *p* < 0.001) and biological sex (%male=46.8 for OCD; 52.6 for HC; *X*^*2*^ = 7.02, *p* = 0.008), but corresponding effect sizes were small (*d* = 0.151 for age, *phi* = 0.058 for sex). Mean framewise displacement (FD) was significantly higher (*t* = 3.73, *p* < 0.001) in patients (mean(SD) = 0.11(0.05)) compared to HC (mean(SD) = 0.10(0.05)), but the proportion of high motion volumes (FD > 0.5 mm) was comparable *(t* = 1.70, *p* = 0.090). Data for subgroup analyses are reported in Supplementary Table S4. We included age, sex and mean FD as covariates in our LME models, and performed an additional sensitivity analysis with matched groups (described below). 48.5% of OCD patients used medication, 50.6% had a childhood-onset (<18 years), and the mean severity as assessed with the Y-BOCS was 24.92(6.41).

### Main analysis

**Fig. 2:**
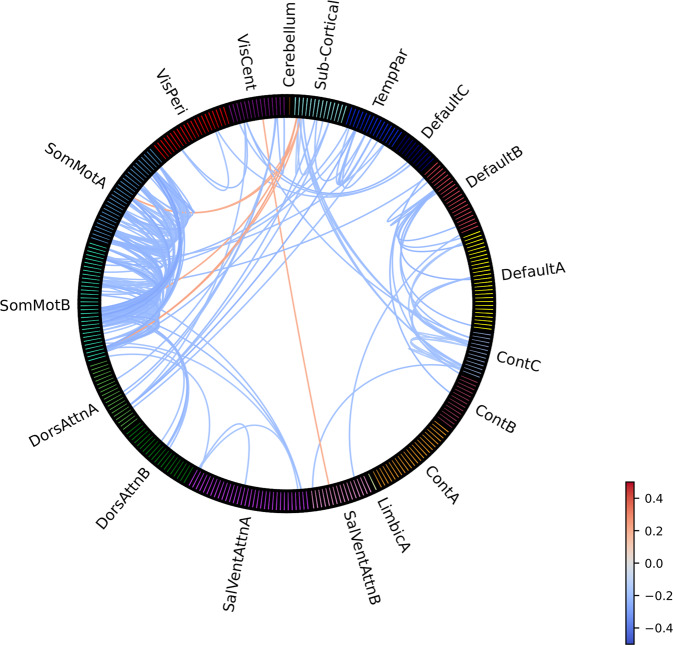
Effect sizes (Cohen’s *d*) for group differences in ROI-to-ROI functional connectivity between OCD patients and HC from pooled samples across age groups. Provided labels in this figure are for assigned networks only (individual ROI labels and ROI to network mapping can be found in Supplement). TempPar Temporal Parietal, Cont Frontoparietal Control, SalVentAttn Salience/Ventral Attention, DorsAttn Dorsal Attention, SomMot Sensorimotor, VisCent Visual Central (Visual A), VisPeri Visual Peripheral (Visual B).

Compared to HC (*N* = 1028), OCD patients (*N* = 1024) showed widespread ROI-to-ROI hypo-connectivity (qFDR<0.05/24), with effect sizes ranging between −0.27 to −0.13 (Fig. 2; for individual ROI labels see Supplementary Fig. 2). ROI-to-ROI FC hypo-connections were predominantly located in sensorimotor (sub)networks, including bilateral primary sensorimotor cortex, supplementary motor area and central sulcus, in default mode (sub)networks (DMN) between bilateral precuneus/posterior cingulate cortex and dorsomedial prefrontal cortex (dmPFC), and between left ventrolateral (vl)PFC (including lateral orbitofrontal cortex) and bilateral dorsal (d)PFC, and in frontoparietal control (sub)networks (FPN; labeled as the “Control” network in the functional atlas) between regions surrounding the posterior cingulate gyrus. ROI-to-ROI hypo-connections between networks were found between bilateral precuneus (in DMN) and posterior cingulate gyrus (in FPN), left insula with right ventral (v)PFC, sensorimotor and salient/ventral attention (SN/VAN) networks, bilateral hippocampus with sensorimotor, and dorsal attention (DAN) and temporoparietal networks. We also found lower FC within bilateral thalamus and right thalamic hypo-connectivity with right caudate nucleus and posterior cingulate gyrus. The only significant hyper-connections were found between bilateral thalamus and right primary sensorimotor cortex and bilateral central sulcus, and between right medial (m)PFC and right extrastriate visual cortex (0.19 < *d* < 0.22). Notably, no significant differences in FC between the frontal cortex and striatum were observed. For network-wise FC, only the sensorimotor networks showed significant within-network hypo-connectivity, with an effect size of −0.18 (Supplementary Fig. 3).

**Fig. 3:**
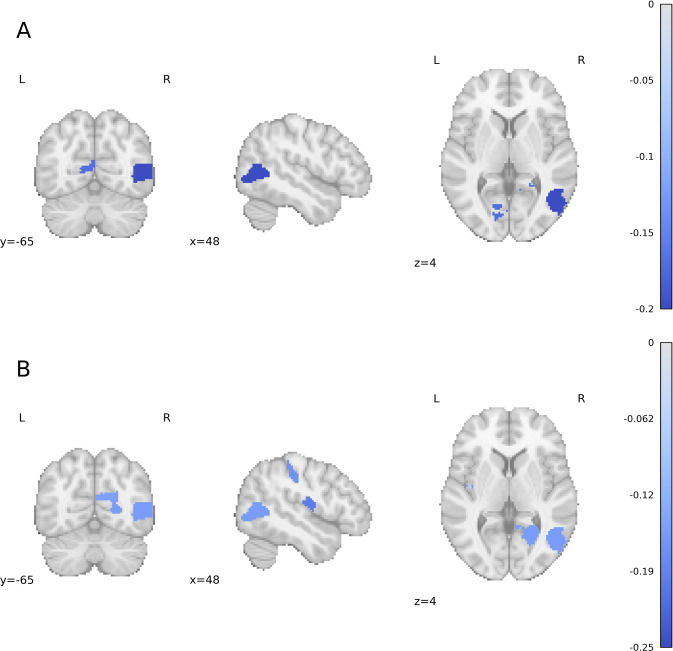
Effect sizes (Cohen’s *d*) for group differences in measures of local activity between OCD patients and HC. (**A**) ReHo, (**B**) regional fALFF. Provided coordinates are in MNI space. L = Left Hemisphere, R = Right Hemisphere.

For measures of local activity, patients showed lower ReHo in the right inferior extrastriate and left calcarine sulcus of the peripheral visual cortex (Visual B), and in the right parietal occipital cortex (−0.2 < *d* < −0.16) (Fig. 3A). For network-wise ReHo, patients showed lower ReHo in the peripheral visual cortex with an effect size of −0.14 (Supplementary Figure 4). Patients showed lower fALFF in the right calcarine sulcus and superior extrastriate of the peripheral visual cortex, bilateral sensorimotor cortex, right parieto-occipital cortex and bilateral postcentral gyri (−0.21 < *d* < −0.15) (Fig. 3B). For network-wise fALFF, patients showed lower fALFF in sensorimotor and DAN networks with effect sizes of −0.21 and −0.16 (Supplementary Figure 5).

### Age

The pediatric sample consisted of 103 patients and 101 HC, and the adult sample of 903 patients and 913 HC. No significant differences were found between pediatric patients and HC for any of the measures. Adult patients showed widespread ROI-to-ROI hypo-connectivity, which broadly resembled that seen in the main analysis (−0.28 < *d* < −0.15), and additionally showed lower FC between left caudate and left posterior cingulate gyrus. Adult patients also showed hyper-connectivity between right thalamus and right central sulcus (consistent with the main analysis; 0.21 < *d* < 0.22). Additional hyperconnectivity was found between right lPFC and left post central gyrus, but no hyperconnectivity between medial PFC and extrastriate visual cortex was observed. The network-wise FC analysis showed additional hypo-connections in adult patients within and between sensorimotor networks, and lower connectivity between temporoparietal and visual central (Visual A) and between subcortical regions and cerebellar networks (−0.19 < *d* < −0.17). Comparisons in local connectivity between adult patients and HC were nearly identical to the findings of the main analysis, with lower ReHo (−0.21 < *d* < −0.18) in most of the same regions (though not in the right inferior extrastriate cortex). No significant differences were found for network-wise ReHo. Adult patients also showed similar but fewer regions with lower regional fALFF (−0.2 < *d* < −0.17), and identical networks with reduced fALFF (−0.2 < *d* < −0.16) (Supplementary Figures 6–11).

### Medication

**Fig. 4:**
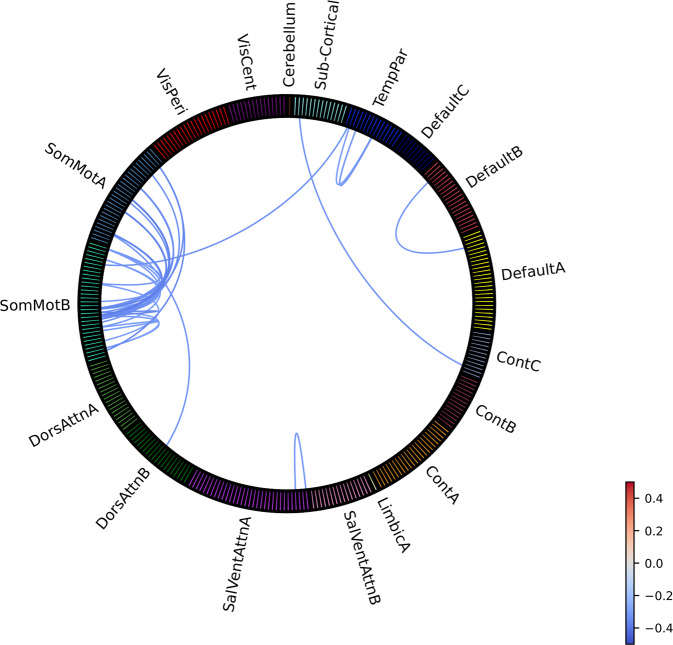
Effect sizes (Cohen’s *d*) for group differences in ROI-to-ROI functional connectivity between medicated OCD patients and HC. Provided labels in this figure are for assigned networks only (individual ROI labels and ROI to network mapping can be found in Supplement). TempPar Temporal Parietal, Cont Frontoparietal Control, SalVentAttn Salience/Ventral Attention, DorsAttn Dorsal Attention, SomMot Sensorimotor, VisCent Visual Central (Visual A), VisPeri Visual Peripheral (Visual B).

The comparison of 342 medicated patients to 509 HC showed ROI-to-ROI hypo-connectivity within and between sensorimotor networks and within and between temporoparietal networks, lower connectivity between left postcentral gyrus and right sensorimotor areas, right medial frontal cortex and right frontal operculum, left vlPFC (including lateral orbitofrontal cortex) and right mPFC and between right thalamus and right posterior cingulate gyrus. The number of significantly different connections was considerably smaller, but the effect sizes were approximately twice as large as for the entire sample, ranging from −0.38 to −0.3 (Fig. 4). Medicated patients also showed lower fALFF in the left central sulcus with an effect size of −0.33 (Supplementary Figure 12). No significant differences were found between medicated patients and HC for network-wise FC or fALFF, or regional and network-wise ReHo. The comparison between 356 unmedicated patients and 420 HC showed no significant group differences for any of the measures. However, the effect sizes for ROI-to-ROI hypo-connections that were found to be significant in the main analysis showed comparable effect sizes in unmedicated patients, ranging between −0.33 to −0.09. No significant differences between medicated and unmedicated patients were observed for any of the measures.

### Symptom severity

**Fig. 5:**
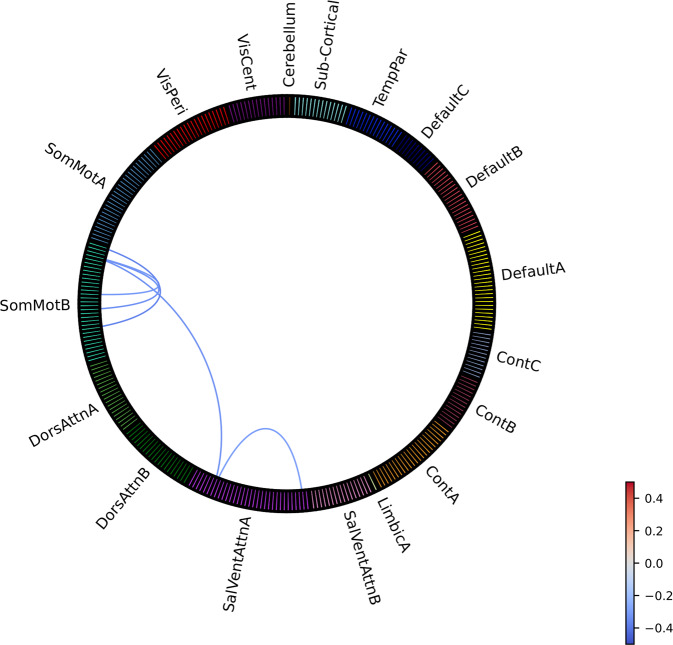
Effect sizes (Cohen’s *d*) for group differences in ROI-to-ROI functional connectivity between high severity OCD patients and HC. Provided labels in this figure are for assigned networks only (individual ROI labels can be found in Supplement). TempPar Temporal Parietal, Cont Frontoparietal Control, SalVentAttn Salience/Ventral Attention, DorsAttn Dorsal Attention, SomMot Sensorimotor, VisCent Visual Central (Visual A), VisPeri Visual Peripheral (Visual B).

The comparison of 376 low-severity patients (YBOCS < = 25) with 598 HC showed no significant group differences. The comparison of 281 high-severity patients with 470 HC showed ROI-to-ROI hypo-connectivity between left and right insula, between left insula and sensorimotor networks and within- and between sensorimotor networks (−0.37 < *d* < −0.3) (Fig. 5, Supplementary Fig. 13), and lower network-wise FC within sensorimotor networks (*d* = −0.34; Supplementary Fig. 14). High-severity patients also showed lower fALFF in sensorimotor networks for both regional and network-averaged features with effect sizes of −0.34 and −0.27, respectively (Supplementary Figs. 15, 16). No significant differences were detected for regional or network-wise ReHo, and no significant differences between low and high severity patients were observed for any of the measures.

### Age of onset

For early-onset (age<18) patient sub-analyses, we included 198 adult patients and 383 HC, and group comparisons showed no significant differences. For late AO patient sub-analyses, we included 300 adult patients and 473 HC. Late AO patients showed lower ROI-to-ROI connectivity between left and right sensorimotor cortical areas (−0.34 < *d* < −0.33) and lower network-wise FC within somatomotor and DMN with effect sizes of −0.25 and −0.34 (Supplementary Figs. 17–18). No significant differences were found for fALFF and ReHo features, and no significant differences between early and late onset patients were observed for any of the measures.

### Sensitivity analysis

To assess whether significant differences in age, sex, and mean FD influenced the results, we repeated our main analysis for ROI-to-ROI FC in an age, sex and mean FD matched sample using propensity score matching (see Supplementary). 811 patients and 797 HC were included (*N* = 1608 subjects from 25 samples). Patients showed widespread ROI-to-ROI hypo-connectivity which largely resembled those seen in main analysis (−0.31 < *d* < −0.15), however the number of significant hypo-connections increased by 64% (*N* = 319 for matched sample comparison, *N* = 194 for main analyses) (Supplementary Figs. 19-20). Notably, 89% of hypo-connections detected in the main analyses remained significant, but three (out of four) hippocampal hypo-connections with sensorimotor, and dorsal attention (DAN) and temporoparietal networks did not. Instead, new significant hypo-connections were found between bilateral hippocampi and regions within sensorimotor networks, as well as cortical—basal ganglia hypo-connections between bilateral caudate and left posterior cingulate, and between left pallidum and right temporal parietal cortex. The hyper-connectivity between regions detected in our main analysis were no longer significant in the matched sample after MCP correction at qFDR = 0.05/24, but these hyper-connections did attain significance at qFDR = 0.05 (0.001<*p*_corrected_ < 0.02) with comparable effect sizes (0.17 < d < 0.21).

### Machine learning

**Fig. 6:**
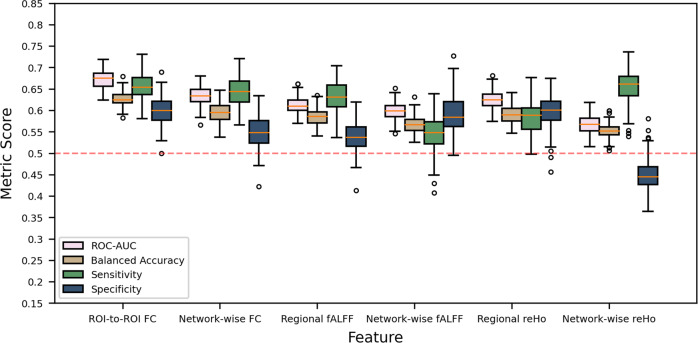
Performance for obsessive-compulsive disorder classification using different resting-state fMRI derived features. Boxplots summarize classification metrics obtained across cross-validation iterations. Dashed lines represent chance-level performance. ROC-AUC area under the received operator curve, ROI region of interest, FC functional connectivity, fALFF fractional amplitude of lower frequency fluctuations, ReHo regional homogeneity.

First, we assessed SVM classification performance to distinguish OCD patients from HC using the complete sample. Overall performance was low though significant, with AUC (averaged across CV folds and repeats) ranging between 0.567 and 0.673 across the different measures (Fig. 6). The best performance was achieved using ROI-to-ROI FC (*p*_corrected_ = 0.024). Classification for adult patients versus HC led to similar performance, with AUCs ranging between 0.565 and 0.684. Performance for pediatric patients versus HC was poor (AUC: 0.542–0.615) and did not reach significance. Classification performance for medicated OCD versus HC using ROI-to-ROI-FC led to an AUC of 0.702 (*p*_corrected_ = 0.024), whereas the classification between unmedicated OCD versus HC was 0.608 (*p*_corrected_ = 0.024). All other classifications (i.e., performed separately for low and high severity, and early and late AO patients versus HC) performed lower than 0.70 AUC. To summarize, twenty seven out of the additional 34 classifications of OCD subgroups versus controls were significant, and seven out of 18 between patient group classifications were significant. A complete overview of classification results, including balanced accuracy, sensitivity, specificity and significance assessed with labels permutations are provided in Supplementary Table S5. We further assessed whether ComBat harmonization improved the performance of our best performing classifier on the complete sample (i.e., using ROI-to-ROI FC). Post-ComBat, the SVM was no longer able to classify the sample of origin above chance level, indicating the successful removal of site-effects. This came at a slight cost in the classification performance for diagnosis and sex (see Supplementary).

## Discussion

Our mega-analyses demonstrated widespread FC aberrations in OCD patients, with global hypo-connectivity and only few hyper-connections. Notably, most of the significant hypo-connections were located within the sensorimotor network. Brain regions involved in the altered connectivity patterns partly correspond to current pathophysiological models of OCD which are mainly based on other neuroimaging modalities [5, 8, 11]. However, our results indicate a lesser degree of subcortical involvement in OCD as measured by resting-state functional MRI, and we did not observe differences in fronto-striatal FC, and only few differences in basal ganglia FC that are central to those models (i.e. between the thalamus and caudate). These results suggest that neural models of OCD should be revised to incorporate hypo-connectivity of the sensorimotor network in particular. Furthermore, despite global hypo-connectivity at the group level, our machine learning results showed that FC could not provide an accurate distinction between OCD patients and HC. Overall classification performance was poor, with AUCs ranging between 0.567–0.673 across the different features when trained on the complete sample, which is insufficient for clinical application [45, 46].

This is the largest FC analysis in OCD conducted to date. Our most consistent finding was lower connectivity within cortical sensorimotor (sub)networks, detected for the majority of OCD versus HC comparisons for both ROI and network-wise FC. The sensorimotor network is often overlooked in OCD studies. There has been some prior evidence OCD related FC alterations in this network [6–8, 14, 16, 47–49]. However, recent meta-analyses on seed-based FC studies in OCD have not reported conclusive findings for the sensorimotor cortex, as studies rarely include seeds within these regions for analysis [20, 22]. The sensorimotor cortex is involved in the generation and control of motor behaviors and integration of sensory information [8]. Alterations in this network could be related to sensory phenomena, aversive or uncomfortable tactile sensations, or perceptions that drive repetitive behaviors [7, 50, 51]. The network is integrated with the sensorimotor CSTC circuit that is relevant to OCD because of its important role in habit formation [6–8]. Altered connectivity within the sensorimotor areas could also reflect impaired sensorimotor gating, the process of suppressing irrelevant sensory, cognitive and motor information to facilitate mental and behavioral integration and flexibility [12]. This could contribute to the inability to inhibit undesired thoughts and images and repetitive behaviors or mental acts [13–15]. The sensorimotor circuit also participates in functions of other neural circuits and vice versa, with the insula and fronto-limbic structures engaged during emotional processing, and early habit formation relying on reward signaling in the ventral affective circuit [7]. Interestingly, a recent transdiagnostic study identified dysconnectivity within the sensorimotor network related to general psychopathology and impulsivity, suggesting that sensorimotor processes affect symptomatology and cognitive function across multiple disorders [52].

In line with previous literature, we identified hypo-connectivity within and between networks of the triple network model i.e., DMN, FPN, SN [20, 21]. The impaired interplay between DMN, FPN, SN could translate into difficulties switching between unwanted, repetitive thoughts and/or compulsions and meaningful, goal-directed action. Secondly, we found aberrant connectivity within CSTC circuits, including thalamic hypo-connectivity with the ventral striatum (including caudate nucleus) and posterior cingulate gyrus regions, and prefrontal hypo-connectivity between CSTC circuits. Our sensitivity analysis using an age, sex and motion matched sample did detect additional cortical—basal ganglia hypo-connections between bilateral caudate and left posterior cingulate, and between left pallidum and right temporal parietal cortex. However, we did not find differences in FC connectivity between prefrontal cortical regions and striatal regions like the putamen, pallidum, caudate nucleus and accumbens, nor did we find FC differences for the amygdala. Of the few FC hyper-connections we observed, most involved connections between (bilateral) thalamus and primary sensorimotor cortex and central sulcus embedded in the sensorimotor CSTC circuit. Recent views on the thalamus hold that it is not a passive relay station but that it has a central role in modulating cortical functioning [53]. This could suggest that thalamic hyperconnectivity with sensorimotor cortical areas may disrupt higher-order corticocortical connectivity within sensorimotor (sub)networks. Interestingly, a recent ENIGMA-OCD study also demonstrated thalamic aberrations, with unmedicated pediatric patients showing larger volumes and adults showing smaller volumes compared to controls [54]. These findings provide further support that the thalamus is a crucial hub in the CSTC circuits and plays a vital role in the pathophysiology underlying OCD. Apart from altered sensorimotor CSTC connectivity, we also found lower connectivity in regions connected to the fronto-limbic CSTC circuit, namely between insula and vPFC, and between cingulate areas and hippocampus, which may be related to altered emotion regulation in OCD [7, 8].

In this study, we also investigated additional rs-fMRI features that describe the intensity of spontaneous brain activity (fALFF) and local synchronization (ReHo). These measures are calculated in a voxel-wise manner and are considered to be reflective of local brain properties, whereas long-range distance correlations (i.e. ROI-to-ROI FC) measure the functional connections between distant brain regions. These measures provide complementary information about the functional organization of the brain and allow for a more detailed and nuanced understanding of brain function. Group differences in measures of local brain activity and synchronization were partly consistent with FC results. OCD patients showed lower fALFF in sensorimotor, visual periphery and dorsal attention network areas, whereas for ReHo patients showed lower activity in dorsal attention network and visual periphery, but not in sensorimotor areas. This is in line with studies reporting lower fALFF and reHo in the sensorimotor cortex and occipital areas [55–58]. However, we did not detect any regions with higher ReHo or fALFF, which have been reported in previous studies [55, 56, 59, 60]. These inconsistencies suggest that the results from these smaller studies might not generalize well to larger samples.

In our secondary analyses we investigated differences between subgroups of participants with specific demographical and clinical characteristics (i.e. age groups, severity of symptoms, age of onset and medication status). No significant case-control differences were detected except for comparisons between adult samples (with changes in FC similar to those seen in our main analyses), and those between late-onset, high-severity and medicated patients versus HC. Few larger scale resting-state studies have investigated the effects of the aforementioned characteristics in OCD. A recent meta-analysis of 47 studies used meta-regression and found a significant negative correlation between the mean age of patients and caudate hypo-connectivity [22]. This is in line with our findings, adult patients showed lower FC between left caudate and posterior cingulate gyrus and this not detected in our main comparisons that included pediatric patients. The meta-regression also showed a negative correlation between age of onset and thalamic-putamen hypo-connectivity, and a negative correlation between symptom severity and hypo-connectivity between the nucleus accumbens and medial orbitofrontal cortex [22]. We did not detect hypo-connectivity between these regions in our age on onset and severity subgroups analyses. Group differences for late-onset adult patients compared to controls were sparse and limited to lower ROI-to-ROI FC in sensorimotor networks, and high severity patients showed only a few hypo-connections, located in sensorimotor networks and within insula regions. This discrepancy in findings could relate to methodological differences between studies. For example, we investigated stratified subgroups rather than regression in line with previous ENIGMA-OCD mega-analyses. Additionally, we used a mega-analytic approach by pooling individual data across studies rather than a meta-analytic approach that synthesizes summary statistics to estimate an overall effect. Our null findings for other case-control comparisons (i.e. pediatric samples, early onset and low severity patients) indicate that these subgroups have no clear association with functional connectivity, but this might also be related to a lack of statistical power as these analyses included fewer patients (*N* = 103, 198 and 376, respectively).

We found the largest effect sizes for differences between medicated patients versus HC, and these were mainly located in sensorimotor regions. We did not detect any significant differences between unmedicated patients and controls. However, exploratory analyses showed that effect sizes for the comparison between unmedicated patients versus controls were comparable with the main analysis. This suggests that findings from the main analysis are not fully driven by medication effects. Furthermore, the lack of significance after multiple comparisons correction might be explained by reduced power due to a smaller sample size compared to our main analysis, and smaller associated effect sizes in unmedicated patients versus controls compared to medicated patients versus controls. These results with larger differences for medicated than unmedicated patients were corroborated by the machine learning analyses, which showed higher classification performance for medicated (0.702 AUC) than unmedicated patients versus control (0.608 AUC). This difference in performance might be explained by medication specific effects that could alter brain structure and function, which would possibly allow for better discrimination between groups. Findings from rodent studies indicate that serotonin reuptake inhibitors (SRIs) promote neuroplasticity in the cortex and subcortex through glio-genesis and neurogenesis [61–63]. However, it is unclear whether these findings translate to humans, and the impact of long-term medication use is not well understood [64]. The influence of medication on FC resembles previous work from ENIGMA-OCD on brain structure; thinner cortices (in adults) and smaller surface areas (in children) were restricted to medicated patients [3, 25]. Similarly, smaller thalamic volume in adults [54] and microstructural alterations in white matter in OCD were mainly driven by medicated patients. Previous ENIGMA-OCD classification analyses based on structural MRI pointed in the same direction, with low overall classification performance between all patients versus controls (AUC = 0.57–0.62) and higher classification performance for medicated patients than for unmedicated patients versus controls (AUC = 0.69 and 0.60, respectively) [26]. But while classification between medicated and unmedicated patients based on structural MRI reached over 0.80 AUC [26], the same classification in this study was 0.56–0.64 AUC. In addition, univariate analyses in the current study showed no significant differences between medication groups. These findings suggest that although the functional differences in OCD are more pronounced in medicated patients, they are not as specific as for brain structure. Little is known about the effects of psychotropic medication on resting-state connectivity in OCD. While meta-analysis of cerebral blood flow and metabolism studies suggests that treatment with SRIs decreases resting caudate nucleus and orbitofrontal cortex activity [65], small-scale FC studies have reported increased striatal connectivity and graph measures of whole-brain connectivity after treatment [66, 67]. However, placebo-controlled studies in healthy volunteers suggest that SRIs primarily reduce FC [68, 69], suggesting that FC normalization after treatment may reflect symptom improvement rather than direct SRI effects. Additional longitudinal studies are warranted to better understand the effects of medication on FC in OCD.

The obtained classification performances in this study are in line with previous work by the ENIGMA-OCD working group using structural MRI. We suspect that the poor overall classification performance is related to the neurobiological heterogeneity underpinning OCD, also related to developmental stage and disease stage [6]. Also, patients can present a variety of symptoms in different combinations, each of which may be caused by distinct brain changes [7]. It is possible that there is no universal biomarker that would be effective for all patients [24]. This heterogeneity is likely to be further exacerbated in a large multicenter study like ENIGMA that combines samples with different scanning parameters, processing pipelines, inclusion criteria and demographic- and other clinical characteristics [70]. In contrast, prior studies using MRI to classify OCD with smaller monocenter samples have reached accuracies up to 93% [18]. These smaller samples are often more homogeneous and use carefully selected patients based on specific inclusion and exclusion criteria to enhance statistical power, but they may not be representative of the broader clinical population. Thus, whereas small monocenter studies focus on answering a specific question about their patient population, large multicenter studies assume that a fundamental pattern of the disorder of interest can be detected despite the presence of heterogeneity, and both are geared toward answering complementary questions [23, 24, 26, 71, 72]. Future classification studies using a similar multicenter setting to our own could investigate the feasibility of using multimodal data (i.e. a combining functional, structural and/or diffusion tensor imaging MRI), deep learning techniques and the use of unsupervised techniques to address phenotypic heterogeneity seen in clinical populations and to stratify disorders into more meaningful subgroups.

Several limitations should be noted. First, we used a retrospectively pooled sample from existing studies across the world, with different scanners and no harmonized data collection protocols. fMRI data were collected with different scanning durations and temporal and spatial resolutions which may affect the signal-to-noise of images and contribute to the heterogeneity of the data. However, we addressed differences between centers through the use of random intercepts in our LME models and Combat harmonization for the machine learning analysis. And whereas variability in fMRI data collection can be viewed as a limitation, it can also be considered a strength as we were able to test if results are robust across diverse protocols and hardware. Second, age, sex, and head motion were significantly different between groups. However, these differences were small in terms of effect size and all performed LME models included these variables as additional covariates in our analyses. In addition, a sensitivity analysis with matched samples showed even more FC differences between groups, suggesting that the results were not driven by group differences in age, sex and head motion. Third, the cross-sectional design of this study did not allow for reliable investigation of medication effects, and limited information was available on history, type, dosage and duration of treatment. Longitudinal studies that incorporate more detailed medication information may provide better insight into the long and short-term effects of medication on FC. Finally, there is a lack of information on OCD subtypes in our dataset. Particular OCD subtypes may be characterized by different neural correlates, and this might limit the ability of machine learning models to find generalizable patterns in brain structure and function [18, 26, 73]. Further studies including detailed clinical information are needed to disentangle this issue.

Taken together, our findings provide evidence for large-scale network aberrations in OCD and highlight the importance of sensorimotor network hypo-connectivity, which should be incorporated into neural models of OCD. Despite abundant hypoconnectivity, univariate effect sizes were small and multivariate classification performance was poor. This indicates that FC does not currently provide an accurate biomarker for OCD, presumably due to disease heterogeneity.

### Supplementary information

Supplementary Information

Alphabetical list of ENIGMA OCD Working Group contributors

## Data Availability

The computer code for the above-described analyses will be provided upon reasonable request.
