# Correction: The functional connectome in obsessive-compulsive disorder: resting-state mega-analysis and machine learning classification for the ENIGMA-OCD consortium

**DOI:** 10.1038/s41380-023-02211-y

**Published:** 2023-08-15

**Authors:** Willem B. Bruin, Yoshinari Abe, Pino Alonso, Alan Anticevic, Lea L. Backhausen, Srinivas Balachander, Nuria Bargallo, Marcelo C. Batistuzzo, Francesco Benedetti, Sara Bertolin Triquell, Silvia Brem, Federico Calesella, Beatriz Couto, Damiaan A. J. P. Denys, Marco A. N. Echevarria, Goi Khia Eng, Sónia Ferreira, Jamie D. Feusner, Rachael G. Grazioplene, Patricia Gruner, Joyce Y. Guo, Kristen Hagen, Bjarne Hansen, Yoshiyuki Hirano, Marcelo Q. Hoexter, Neda Jahanshad, Fern Jaspers-Fayer, Selina Kasprzak, Minah Kim, Kathrin Koch, Yoo Bin Kwak, Jun Soo Kwon, Luisa Lazaro, Chiang-Shan R. Li, Christine Lochner, Rachel Marsh, Ignacio Martínez-Zalacaín, Jose M. Menchon, Pedro S. Moreira, Pedro Morgado, Akiko Nakagawa, Tomohiro Nakao, Janardhanan C. Narayanaswamy, Erika L. Nurmi, Jose C. Pariente Zorrilla, John Piacentini, Maria Picó-Pérez, Fabrizio Piras, Federica Piras, Christopher Pittenger, Janardhan Y. C. Reddy, Daniela Rodriguez-Manrique, Yuki Sakai, Eiji Shimizu, Venkataram Shivakumar, Blair H. Simpson, Carles Soriano-Mas, Nuno Sousa, Gianfranco Spalletta, Emily R. Stern, S. Evelyn Stewart, Philip R. Szeszko, Jinsong Tang, Sophia I. Thomopoulos, Anders L. Thorsen, Tokiko Yoshida, Hirofumi Tomiyama, Benedetta Vai, Ilya M. Veer, Ganesan Venkatasubramanian, Nora C. Vetter, Chris Vriend, Susanne Walitza, Lea Waller, Zhen Wang, Anri Watanabe, Nicole Wolff, Je-Yeon Yun, Qing Zhao, Wieke A. van Leeuwen, Hein J. F. van Marle, Laurens A. van de Mortel, Anouk van der Straten, Ysbrand D. van der Werf, ENIGMA-OCD Working Group, Honami Arai, Irene Bollettini, Rosa Calvo Escalona, Ana Coelho, Federica Colombo, Leila Darwich, Martine Fontaine, Toshikazu Ikuta, Jonathan C. Ipser, Asier Juaneda-Seguí, Hitomi Kitagawa, Gerd Kvale, Mafalda Machado-Sousa, Astrid Morer, Takashi Nakamae, Jin Narumoto, Joseph O’Neill, Sho Okawa, Eva Real, Veit Roessner, Joao R. Sato, Cinto Segalàs, Roseli G. Shavitt, Dick J. Veltman, Kei Yamada, Wieke A. van Leeuwen, Hein J. F. van Marle, Laurens A. van de Mortel, Anouk van der Straten, Ysbrand D. van der Werf, Odile A. van den Heuvel, Guido A. van Wingen, Paul M. Thompson, Dan J. Stein, Odile A. van den Heuvel, Guido A. van Wingen

**Affiliations:** 1 Amsterdam UMC location University of Amsterdam, Department of Psychiatry, Meibergdreef 9, Amsterdam, The Netherlands; 2 Amsterdam Neuroscience, Amsterdam, The Netherlands; 3 Department of Psychiatry, Graduate School of Medical Science, Kyoto Prefectural University of Medicine, Kyoto, Japan; 4 Department of Psychiatry, Bellvitge University Hospital, Barcelona, Spain; 5 Department of Clinical Science, Faculty of Medicine, University of Barcelona, Barcelona, Spain; 6 IDIBELL, Bellvitge University Hospital, Barcelona, Spain; 7 CIBERSAM, Instituto de Salud Carlos III, Madrid, Spain; 8 Department of Psychiatry, Yale University, New Haven, CT USA; 9 Department of Child and Adolescent Psychiatry, Faculty of Medicine, Technische Universität Dresden, Dresden, Germany; 10 Department of Psychiatry, National Institute of Mental Health And Neurosciences (NIMHANS), Bangalore, India; 11 Radiology Service, Diagnosis Image Center, Hospital Clinic de Barcelona, Barcelona, Spain; 12 Magnetic Resonance Image Core Facility, Institut d’Investigacions Biomèdiques August Pi i Sunyer (IDIBAPS), Barcelona, Spain; 13 Department of Psychiatry, University of Sao Paulo School of Medicine, Sao Paulo, Brazil; 14 Department of Methods and Techniques in Psychology, Pontifical Catholic University, Sao Paulo, Brazil; 15 Vita-Salute San Raffaele University, Milano, Italy; 16 Psychiatry & Clinical Psychobiology, Division of Neuroscience, IRCCS Scientific Institute Ospedale San Raffaele, Milano, Italy; 17 Bellvitge Biomedical Research Insitute-IDIBELL, Bellvitge University Hospital, Barcelona, Spain; 18 Department of Child and Adolescent Psychiatry and Psychotherapy, University Hospital of Psychiatry, University of Zurich, Zurich, Switzerland; 19 Neuroscience Center Zurich, University of Zurich and ETH Zurich, Zurich, Switzerland; 20 Life and Health Sciences Research Institute (ICVS), School of Medicine, University of Minho, Braga, Portugal; 21 ICVS/3B’s, PT Government Associate Laboratory, Braga/Guimarães, Portugal; 22 Clinical Academic Center—Braga, Braga, Portugal; 23 Department of Psychiatry, New York University Grossman School of Medicine, New York, NY USA; 24 Nathan Kline Institute for Psychiatric Research, Orangeburg, NY USA; 25 Department of Psychiatry, University of Toronto, Toronto, ON Canada; 26 General Adult Psychiatry & Health Systems, Centre for Addiction and Mental Health, Toronto, ON Canada; 27 Women’s and Children’s Health, Karolinska Institutet, Stockholm, Sweden; 28 University of California, San Diego, CA USA; 29 Molde Hospital, Møre og Romsdal Hospital Trust, Molde, Norway; 30 Bergen Center for Brain Plasticity, Haukeland University Hospital, Bergen, Norway; 31 Department of Mental Health, Norwegian University of Science and Technology, Trondheim, Norway; 32 Center for Crisis Psychology, University of Bergen, Bergen, Norway; 33 Research Center for Child Mental Development, Chiba University, Chiba, Japan; 34 Imaging Genetics Center, Stevens Neuroimaging & Informatics Institute, Keck School of Medicine, University of Southern California, Los Angeles, CA USA; 35 Department of Psychiatry, University of British Columbia, Vancouver, BC Canada; 36 Amsterdam UMC, location Vrije Universiteit Amsterdam, Department of Psychiatry, De Boelelaan 1117, Amsterdam, The Netherlands; 37 Amsterdam UMC, location Vrije Universiteit Amsterdam, Department of Anatomy and Neurosciences, De Boelelaan 1117, Amsterdam, The Netherlands; 38 Department of Neuropsychiatry, Seoul National University Hospital, Seoul, Republic of Korea; 39 Department of Psychiatry, Seoul National University College of Medicine, Seoul, Republic of Korea; 40 Department of Neuroradiology, School of Medicine, Klinikum Rechts der Isar, Technical University of Munich, Munich, Germany; 41 Department of Brain and Cognitive Sciences, Seoul National University College of Natural Sciences, Seoul, Republic of Korea; 42 Department of Child and Adolescent Psychiatry and Psychology, Hospital Clinic of Barcelona, Barcelona, Spain; 43 Department of Medicine, University of Barcelona, Barcelona, Spain; 44 SA MRC Unit on Risk and Resilience in Mental Disorders, Department of Psychiatry, Stellenbosch University, Stellenbosch, South Africa; 45 Department of Psychiatry, Columbia University Irving Medical Center, New York, NY USA; 46 Department of Clinical Sciences, University of Barcelona, Barcelona, Spain; 47 Psychological Neuroscience Lab, CIPsi, School of Psychology, University of Minho, Braga, Portugal; 48 Graduate School of Medical Sciences, Kyushu University, Fukuoka-shi, Japan; 49 National Institute of Mental Health And Neurosciences (NIMHANS), Bangalore, India; 50 GVAMHS, Goulburn Valley Health, Shepparton, VIC Australia; 51 Department of Psychiatry and Biobehavioral Sciences, University of California at Los Angeles, Los Angeles, CA USA; 52 Division of Child and Adolescent Psychiatry,, UCLA Semel Institute for Neuroscience, Los Angeles, CA USA; 53 Departamento de Psicología Básica, Clínica y Psicobiología, Universitat Jaume I, Castelló de la Plana, Spain; 54 Laboratory of Neuropsychiatry, Department of Clinical and Behavioral Neurology, IRCCS Santa Lucia Foundation, Rome, Italy; 55 Department of Diagnostic and Interventional Neuroradiology, School of Medicine, Technical University of Munich, Munich, Germany; 56 TUM-Neuroimaging Center (TUM-NIC) of Klinikum rechts der Isar, Technische Universität München, Munich, Germany; 57 Graduate School of Systemic Neurosciences (GSN), Ludwig-Maximilians-Universität, Munich, Germany; 58 ATR Brain Information Communication Research Laboratory Group, Kyoto, Japan; 59 United Graduate School of Child Development, Osaka University, Kanazawa University, Hamamatsu University School of Medicine, Chiba University and University of Fukui, Fukui, Japan; 60 Department of Cognitive Behavioral Physiology Graduate School of Medicine, Chiba University, Chiba, Japan; 61 Department of Integrative Medicine, National Institute of Mental Health And Neurosciences (NIMHANS), Bangalore, India; 62 Department of Social Psychology and Quantitative Psychology, Universitat de Barcelona-UB, Barcelona, Spain; 63 Department of Psychiatry and Behavioral Sciences, Baylor College of Medicine, Houston, TX USA; 64 British Columbia Children’s Hospital Research Institute, Vancouver, BC Canada; 65 British Columbia Mental Health and Substance Use Services Research Institute, Vancouver, BC Canada; 66 Department of Psychiatry and Neuroscience, Icahn School of Medicine at Mount Sinai, New York, NY USA; 67 Mental Illness Research, Education and Clinical Center (MIRECC), James J. Peters VA Medical Center, Bronx, NY USA; 68 Department of Psychiatry, Sir Run Run Shaw Hospital, Zhejiang University School of Medicine, Hangzhou, Zhejiang Province China; 69 Department of Developmental Psychology, University of Amsterdam, Amsterdam, The Netherlands; 70 Department of Psychology, Faculty of Natural Sciences, MSB Medical School Berlin, Berlin, Germany; 71 Amsterdam Neuroscience, Compulsivity, Impulsivity & Attention program, Amsterdam, The Netherlands; 72 Amsterdam Neuroscience, Brain Imaging program, Amsterdam, The Netherlands; 73 Department of Psychiatry and Neurosciences CCM, Charité Universitätsmedizin Berlin, corporate member of Freie Universität Berlin and Humboldt-Universität zu Berlin, Berlin, Germany; 74 Shanghai Mental Health Center, Shanghai Jiao Tong University School of Medicine, Shanghai Jiao, China; 75 Yeongeon Student Support Center, Seoul National University College of Medicine, Seoul, Republic of Korea; 76 Amsterdam Neuroscience, Mood Anxiety Psychosis Stress Sleep, Amsterdam, The Netherlands; 77 SA MRC Unit on Risk and Resilience in Mental Disorders, Department of Psychiatry, Neuroscience Institute, University of Cape Town, Cape Town, South Africa; 78 Center for Research on Counseling and Support Services, Tokyo University, Tokyo, Japan; 79 Department of Communication Sciences and Disorders, University of Mississippi, Oxford, MS USA; 80 Department of Psychiatry and Mental Health, Neuroscience Institute, University of Cape Town, Cape Town, South Africa; 81 Department of Clinical Psychology, University of Bergen, Bergen, Norway; 82 UCLA Brain Research Institute, Los Angeles, CA USA; 83 Center of Mathematics, Computing and Cognition, Universidade Federal do ABC, Santo Andre, Brazil; 84 Big Data Sector, Hospital Israelita Albert Einstein, Sao Paulo, Brazil; 85 Department of Radiology, Graduate School of Medical Science, Kyoto Prefectural University of Medicine, Kyoto, Japan

**Keywords:** Neuroscience, Psychiatric disorders, Diagnostic markers

Correction to: *Molecular Psychiatry* 10.1038/s41380-023-02077-0, published online 2 May 2023

In this article Honami Arai, Irene Bollettini, Rosa Calvo Escalona, Ana Coelho, Federica Colombo, Leila Darwich, Martine Fontaine, Toshikazu Ikuta, Jonathan C. Ipser, Asier Juaneda-Seguí, Hitomi Kitagawa, Gerd Kvale, Mafalda Machado-Sousa, Astrid Morer, Takashi Nakamae, Jin Narumoto, Joseph O’Neill, Sho Okawa, Eva Real, Veit Roessner, Joao R. Sato, Cinto Segalàs, Roseli G. Shavitt, Dick J. Veltman, Kei Yamada were missing from the author list indexed under the ENIGMA-OCD Working Group. Additionally, there was an error regarding Tokiko Yoshida’s name, where the first name and last name were written in the wrong order.

The original article has been corrected.

