# Supplementary material for: The functional connectome in obsessive-compulsive disorder: resting-state mega-analysis and machine learning classification for the ENIGMA-OCD consortium
Source: Mol Psychiatry. 2023 May 2;28(10):4307–19. doi: 10.1038/s41380-023-02077-0 (PMC10827654; doi:10.1038/s41380-023-02077-0)
Supplement: Supplementary file 1 — Supplementary Information [file 41380_2023_2077_MOESM1_ESM.docx]

**Supplementary Material**

**Instruments used for Diagnosis**

Diagnosis was determined in accordance with DSM-IV or DSM-5 criteria using structured interviews; Mini-International Neuropsychiatric Interview (MINI) and Structured Clinical Interview for DSM Disorders (SCID) were used for adult samples (1, 2), and Schedule for Affective Disorders and Schizophrenia for School-Age Children – Present and Lifetime Version (K-SADS-PL), MINI for Children and Adolescents (MINI-KIDS), and Anxiety Disorders Interview Schedule (ADIS) for pediatric samples (3-5).

**Image Acquisition and Processing**

Structural T1-weighted (T1w) and resting-state functional MRI (rs-fMRI) data were acquired and preprocessed locally at each site. Images were obtained using different field strength with either 1.5 or 3T. Resting state repetition times ranged from 700 to 3500 milliseconds, scan durations from 4 to 12 minutes and a complete overview of acquisition parameters of each sample can be found in **Supplementary Table S2**. The images were analyzed using HALFpipe (Harmonized AnaLysis of Functional MRI pipeline) versions 1.0.0 to 1.2.1 (6), following standardized protocols to harmonize analysis and quality control processes across multiple sites (see <http://enigma.ini.usc.edu/protocols/functional-protocols/>).

HALFpipe is a user-friendly software that relies on fMRIPrep (7) and facilitates reproducible analysis of fMRI data including preprocessing and feature extraction. Preprocessing included motion correction, slice timing and susceptibility distortion correction (if slice timing information and field maps were available), and spatial normalization to standard space. fMRIPrep calculates the parameters for each spatial transformation (i.e., head motion correction with three rotations and three translations using FSL’s *mcflirt*, EPI-to-T1w boundary-based co-registration (BBR) using FSL’s FLIRT with nine degrees-of-freedom (excessive deviation will result in rejecting the BBR refinement and accepting the original, affine registration found by FLIRT), and non-linear spatial normalization of skull-stripped structural images to the *MNI152NLin2009cAsym* template using ANTs’ *antsRegistration*), then concatenates all spatial transformations with ANT’s *antsApplyTransforms*, and applies EPI-to-standard normalization in a single step using Lanczos interpolation. ICA-AROMA was used to minimize the effect of head motion on the preprocessed data (8). ICA-AROMA is an automated noise classification procedure that identifies participant-specific motion components related to head motion, white matter, and cerebrospinal fluid (CSF) in a data-driven fashion, and effectively preserves temporal degrees of freedom while eliminating distance-dependent artifacts (9, 10). Additionally, a set of physiological nuisance regressors were extracted to allow for component-based noise correction using CompCor (11). Principal components for anatomical CompCor were estimated on the preprocessed data within the intersection of a subcortical and subject-specific CSF masks calculated in T1w space (using the inverse EPI-to-T1w transformation), see [fmriprep documentation](https://fmriprep.org/en/20.2.6/outputs.html) for more details. Benchmark studies have shown that ICA-AROMA and anatomical CompCorr based noise correction are effective denoising methods that lead to less estimated loss in temporal degrees of freedom compared to high-parameter models and framewise censoring, and provide a good trade-off between the cost in temporal degrees of freedom and FC distance-dependency (9, 12). Denoising is performed after resampling the rs-fMRI data to standard space and occurs in several steps: first ICA-AROMA is ran to classify noise components, followed by spatial smoothing and grand mean scaling, non-aggressive removal of noise components (orthogonalized to the signal components) estimated by ICA-AROMA from both the preprocessed rs-fMRI data and nuisance time series, low- and/or high-pass temporal filtering, and nuisance regression of the top five aCompCor components. Grand mean scaling was deployed with a mean of 10,000 and smoothing occurred with a 6 mm full-width at half-maximum (FWHM) kernel.

Out of the 2,893 participants with neuroimaging data that had required covariates, 264 were excluded following locally assessed quality control protocols (i.e. visual checking of skull stripping, T1w spatial normalization, EPI tSNR, confounds, ICA-based artifact removal and EPI normalization; quality control instructions can be found at <http://enigma.ini.usc.edu/protocols/functional-protocols/>) and an additional 111 high-motion participants were excluded based on the following three criteria: if any rotation/translation parameters exceeded 4 mm/degrees, if average framewise displacement (FD) exceeded 0.3 mm, or if participants had less than 100 volumes of motion unaffected data (total volumes with FD<0.25mm) (12). A flow chart depicting sample inclusion can be found in **Supplementary Figure 1.**

**Feature Extraction**

A combination of functional and structural atlases were applied to each participant’s preprocessed functional image to extract regional rsfMRI-derived features across the entire brain. Atlases included a 400 regions-of-interest (ROI) matched to 17 large scale resting-state networks [functional atlas](https://github.com/ThomasYeoLab/CBIG/tree/master/stable_projects/brain_parcellation/Schaefer2018_LocalGlobal/Parcellations/MNI) from Schaefer and colleagues (13), 17 subcortical ROIs from the Harvard-Oxford Subcortical Structural Atlas (14) and 17 cerebellar ROIs from the Buckner 17-network atlas (15), resulting in a total of 434 ROIs considered for analysis. Our motivation to use the Schaefer atlas with 400 ROIs was based on several reasons. First, the parcellations are well validated, and most commonly used functional atlases contain 200–400 parcels (13, 16). Second, this atlas has been demonstrated to generalize well in participants ranging from age 6 to 85, which makes it well suited for studies across the human lifespan (13). Third, the atlas was created in a data-driven manner, using a compromise between local gradient and global similarity approaches (13). This was in line with the purpose of our study to potentially find new ROIs related to OCD that might have been overlooked previously. Finally, it has been shown that the Schaeffer atlas with 400 parcels performs well for rsfMRI classification in a similar multi-site setting (i.e. the ABIDE study) compared to other atlases like the Automated Anatomical Labeling and Harvard-Oxford probabilistic atlas (17).

Timeseries from ROIs of participants that had less than 80% voxel coverage with the individual subject’s functional image were automatically excluded by the pipeline. The outputs of these timeseries were non-values (NA) and could not be used for analysis. We therefore had to exclude participants and ROIs with poor coverage to obtain a subset of data without NA’s that could be used for meaningful group comparisons. This is especially relevant for pair-wise functional connectivity analyses, because ROIs with missing timeseries would propagate to entire missing rows/columns in the resulting functional connectivity matrix. In addition, since we investigated network-wise functional connectivity (details described below), missing ROIs would also lead to an inconsistent number of regional timeseries used to compute between-network averages. Two out of 434 ROIs were excluded that had no coverage across all participants, and 15 participants were excluded that had less than 90% of remaining ROIs with 50% coverage. From the remaining participants and ROIs, 67 ROIs were excluded that had less than 90% of participants with 50% coverage across ROIs, or any participants with no coverage. Another 53 ROIs were excluded that had more than 1% of remaining participants with NA’s, and using the remaining selected ROIs, 242 participants were excluded that had any NA. Four Harvard-Oxford subcortical ROIs that are of particular interest for OCD were excluded using the aforementioned criteria: namely the left- and right amygdala and accumbens (ventral striatum). The online platform NeuroSynth ([neurosynth.org](https://neurosynth.org/); (18)) was used to identify the following peak MNI coordinates: left amygdala (-22, -4, -22) and right amygdala (26, -4, -22) using the search term “amygdala”, and left ventral striatum (-12, 8, -8) and right ventral striatum (10, 10, -6) using the search term “ventral striatum”. The aforementioned coordinates were used to create 6 mm spherical seeds to extract their respective timeseries from the preprocessed data. 58 participants had NAs in these additional ROIs and had to be excluded, resulting in a total of 2,148 participants with 318 ROIs. After excluding participants samples with <10 remaining participants per group, this resulted in a final sample of 2,052 participants (1,024 OCD patients and 1,028 healthy controls; see Supplementary Figure 1 for flowchart depicting inclusion procedure). Networks for ROIs belonging to the Harvard-Oxford Subcortical atlas and the amygdala and accumbens seeds were labeled as Subcortical, and those for the Buckner cerebellar atlas as Cerebellar. The resulting ROIs and networks were used to extract both ROI- and network-level rs-fMRI features, including pairwise functional connectivity (FC) as well as measures of local activity: regional homogeneity (ReHo) and fractional amplitude of low frequency fluctuations (fALFF). See Supplementary Table S3 for a full overview of included regions and their assigned network labels.

For FC, temporal filtering was applied using a Gaussian-weighted high-pass width of 125 seconds. This procedure removes temporal drift but leaves most of the signal intact. Average timeseries were extracted for each of the 318 ROIs and used to calculate individual symmetrical 318x318 FC matrices describing pairwise connectivity between ROIs using Pearson’ correlations. Similarly, we derived pairwise between-network FC using averaged timeseries from the 18 unique networks, as well as 16 within-network FC for 16 out of 18 networks that included more than one ROI. The diagonal and upper triangular of the pairwise correlations matrix were discarded, and remaining correlations were normalized to Z-scores with Fisher r-to-z transformation prior to analysis. The resulting feature dimensions were (318×318-1)/2=50,403 for ROI-based FC and (18×17)/2+16=169 for network-based FC. For local activity measures fALFF and ReHO, temporal filtering was applied using frequency-based band pass filter with a low cut-off of 0.01 Hz and a high cut-off of 0.1 Hz, and spatial smoothing with a 6 mm FWHM kernel was only applied after fALLF and ReHo were extracted from the preprocessed data. ReHo describes local similarity between the time series of a given voxel and its nearest neighboring voxels and was calculated using Kendall’s coefficient of concordance (19). fALFF was calculated by dividing the power in the low frequency range (0.01–0.1 Hz) by the power in the entire frequency range (20). Each subjects’ ReHo and fALFF maps were Z-standardized on the individual level by subtracting images with the mean value for the entire brain and dividing by the whole brain standard deviation. The standardized and smoothed ReHo and fALFF maps were then averaged across both ROIs and networks to derive 318 ROI and 18 network-level features for each participant.

**Mega-analyses**

Linear Mixed Effect (LME) models were used to assess between-group differences on the pooled measures of ROI- and network-level FC, ReHo and fALFF. LME models are extensions of linear regression models and efficiently account for clustering of data within samples with sample-varying effects. We used the *lme4* (v1.1_21) and *lmerTest* (v3.1_3) packages in R, and the *Pymer4* (v0.7.5) python-to-R interface to perform the analyses (21-23). Independent variables diagnosis, age, sex, and head motion (framewise displacement) were used as fixed factors, and sample ID was included as a random intercept (LME model: y ~ 1 + Diagnosis + Age + Sex + Motion + (1 | Sample ID)). Models were fit using the restricted maximum likelihood method and effect size estimates were calculated with the Cohen's *d*-statistic appropriate for mixed effect models, and were computed using the *t*-statistic for the factor of interest (diagnosis) in the fitted LME model (24). In our main analysis we compared all OCD patients versus controls and compared adult and pediatric samples separately. Additionally, we performed stratified group comparisons for patients with- and without current use of psychotropic medication at time of scanning, patients with low (YBOCS<=25; mild-moderate (25)) or high severity of symptoms (YBOCS>25; moderate-severe) based on median split, and patients with early (<18 years) and late-onset (≥18 years) age of onset (AO) in line with prior ENIGMA-OCD mega-analyses (26, 27). Samples with <10 participants per group were excluded. Multiple comparison correction (MCP) was applied for each rs-fMRI modality separately (i.e. FC; ReHO and fALFF) using the two-stage Benjamini-Hochberg false discovery rate (FDR) procedure. qFDR for each rs-fMRI modality was Bonferroni corrected (0.05/24 (12 contrasts x ROI + Network-wise features) = 0.0020833) to account for the number of rsfMRI features and contrasts tested simultaneously.

To summarize, the 12 contrasts used for group comparisons were as follows:

1. OCD patients vs. healthy controls – both age groups combined (adult and pediatric samples)

2. OCD patients vs. healthy controls – adult samples

3. OCD patients vs. healthy controls – pediatric samples

4. Medicated OCD patients vs. healthy controls – both age groups combined

5. Unmedicated OCD patients vs. healthy controls – both age groups combined

6. Medicated OCD patients vs. Unmedicated OCD patients – both age groups combined

7. Early age of onset OCD patients vs. healthy controls – adult samples

8. Late age of onset OCD patients vs. healthy controls – adult samples

9. Early age of onset OCD patients vs. Late age of onset OCD patients – adult samples

10. Low severity OCD patients vs. healthy controls – both age groups combined

11. High severity OCD patients vs. healthy controls – both age groups combined

12. Low severity OCD patients vs. High severity OCD patients – both age groups combined

**Machine learning classification**

Classifications were performed using linear support vector machine models (SVM; LIBSVM (28)) implemented in scikit-learn (v1.0.2, in Python v3.9.5). SVM classification performance was evaluated using repeated stratified K-Fold cross-validation (CV) with five folds and 20 repeats. Stratified-K-Fold splits were made by preserving the proportion of patients and controls from each center in which 80% of data was used for classifier training and 20% for testing, and this procedure was repeated 20 times. rs-fMRI features were mean centered using the training data and model performance was measured on the testing set using the area under the receiver operating characteristic curve (AUC), and reported scores were averaged across all folds and repeats. Balanced accuracy, sensitivity and specificity are reported in **Supplementary Table S5**. SVM class weights for C were set to “balanced” mode to automatically adjust weights inversely proportional to class frequencies in the input data to better deal with class imbalance. Hyper-parameters for the SVM were optimized via nested CV: a grid-search was performed across different values of C (penalty given to errors; 0.001, 0.01, 0.1, 1, 10) using stratified 5-Fold CV on training data. Statistical significance of classification performance was assessed using a label permutation-testing framework with 1000 iterations (29). 95% confidence intervals (CI) for AUC were computed using the modified Wald-method (30). Classifications were performed separately for each rsfMRI feature and all (stratified) case-control and within-patient comparisons, consistent with previously described mega-analyses. Finally, we explored the influence of ComBat harmonization for removing site effects in our best performing classifier (31).

**Sensitivity analysis:**

There was a significant difference in age (mean(SD)=29.55(10.70) for OCD; 27.98(9.97) for HC; *t*=3.42, *p*<0.001), biological sex and average framewise displacement (FD; mean(SD)=0.11(0.05) for OCD; 0.10(0.05) for HC, *t*=3.73, *p*<0.001) between patients and HC from pooled samples (across age groups) included for main analysis. To assess if these differences had influenced the results, we repeated our main analysis for case-control differences in ROI-to-ROI FC in a matched subsample.

To this end, we employed propensity score matching to select patients and control samples for each site separately, matched for age, sex and average FD using the *psmpy* package developed by Kline and Luo (32). A nearest neighbors matching (NMM) algorithm without replacement was used, and matching was performed based on the propensity logit score. We performed a grid search to find the caliper size (from 0 to 1 with increments of 0.1) that would guarantee sufficient matching quality (indicated by p>0.05 age, sex and average FD differences between patients and HC) while retaining the highest possible sample size. Using a caliper size of 0.8, the matching procedure resulted in a sample of 811 OCD patients and 797 HC from 25 sites that had at least 10 remaining participants per group, matched for age (mean(SD)=29.12(10.29) for OCD; 28.22(9.93) for HC, *t*=1.79, *p*=0.07), sex (%male=48.6 for OCD; 51.7 for HC; *X2*(1, N=1608)=1.68, *p*=0.19) and average FD (mean(SD)=0.10(0.05) for OCD; 0.10(0.05) for HC, *t*=0.73, *p*=0.46).

Patients showed widespread ROI-to-ROI hypo-connectivity which largely resembled those seen in main analysis (-0.31<d<-0.15), however the number of significant hypo-connections increased by 64% (N=319 for matched sample comparison, N=194 for main analyses) (**Supplementary Figures 19-20**). Notably, 89% of hypo-connections detected in the main analyses remained significant, but three (out of four) hippocampal hypo-connections with sensorimotor, and dorsal attention (DAN) and temporoparietal networks did not. Instead, new significant hypo-connections were found between bilateral hippocampi and regions within sensorimotor networks, as well as cortical – basal ganglia hypo-connections between bilateral caudate and left posterior cingulate, and between left pallidum and right temporal parietal cortex. The hyper-connectivity between regions detected in our main analysis were no longer significant in the matched sample after MCP correction at qFDR=0.05/24, but these hyper-connections did attain significance at qFDR=0.05 (0.001<*p*_corrected_<0.02) with comparable effect sizes (0.17<d<0.21).

**ComBat harmonization:**

We addressed whether ComBat harmonization could further improve the performance for our best performing classifier on the complete sample (i.e. using ROI-to-ROI FC) by removing site-specific effects. To this end, we trained linear SVM models with- and without ComBat harmonization to classify OCD patients from controls using the same evaluation strategy as for our main analysis (repeated stratified K-Fold cross-validation with five folds and 20 repeats). We used the scikit-learn compatible ComBat implementation for Python (v0.20; see <https://github.com/CoAxLab/pycombat>).Importantly, ComBat was applied in a cross-validated manner, in which site-effects were estimated for each CV-iteration separately using only the training data, and consequently removed from both training and test data while preserving the effect of interest (i.e. the class to predict). We then compared model performances obtained for SVMs trained with- and without ComBat, as well as the performance of a “dummy” classifier that randomly assigns labels to test subjects using the class prior probabilities of the training data. First, we compared the effects of ComBat on sex (i.e. male or female) classification, as this is a well-defined classification problem where we expect a relatively high base performance. ComBat was applied to remove site-effects from the ROI-to-ROI FC while preserving the effects of sex. Model performance for the dummy classifier was at chance-level performance, the SVM trained without ComBat led to an average (across CV-iterations) AUC of 0.83, and an average AUC of 0.80 was obtained for the SVM combined with ComBat (**Supplementary Figure 21**). We then assessed the effects of ComBat on our classification of interest (diagnosis) in a similar manner, and resulting classification performances are provided in **Supplementary Figure 22**. As expected, model performance for the dummy classifier was at chance-level. SVM trained without ComBat led to an average AUC of 0.67, whereas SVM trained with ComBat (to remove site-effects while preserving the effects of diagnosis) led to similar but slightly lower performance of 0.64 AUC. Finally, we assessed the effects of ComBat on sample ID (multi-class) classification in which SVMs were trained to identify which of the 28 samples subjects belong to. A One-Versus-Rest scheme was employed to train one SVM for each of the 28 samples to distinguish the corresponding sample from all other samples. Multi-class classification performances are provided in **Supplementary Figure 23**. Dummy classifier performance for sample ID classification was around chance-level performance (1/28 samples) with an average accuracy of 0.06. For SVM trained without ComBat an accuracy of 0.73 was achieved, showing that the SVM is able to successfully identify what sample a subject belongs to using ROI-to-ROI FC data. Sample ID classification for SVM combined with ComBat led to chance-level performance with an average accuracy of 0.03. These findings indicate that although ComBat harmonization did successfully hamper the ability of the SVM to identify which sample a subject belonged to, it hardly changed the performance of our classification of interest.

**Supplementary Figure 1.** Flowchart of patients included for analysis in this study. EPI=Echo Planar Imaging, HC=Healthy Control.

Subjects with neuroimaging and clinical data

*n*=2916

Subjects with required covariates
*n*=2895

OCD patients

*n*=1024

Healthy controls

*n*=1028

Subjects remain after excluding medicated controls
*n*=2893

Subjects remain after excluding samples with insufficient samples
*n*=2463

2 healthy controls excluded that were using psychotropic medication at time of scanning

21 excluded that did not have age, sex or diagnosis information available

Subjects remain after excluding samples with insufficient samples
*n*=2052

96 subjects excluded from five samples with less than 10 examples per class

15 excluded with <90% ROIs with 50% coverage, 242 excluded with missing values in remaining ROIs and 58 excluded with missing values in seeds of interest

55 subjects excluded from three samples with less than 10 examples per class

Subjects with sufficient EPI coverage
*n*=2148

Subjects passed motion criteria
*n*=2518

111 excluded with:
any rotation/translation parameter exceeding 4 mm/degrees, or average FD exceeding 0.3 mm, or less than 100 volumes of motion unaffected data (volumes with FD<0.25mm)

264 excluded following locally assessed MRI quality control protocols for quality of T1w skull stripping, spatial normalization, EPI signal-to-noise, confound timeseries and ICA noise components removal

Subjects passed MRI quality control
*n*=2629

**Supplementary Table S1**. Demographic and clinical information for samples contributing to the ENIGMA-OCD consortium that were included for analysis.

| **Sample** | **Group** | **Age Group** | **Tesla** | **N** | **Age** | **Male (%)** | **Medication** (N unmedicated/medicated/missing) | **Age of Onset** (N pediatric/adult/missing) | **Severity** (Y-BOCS) |
| --- | --- | --- | --- | --- | --- | --- | --- | --- | --- |
| **Amsterdam_VUmc** | **Case** | Adult | 3 | 30 | 37.0 | 43.3 | 28/0/2 | 19/9/2 | 20.8 |
|  | **Control** |  |  | 28 | 37.3 | 46.4 |  |  |  |
| **Bangalore_NIMHANS** | **Case** | Adult | 3 | 185 | 29.6 | 53.5 | 119/66/0 | 64/121/0 | 25.8 |
|  | **Control** |  |  | 186 | 27.1 | 66.1 |  |  |  |
| **Barcelona_Bellvitge/ANTIGA_1.5T** | **Case** | Adult | 1.5 | 48 | 32.8 | 50.0 | 1/47/0 | 19/29/0 | 26.8 |
|  | **Control** |  |  | 79 | 32.4 | 60.8 |  |  |  |
| **Barcelona_Bellvitge/PROV_1.5T** | **Case** | Adult | 1.5 | 41 | 37.0 | 53.7 | 3/38/0 | 15/26/0 | 21.8 |
|  | **Control** |  |  | 20 | 34.3 | 55.0 |  |  |  |
| **Bergen** | **Case** | Adult | 3 | 29 | 30.0 | 31.0 | 20/9/0 | 11/16/2 | 26.3 |
|  | **Control** |  |  | 25 | 30.6 | 28.0 |  |  |  |
| **Braga_UMinho/Braga_1.5T** | **Case** | Adult | 1.5 | 28 | 30.0 | 35.7 | 1/27/0 | 8/19/1 | 27.1 |
|  | **Control** |  |  | 18 | 32.2 | 44.4 |  |  |  |
| **Braga_UMinho/Braga_1.5T_act** | **Case** | Adult | 1.5 | 44 | 27.9 | 50.0 | 0/44/0 | 22/21/1 | 25.9 |
|  | **Control** |  |  | 53 | 25.9 | 32.1 |  |  |  |
| **Braga_UMinho/Braga_3T** | **Case** | Adult | 3 | 32 | 29.5 | 43.8 | 6/26/0 | 19/13/0 | 26.1 |
|  | **Control** |  |  | 26 | 30.7 | 38.5 |  |  |  |
| **Brazil** | **Case** | Adult | 3 | 59 | 38.3 | 32.2 | 23/34/2 | 44/10/5 | 29.2 |
|  | **Control** |  |  | 34 | 33.3 | 44.1 |  |  |  |
| **Cape_Town_UCT/Skyra** | **Case** | Adult | 3 | 23 | 30.8 | 43.5 | 6/17/0 | 18/5/0 | 24.0 |
|  | **Control** |  |  | 15 | 30.1 | 26.7 |  |  |  |
| **Chiba/CHB** | **Case** | Adult | 3 | 16 | 34.1 | 18.8 | 1/15/0 | 0/16/0 | 26.6 |
|  | **Control** |  |  | 27 | 29.0 | 81.5 |  |  |  |
| **Chiba/CHBSRPB** | **Case** | Adult | 3 | 22 | 31.0 | 36.4 | 1/21/0 | 0/22/0 | 24.0 |
|  | **Control** |  |  | 40 | 30.6 | 40.0 |  |  |  |
| **Kyoto_KPU/Kyoto1.5T** | **Case** | Adult | 1.5 | 15 | 31.7 | 33.3 | 10/5/0 | 3/12/0 | 27.3 |
|  | **Control** |  |  | 10 | 34.1 | 30.0 |  |  |  |
| **Kyoto_KPU/Kyoto3T** | **Case** | Adult | 3 | 34 | 33.0 | 32.4 | 34/0/0 | 9/25/0 | 22.5 |
|  | **Control** |  |  | 37 | 29.4 | 48.6 |  |  |  |
| **Milan_HSR** | **Case** | Adult | 3 | 10 | 35.6 | 60.0 | 1/8/1 | 5/2/3 | 31.5 |
|  | **Control** |  |  | 56 | 28.7 | 44.6 |  |  |  |
| **NYSPI_Columbia/Adults** | **Case** | Adult | 3 | 38 | 29.3 | 55.3 | 37/0/1 | 22/15/1 | 25.1 |
|  | **Control** |  |  | 34 | 29.9 | 50.0 |  |  |  |
| **New_York** | **Case** | Adult | 3 | 50 | 29.5 | 36.0 | 15/35/0 | 0/0/50 | 23.9 |
|  | **Control** |  |  | 12 | 37.6 | 50.0 |  |  |  |
| **Seoul_SNU** | **Case** | Adult | 3 | 45 | 24.4 | 64.4 | 45/0/0 | 28/17/0 | 27.5 |
|  | **Control** |  |  | 51 | 23.8 | 68.6 |  |  |  |
| **Shanghai_SMCH** | **Case** | Adult | 3 | 50 | 28.4 | 60.0 | 49/1/0 | 0/50/0 | 24.8 |
|  | **Control** |  |  | 34 | 25.7 | 52.9 |  |  |  |
| **UCLA/Adult** | **Case** | Adult | 3 | 32 | 31.3 | 50.0 | 22/9/1 | 30/1/1 | 24.7 |
|  | **Control** |  |  | 19 | 29.3 | 52.6 |  |  |  |
| **Yale_Pittinger/HCP_Prisma** | **Case** | Adult | 3 | 35 | 30.9 | 45.7 | 23/11/1 | 0/0/35 | 21.8 |
|  | **Control** |  |  | 32 | 29.5 | 62.5 |  |  |  |
| **Yale_Pittinger/HCP_Trio** | **Case** | Adult | 3 | 21 | 37.6 | 23.8 | 21/0/0 | 1/1/19 | 26.1 |
|  | **Control** |  |  | 22 | 33.6 | 36.4 |  |  |  |
| **Yale_Pittinger/Yale_2014** | **Case** | Adult | 3 | 25 | 37.2 | 56.0 | 13/12/0 | 8/17/0 | 27.4 |
|  | **Control** |  |  | 60 | 32.3 | 56.7 |  |  |  |
| **Barcelona_HCPB** | **Case** | Pediatric | 3 | 36 | 15.5 | 52.8 | 5/31/0 | 36/0/0 | 20.1 |
|  | **Control** |  |  | 27 | 15.6 | 51.9 |  |  |  |
| **Chiba/CHBC** | **Case** | Pediatric | 3 | 23 | 13.8 | 52.2 | 9/13/1 | 23/0/0 | 24.8 |
|  | **Control** |  |  | 30 | 13.9 | 60.0 |  |  |  |
| **NYSPI_Columbia/Pediatric** | **Case** | Pediatric | 3 | 21 | 12.8 | 47.6 | 21/0/0 | 21/0/0 | 23.9 |
|  | **Control** |  |  | 14 | 13.1 | 50.0 |  |  |  |
| **Vancouver_BCCHR** | **Case** | Pediatric | 3 | 22 | 15.3 | 40.9 | 3/19/0 | 22/0/0 | 14.2 |
|  | **Control** |  |  | 24 | 14.2 | 33.3 |  |  |  |
| **Yale_Gruner** | **Case** | Pediatric | 3 | 10 | 13.9 | 50.0 | 6/4/0 | 10/0/0 | 25.6 |
|  | **Control** |  |  | 15 | 14.1 | 40.0 |  |  |  |

**Supplementary Table S2**. Available information on scanning acquisition parameters used to obtain structural and functional resting-state data for included ENIGMA-OCD samples.
TR=Repetition Time; TE=Echo Time.

| **Sample** | **Scanner type** | **Field strength** (in Tesla) | **STRUCTURAL MRI PARAMETERS** | | | | | **FUNCTIONAL MRI PARAMETERS** | | | | | **STUDY INCLUSION DATES** | | **DOI to published paper**  (if available) |
| --- | --- | --- | --- | --- | --- | --- | --- | --- | --- | --- | --- | --- | --- | --- | --- |
|  |  |  | **Voxel-size**  (X, Y, Z in mm) | **TR** (ms) | **TE**  (ms) | **Flip angle**  (degrees °) | **Voxel-size**  (X, Y, Z in mm) | | **TR** (ms) | **TE**  (ms) | **Flip angle**   (degrees °) | **N Volumes** | **Start** (YYYY-MM-DD) | **End** (YYYY-MM-DD) |  |
| Amsterdam_VUmc | GE Signa HDxt | 3.0 | 1, 0.98, 0.98 | N/A | N/A | N/A | 3.3, 3.3, 3.0 | | 1800 | 35 | 80 | 200 | 2008 | 2014 | 10.1080/15622975.2017.1353132 |
| Bangalore_NIMHANS | Siemens Skyra | 3.0 | 1, 1, 1 | 1900 | 2.43 | 9 | 3, 3, 3 | | 2000 | 30 | 78 | 153-303 | 2010 | continued | N/A |
| Barcelona_Bellvitge/ANTIGA_1.5T | GE Signa Excite | 1.5 | 1.17, 1.17, 1.2 | 11.8 | 4.2 | 15 | 3.75, 3.75, 5 | | 2000 | 50 | 90 | 120 | 2006-07-19 2009-02-04 | 2007-12-21 2010-03-31 | 10.1016/j.biopsych.2012.10.006 |
| Barcelona_Bellvitge/PROV_1.5T | GE Signa Excite | 1.5 | 1.17, 1.17, 1.2 | 11.8 | 4.2 | 15 | 3.75, 3.75, 5 | | 2000 | 50 | 90 | 120 | 2011-05-12 | continued | 10.1017/S0033291717002288 |
| Barcelona_HCPB | Siemens Magnetom Trio Tim | 3.0 | 1, 0.94, 0.94 | 2300 | 3.01 | 9 | 3, 3, 4 | | 2000 | 29 | 80 | 240 | 2007-07-01 | 2013-06-30 | - |
| Bergen | GE Discovery MR750 | 3.0 | 1, 1, 1 | 7 | 3 | 12 | 3.44, 3.44, 3.3 | | 1800 | 30 | 80 | 160 | 2015-08-24 | 2017-12-11 | 10.1016/j.bpsc.2020.01.007 |
| Braga_UMinho/Braga_1.5T | Siemens Magnetom Avanto | 1.5 | 1, 1, 1 | 2730 | 3.48 | 7 | 3.5, 3.5, 3.5 | | 2000 | 30 | 90 | 180 | 2012-05-23 | 2017-04-07 | 10.1016/j.pscychresns.2019.06.008 |
| Braga_UMinho/Braga_1.5T_act | Siemens Magnetom Avanto | 1.5 | 1, 1, 1 | 2730 | 3.48 | 7 | 3.5, 3.5, 3.5 | | 2000 | 30 | 90 | 180 | 2012-05-23 | 2017-04-07 | 10.1016/j.pscychresns.2019.06.008 |
| Braga_UMinho/Braga_3T | Siemens Verio | 3.0 | 1, 1, 1 | 2420 | 4.12 | 9 | 2, 2, 2 | | 1000 | 27 | 62 | 720 | 2019-05-08 | 2020-11-04 | N/A |
| Brazil | Philips Medical Systems | 3.0 | 1, 1, 1 | 7 | 3.2 | 8 | 3, 3, 3 | | 2000 | 30 | 80 | 130 | 2014-08-19 | 2017-11-27 | N/A |
| Cape_Town_UCT/Skyra | Siemens Skyra | 3.0 | 1.0, 1.0, 1.0 | 2530 | 1.69 | 7 | 3.75, 3.75, 5.0 | | 1730 | 27 | 70 | 298 | 2015-05-28 | continued | N/A |
| Chiba/CHB | GE Discovery MR750 3T | 3.0 | 1, 1, 1 | 8.136 | 3.172 | 15 | 3.3, 3.3, 3.5 | | 2300 | 30 | 81 | 201 | 2013-02-21 | 2018-05-01 | 10.3389/fpsyt.2017.00143 |
| Chiba/CHBC | GE Discovery MR750 3T | 3.0 | 1, 1, 1 | 8.136 | 3.172 | 15 | 3.3, 3.3, 3.5 | | 2300 | 30 | 81 | 201 | 2013-06-19 | continued | N/A |
| Chiba/CHBSRPB | GE Discovery MR750 3T | 3.0 | 1.02, 1.02, 1.2 | 8.168 | 3.18 | 11 | 3.31, 3.31, 3.2 | | 2500 | 30 | 80 | 244 | 2018-06-19 | continued | N/A |
| Kyoto_KPU/Kyoto1.5T | Philips Gyroscan Intera | 1.5 | 0.98, 0.98, 1.50 | N/A | N/A | N/A | 3, 3, 3 | | 2411 | 40 | 80 | 200 | 2009-06-01 | 2010-06-24 | 10.1016/j.eurpsy.2010.09.005 |
| Kyoto_KPU/Kyoto3T | Philips Achieva TX | 3.0 | 1.00, 1.00, 1.00 | 7.1 | 3.3 | 10 | 3, 3, 3 | | 2000 | 30 | 80 | 200 | 2010-08-14 | 2012-03-09 | 10.1016/j.euroneuro.2015.08.017 |
| Milan_HSR | Philips Ingenia CX | 3.0 | 1, 1, 1 | 7.9 | 3.7 | 8 | 3, 3, 3.7 | | 2000 | 30 | 85 | 200 | 2016-10-25 | 2020-11-17 | N/A |
| NYSPI_Columbia/Adults | GE MR 750 | 3.0 | 0.8, 0.8, 0.8 | 7.864 | 3.112 | 12 | 2, 2, 2 | | 850 | 25 | 60 | 544 | 2015-03-03 | 2019-11-18 | 10.1038/s41386-020-00929-9 |
| NYSPI_Columbia/Pediatric | GE MR 750 | 3.0 | 0.8, 0.8, 0.8 | 7.864 | 3.112 | 12 | 2, 2, 2 | | 850 | 25 | 60 | 544 | 2015-04-25 | 2018-05-21 | 10.1038/s41386-020-0613-3 10.1002/da.23187 |
| New_York | 3T Siemens NKI TRIOTIM | 3.0 | 0.80, 0.80, 0.80 | 2400 | 2.01 | 8 | 2.1, 2.1, 2.1 | | 1000 | 25.4 | 60 | 480 | 2018-04-30 | 2020-03-10 | N/A |
| Seoul_SNU | Siemens Trio | 3.0 | 1.0, 1.0, 1.0 | 1670 | 1.89 | 9 | 1.9, 1.9, 3.5 | | 3500 | 30 | 90 | 116 | 2010-05-01 | 2016-08-31 | 10.1002/hbm.23347 10.1016/j.biopsych.2013.09.002 10.30773/pi.2019.0206 |
| Shanghai_SMCH | Siemens VERIO | 3.0 | 1, 1, 1 | 2300 | 2.96 | 9 | 3, 3, 3 | | 3000 | 30 | 85 | 170 | N/A | continued | N/A |
| UCLA/Adult | Siemens Trio | 3.0 | 1, 1, 1 | 1900 | 3.26 | 9 | 3, 3, 3 | | 2000 | 25 | 78 | 208 | 2011-01-07 | 2015-06-30 | 10.1073/pnas.1716686115 10.3389/fpsyt.2015.00074 10.1038/tp.2017.192 10.2147/PRBM.S75106 10.1007/s11682-020-00358-8 10.1038/npp.2017.249 |
| Vancouver_BCCHR | GE Discovery MR750 | 3.0 | 1, 1, 1 | 8.152 | 3.172 | 10 | 3, 3, 4 | | 2000 | 25 | 90 | 150 | 2017 | 2019 | N/A |
| Yale_Gruner | 3T GE Signa | 3.0 | 0.98, 0.98, 1 | N/A | N/A | N/A | 3.75, 3.75, 3 | | 2000 | 30 | 80 | 150 | N/A | N/A | 10.1002/hbm.22551 |
| Yale_Pittinger/HCP_Prisma | 3T Prisma | 3.0 | 0.8, 0.8, 0.8 | N/A | N/A | N/A | 2, 2, 2 | | 800 | 37 | 52 | 500 | N/A | N/A | N/A |
| Yale_Pittinger/HCP_Trio | 3T Siemens Trio | 3.0 | 0.8, 0.8, 0.8 | N/A | N/A | N/A | 2.5, 2.5, 2.5 | | 700 | 31 | 55 | 500 | N/A | N/A | N/A |
| Yale_Pittinger/Yale_2014 | 3T Siemens Trio | 3.0 | 1, 1, 1 | 2530 | 3.66 | 7 | 3.44, 3.44, 4 | | 2000 | 25 | 85 | 300 | N/A | N/A | 10.1016/j.biopsych.2013.10.021 |

**Supplementary Table S3**. Overview of the 318 regions included for analysis and their assigned network labels. These regions were obtained from a combination of atlases and seeds including a 400 regions-of-interest (ROI) matched to 17 large scale resting-state networks [functional atlas](https://github.com/ThomasYeoLab/CBIG/tree/master/stable_projects/brain_parcellation/Schaefer2018_LocalGlobal/Parcellations/MNI) from Schaefer and colleagues (13), 17 subcortical ROIs from the Harvard-Oxford Subcortical Structural Atlas (14), 17 cerebellar ROIs from the Buckner 17-network atlas (15), and four seeds from [neurosynth.org](https://neurosynth.org/); (18). Schaefer IDs correspond to the IDs of the [Schaefer 400 parcel atlas](https://github.com/ThomasYeoLab/CBIG/tree/master/stable_projects/brain_parcellation/Schaefer2018_LocalGlobal/Parcellations/MNI). ROI = Region of Interest.

| **Schaefer ROI ID** | **ROI label** | **Network label** |
| --- | --- | --- |
| 1 | 17Networks_LH_VisCent_ExStr_1 | VisCent |
| 2 | 17Networks_LH_VisCent_ExStr_2 | VisCent |
| 6 | 17Networks_LH_VisCent_ExStr_6 | VisCent |
| 8 | 17Networks_LH_VisCent_ExStr_7 | VisCent |
| 9 | 17Networks_LH_VisCent_ExStr_8 | VisCent |
| 10 | 17Networks_LH_VisCent_ExStr_9 | VisCent |
| 12 | 17Networks_LH_VisCent_ExStr_11 | VisCent |
| 13 | 17Networks_LH_VisPeri_ExStrInf_1 | VisPeri |
| 14 | 17Networks_LH_VisPeri_ExStrInf_2 | VisPeri |
| 15 | 17Networks_LH_VisPeri_ExStrInf_3 | VisPeri |
| 16 | 17Networks_LH_VisPeri_ExStrInf_4 | VisPeri |
| 17 | 17Networks_LH_VisPeri_ExStrInf_5 | VisPeri |
| 18 | 17Networks_LH_VisPeri_StriCal_1 | VisPeri |
| 19 | 17Networks_LH_VisPeri_StriCal_2 | VisPeri |
| 20 | 17Networks_LH_VisPeri_ExStrSup_1 | VisPeri |
| 21 | 17Networks_LH_VisPeri_ExStrSup_2 | VisPeri |
| 22 | 17Networks_LH_VisPeri_ExStrSup_3 | VisPeri |
| 24 | 17Networks_LH_VisPeri_ExStrSup_5 | VisPeri |
| 25 | 17Networks_LH_SomMotA_1 | SomMotA |
| 26 | 17Networks_LH_SomMotA_2 | SomMotA |
| 28 | 17Networks_LH_SomMotA_4 | SomMotA |
| 29 | 17Networks_LH_SomMotA_5 | SomMotA |
| 30 | 17Networks_LH_SomMotA_6 | SomMotA |
| 31 | 17Networks_LH_SomMotA_7 | SomMotA |
| 32 | 17Networks_LH_SomMotA_8 | SomMotA |
| 33 | 17Networks_LH_SomMotA_9 | SomMotA |
| 34 | 17Networks_LH_SomMotA_10 | SomMotA |
| 35 | 17Networks_LH_SomMotA_11 | SomMotA |
| 36 | 17Networks_LH_SomMotA_12 | SomMotA |
| 37 | 17Networks_LH_SomMotA_13 | SomMotA |
| 39 | 17Networks_LH_SomMotA_15 | SomMotA |
| 41 | 17Networks_LH_SomMotA_17 | SomMotA |
| 44 | 17Networks_LH_SomMotB_Aud_1 | SomMotB |
| 45 | 17Networks_LH_SomMotB_Aud_2 | SomMotB |
| 46 | 17Networks_LH_SomMotB_Ins_1 | SomMotB |
| 47 | 17Networks_LH_SomMotB_S2_1 | SomMotB |
| 48 | 17Networks_LH_SomMotB_S2_2 | SomMotB |
| 49 | 17Networks_LH_SomMotB_Aud_3 | SomMotB |
| 50 | 17Networks_LH_SomMotB_Aud_4 | SomMotB |
| 51 | 17Networks_LH_SomMotB_S2_3 | SomMotB |
| 52 | 17Networks_LH_SomMotB_S2_4 | SomMotB |
| 53 | 17Networks_LH_SomMotB_S2_5 | SomMotB |
| 54 | 17Networks_LH_SomMotB_S2_6 | SomMotB |
| 55 | 17Networks_LH_SomMotB_Cent_1 | SomMotB |
| 56 | 17Networks_LH_SomMotB_Cent_2 | SomMotB |
| 57 | 17Networks_LH_SomMotB_Cent_3 | SomMotB |
| 58 | 17Networks_LH_SomMotB_Cent_4 | SomMotB |
| 59 | 17Networks_LH_SomMotB_Cent_5 | SomMotB |
| 61 | 17Networks_LH_DorsAttnA_TempOcc_2 | DorsAttnA |
| 63 | 17Networks_LH_DorsAttnA_TempOcc_4 | DorsAttnA |
| 64 | 17Networks_LH_DorsAttnA_ParOcc_1 | DorsAttnA |
| 65 | 17Networks_LH_DorsAttnA_ParOcc_2 | DorsAttnA |
| 66 | 17Networks_LH_DorsAttnA_SPL_1 | DorsAttnA |
| 67 | 17Networks_LH_DorsAttnA_SPL_2 | DorsAttnA |
| 68 | 17Networks_LH_DorsAttnA_SPL_3 | DorsAttnA |
| 69 | 17Networks_LH_DorsAttnA_SPL_4 | DorsAttnA |
| 70 | 17Networks_LH_DorsAttnA_SPL_5 | DorsAttnA |
| 73 | 17Networks_LH_DorsAttnB_PostC_1 | DorsAttnB |
| 74 | 17Networks_LH_DorsAttnB_PostC_2 | DorsAttnB |
| 75 | 17Networks_LH_DorsAttnB_PostC_3 | DorsAttnB |
| 76 | 17Networks_LH_DorsAttnB_PostC_4 | DorsAttnB |
| 77 | 17Networks_LH_DorsAttnB_PostC_5 | DorsAttnB |
| 78 | 17Networks_LH_DorsAttnB_PostC_6 | DorsAttnB |
| 82 | 17Networks_LH_DorsAttnB_FEF_1 | DorsAttnB |
| 83 | 17Networks_LH_DorsAttnB_FEF_2 | DorsAttnB |
| 84 | 17Networks_LH_DorsAttnB_FEF_3 | DorsAttnB |
| 85 | 17Networks_LH_DorsAttnB_PrCv_1 | DorsAttnB |
| 86 | 17Networks_LH_SalVentAttnA_ParOper_1 | SalVentAttnA |
| 87 | 17Networks_LH_SalVentAttnA_ParOper_2 | SalVentAttnA |
| 88 | 17Networks_LH_SalVentAttnA_ParOper_3 | SalVentAttnA |
| 89 | 17Networks_LH_SalVentAttnA_Ins_1 | SalVentAttnA |
| 90 | 17Networks_LH_SalVentAttnA_Ins_2 | SalVentAttnA |
| 91 | 17Networks_LH_SalVentAttnA_Ins_3 | SalVentAttnA |
| 92 | 17Networks_LH_SalVentAttnA_Ins_4 | SalVentAttnA |
| 93 | 17Networks_LH_SalVentAttnA_FrOper_1 | SalVentAttnA |
| 94 | 17Networks_LH_SalVentAttnA_FrOper_2 | SalVentAttnA |
| 95 | 17Networks_LH_SalVentAttnA_ParMed_1 | SalVentAttnA |
| 96 | 17Networks_LH_SalVentAttnA_ParMed_2 | SalVentAttnA |
| 97 | 17Networks_LH_SalVentAttnA_ParMed_3 | SalVentAttnA |
| 98 | 17Networks_LH_SalVentAttnA_FrMed_1 | SalVentAttnA |
| 99 | 17Networks_LH_SalVentAttnA_FrMed_2 | SalVentAttnA |
| 101 | 17Networks_LH_SalVentAttnB_PFCl_1 | SalVentAttnB |
| 102 | 17Networks_LH_SalVentAttnB_PFCl_2 | SalVentAttnB |
| 103 | 17Networks_LH_SalVentAttnB_PFCl_3 | SalVentAttnB |
| 104 | 17Networks_LH_SalVentAttnB_Ins_1 | SalVentAttnB |
| 105 | 17Networks_LH_SalVentAttnB_Ins_2 | SalVentAttnB |
| 106 | 17Networks_LH_SalVentAttnB_Ins_3 | SalVentAttnB |
| 108 | 17Networks_LH_SalVentAttnB_PFCmp_1 | SalVentAttnB |
| 120 | 17Networks_LH_LimbicA_TempPole_7 | LimbicA |
| 121 | 17Networks_LH_ContA_Temp_1 | ContA |
| 122 | 17Networks_LH_ContA_IPS_1 | ContA |
| 124 | 17Networks_LH_ContA_IPS_3 | ContA |
| 125 | 17Networks_LH_ContA_IPS_4 | ContA |
| 126 | 17Networks_LH_ContA_IPS_5 | ContA |
| 127 | 17Networks_LH_ContA_PFCd_1 | ContA |
| 128 | 17Networks_LH_ContA_PFClv_1 | ContA |
| 129 | 17Networks_LH_ContA_PFClv_2 | ContA |
| 130 | 17Networks_LH_ContA_PFCl_1 | ContA |
| 131 | 17Networks_LH_ContA_PFCl_2 | ContA |
| 132 | 17Networks_LH_ContA_PFCl_3 | ContA |
| 133 | 17Networks_LH_ContA_Cingm_1 | ContA |
| 136 | 17Networks_LH_ContB_IPL_1 | ContB |
| 137 | 17Networks_LH_ContB_IPL_2 | ContB |
| 138 | 17Networks_LH_ContB_IPL_3 | ContB |
| 139 | 17Networks_LH_ContB_PFCd_1 | ContB |
| 142 | 17Networks_LH_ContB_PFClv_3 | ContB |
| 143 | 17Networks_LH_ContB_PFCmp_1 | ContB |
| 144 | 17Networks_LH_ContC_pCun_1 | ContC |
| 145 | 17Networks_LH_ContC_pCun_2 | ContC |
| 146 | 17Networks_LH_ContC_pCun_3 | ContC |
| 147 | 17Networks_LH_ContC_Cingp_1 | ContC |
| 148 | 17Networks_LH_ContC_Cingp_2 | ContC |
| 149 | 17Networks_LH_DefaultA_IPL_1 | DefaultA |
| 151 | 17Networks_LH_DefaultA_PFCd_1 | DefaultA |
| 152 | 17Networks_LH_DefaultA_PFCd_2 | DefaultA |
| 153 | 17Networks_LH_DefaultA_PFCd_3 | DefaultA |
| 154 | 17Networks_LH_DefaultA_pCunPCC_1 | DefaultA |
| 155 | 17Networks_LH_DefaultA_pCunPCC_2 | DefaultA |
| 156 | 17Networks_LH_DefaultA_pCunPCC_3 | DefaultA |
| 157 | 17Networks_LH_DefaultA_pCunPCC_4 | DefaultA |
| 158 | 17Networks_LH_DefaultA_pCunPCC_5 | DefaultA |
| 159 | 17Networks_LH_DefaultA_pCunPCC_6 | DefaultA |
| 160 | 17Networks_LH_DefaultA_pCunPCC_7 | DefaultA |
| 163 | 17Networks_LH_DefaultA_PFCm_3 | DefaultA |
| 164 | 17Networks_LH_DefaultA_PFCm_4 | DefaultA |
| 166 | 17Networks_LH_DefaultA_PFCm_6 | DefaultA |
| 170 | 17Networks_LH_DefaultB_Temp_4 | DefaultB |
| 171 | 17Networks_LH_DefaultB_Temp_5 | DefaultB |
| 172 | 17Networks_LH_DefaultB_Temp_6 | DefaultB |
| 173 | 17Networks_LH_DefaultB_IPL_1 | DefaultB |
| 174 | 17Networks_LH_DefaultB_IPL_2 | DefaultB |
| 175 | 17Networks_LH_DefaultB_PFCd_1 | DefaultB |
| 177 | 17Networks_LH_DefaultB_PFCd_3 | DefaultB |
| 180 | 17Networks_LH_DefaultB_PFCd_6 | DefaultB |
| 181 | 17Networks_LH_DefaultB_PFCl_1 | DefaultB |
| 182 | 17Networks_LH_DefaultB_PFCl_2 | DefaultB |
| 183 | 17Networks_LH_DefaultB_PFCv_1 | DefaultB |
| 186 | 17Networks_LH_DefaultB_PFCv_4 | DefaultB |
| 187 | 17Networks_LH_DefaultB_PFCv_5 | DefaultB |
| 188 | 17Networks_LH_DefaultC_IPL_1 | DefaultC |
| 189 | 17Networks_LH_DefaultC_Rsp_1 | DefaultC |
| 190 | 17Networks_LH_DefaultC_Rsp_2 | DefaultC |
| 191 | 17Networks_LH_DefaultC_Rsp_3 | DefaultC |
| 193 | 17Networks_LH_DefaultC_PHC_2 | DefaultC |
| 194 | 17Networks_LH_DefaultC_PHC_3 | DefaultC |
| 195 | 17Networks_LH_TempPar_1 | TempPar |
| 196 | 17Networks_LH_TempPar_2 | TempPar |
| 197 | 17Networks_LH_TempPar_3 | TempPar |
| 198 | 17Networks_LH_TempPar_4 | TempPar |
| 199 | 17Networks_LH_TempPar_5 | TempPar |
| 200 | 17Networks_LH_TempPar_6 | TempPar |
| 201 | 17Networks_RH_VisCent_ExStr_1 | VisCent |
| 202 | 17Networks_RH_VisCent_ExStr_2 | VisCent |
| 203 | 17Networks_RH_VisCent_ExStr_3 | VisCent |
| 204 | 17Networks_RH_VisCent_ExStr_4 | VisCent |
| 207 | 17Networks_RH_VisCent_Striate_1 | VisCent |
| 208 | 17Networks_RH_VisCent_ExStr_7 | VisCent |
| 210 | 17Networks_RH_VisCent_ExStr_9 | VisCent |
| 212 | 17Networks_RH_VisCent_ExStr_11 | VisCent |
| 213 | 17Networks_RH_VisPeri_ExStrInf_1 | VisPeri |
| 214 | 17Networks_RH_VisPeri_ExStrInf_2 | VisPeri |
| 215 | 17Networks_RH_VisPeri_ExStrInf_3 | VisPeri |
| 216 | 17Networks_RH_VisPeri_ExStrInf_4 | VisPeri |
| 217 | 17Networks_RH_VisPeri_ExStrInf_5 | VisPeri |
| 218 | 17Networks_RH_VisPeri_StriCal_1 | VisPeri |
| 219 | 17Networks_RH_VisPeri_StriCal_2 | VisPeri |
| 220 | 17Networks_RH_VisPeri_ExStrSup_1 | VisPeri |
| 221 | 17Networks_RH_VisPeri_ExStrSup_2 | VisPeri |
| 222 | 17Networks_RH_VisPeri_ExStrSup_3 | VisPeri |
| 224 | 17Networks_RH_SomMotA_1 | SomMotA |
| 225 | 17Networks_RH_SomMotA_2 | SomMotA |
| 226 | 17Networks_RH_SomMotA_3 | SomMotA |
| 227 | 17Networks_RH_SomMotA_4 | SomMotA |
| 228 | 17Networks_RH_SomMotA_5 | SomMotA |
| 229 | 17Networks_RH_SomMotA_6 | SomMotA |
| 230 | 17Networks_RH_SomMotA_7 | SomMotA |
| 231 | 17Networks_RH_SomMotA_8 | SomMotA |
| 232 | 17Networks_RH_SomMotA_9 | SomMotA |
| 233 | 17Networks_RH_SomMotA_10 | SomMotA |
| 234 | 17Networks_RH_SomMotA_11 | SomMotA |
| 235 | 17Networks_RH_SomMotA_12 | SomMotA |
| 237 | 17Networks_RH_SomMotA_14 | SomMotA |
| 238 | 17Networks_RH_SomMotA_15 | SomMotA |
| 244 | 17Networks_RH_SomMotB_Aud_1 | SomMotB |
| 245 | 17Networks_RH_SomMotB_Aud_2 | SomMotB |
| 246 | 17Networks_RH_SomMotB_Ins_1 | SomMotB |
| 247 | 17Networks_RH_SomMotB_S2_1 | SomMotB |
| 248 | 17Networks_RH_SomMotB_S2_2 | SomMotB |
| 249 | 17Networks_RH_SomMotB_Aud_3 | SomMotB |
| 250 | 17Networks_RH_SomMotB_S2_3 | SomMotB |
| 251 | 17Networks_RH_SomMotB_S2_4 | SomMotB |
| 252 | 17Networks_RH_SomMotB_S2_5 | SomMotB |
| 253 | 17Networks_RH_SomMotB_S2_6 | SomMotB |
| 254 | 17Networks_RH_SomMotB_S2_7 | SomMotB |
| 255 | 17Networks_RH_SomMotB_S2_8 | SomMotB |
| 256 | 17Networks_RH_SomMotB_Cent_1 | SomMotB |
| 257 | 17Networks_RH_SomMotB_Cent_2 | SomMotB |
| 258 | 17Networks_RH_SomMotB_Cent_3 | SomMotB |
| 261 | 17Networks_RH_DorsAttnA_TempOcc_3 | DorsAttnA |
| 262 | 17Networks_RH_DorsAttnA_ParOcc_1 | DorsAttnA |
| 263 | 17Networks_RH_DorsAttnA_ParOcc_2 | DorsAttnA |
| 264 | 17Networks_RH_DorsAttnA_ParOcc_3 | DorsAttnA |
| 265 | 17Networks_RH_DorsAttnA_SPL_1 | DorsAttnA |
| 266 | 17Networks_RH_DorsAttnA_SPL_2 | DorsAttnA |
| 268 | 17Networks_RH_DorsAttnA_SPL_4 | DorsAttnA |
| 269 | 17Networks_RH_DorsAttnA_SPL_5 | DorsAttnA |
| 270 | 17Networks_RH_DorsAttnA_SPL_6 | DorsAttnA |
| 271 | 17Networks_RH_DorsAttnA_SPL_7 | DorsAttnA |
| 273 | 17Networks_RH_DorsAttnB_TempOcc_1 | DorsAttnB |
| 274 | 17Networks_RH_DorsAttnB_PostC_1 | DorsAttnB |
| 275 | 17Networks_RH_DorsAttnB_PostC_2 | DorsAttnB |
| 276 | 17Networks_RH_DorsAttnB_PostC_3 | DorsAttnB |
| 277 | 17Networks_RH_DorsAttnB_PostC_4 | DorsAttnB |
| 278 | 17Networks_RH_DorsAttnB_PostC_5 | DorsAttnB |
| 279 | 17Networks_RH_DorsAttnB_PostC_6 | DorsAttnB |
| 282 | 17Networks_RH_DorsAttnB_FEF_1 | DorsAttnB |
| 283 | 17Networks_RH_DorsAttnB_FEF_2 | DorsAttnB |
| 284 | 17Networks_RH_DorsAttnB_FEF_3 | DorsAttnB |
| 285 | 17Networks_RH_SalVentAttnA_ParOper_1 | SalVentAttnA |
| 286 | 17Networks_RH_SalVentAttnA_ParOper_2 | SalVentAttnA |
| 287 | 17Networks_RH_SalVentAttnA_ParOper_3 | SalVentAttnA |
| 288 | 17Networks_RH_SalVentAttnA_PrC_1 | SalVentAttnA |
| 289 | 17Networks_RH_SalVentAttnA_Ins_1 | SalVentAttnA |
| 290 | 17Networks_RH_SalVentAttnA_Ins_2 | SalVentAttnA |
| 291 | 17Networks_RH_SalVentAttnA_Ins_3 | SalVentAttnA |
| 292 | 17Networks_RH_SalVentAttnA_Ins_4 | SalVentAttnA |
| 293 | 17Networks_RH_SalVentAttnA_FrOper_1 | SalVentAttnA |
| 294 | 17Networks_RH_SalVentAttnA_FrOper_2 | SalVentAttnA |
| 295 | 17Networks_RH_SalVentAttnA_FrOper_3 | SalVentAttnA |
| 296 | 17Networks_RH_SalVentAttnA_FrMed_1 | SalVentAttnA |
| 297 | 17Networks_RH_SalVentAttnA_ParMed_1 | SalVentAttnA |
| 298 | 17Networks_RH_SalVentAttnA_ParMed_2 | SalVentAttnA |
| 299 | 17Networks_RH_SalVentAttnA_FrMed_2 | SalVentAttnA |
| 300 | 17Networks_RH_SalVentAttnA_ParMed_3 | SalVentAttnA |
| 301 | 17Networks_RH_SalVentAttnA_ParMed_4 | SalVentAttnA |
| 302 | 17Networks_RH_SalVentAttnA_FrMed_3 | SalVentAttnA |
| 304 | 17Networks_RH_SalVentAttnB_IPL_1 | SalVentAttnB |
| 305 | 17Networks_RH_SalVentAttnB_PFClv_1 | SalVentAttnB |
| 306 | 17Networks_RH_SalVentAttnB_PFCl_1 | SalVentAttnB |
| 307 | 17Networks_RH_SalVentAttnB_PFCl_2 | SalVentAttnB |
| 308 | 17Networks_RH_SalVentAttnB_PFCl_3 | SalVentAttnB |
| 309 | 17Networks_RH_SalVentAttnB_Ins_1 | SalVentAttnB |
| 310 | 17Networks_RH_SalVentAttnB_Ins_2 | SalVentAttnB |
| 311 | 17Networks_RH_SalVentAttnB_PFCmp_1 | SalVentAttnB |
| 312 | 17Networks_RH_SalVentAttnB_PFCmp_2 | SalVentAttnB |
| 325 | 17Networks_RH_ContA_IPS_1 | ContA |
| 326 | 17Networks_RH_ContA_IPS_2 | ContA |
| 327 | 17Networks_RH_ContA_IPS_3 | ContA |
| 328 | 17Networks_RH_ContA_IPS_4 | ContA |
| 329 | 17Networks_RH_ContA_PFCd_1 | ContA |
| 330 | 17Networks_RH_ContA_PFCl_1 | ContA |
| 331 | 17Networks_RH_ContA_PFCl_2 | ContA |
| 332 | 17Networks_RH_ContA_PFCl_3 | ContA |
| 333 | 17Networks_RH_ContA_PFCl_4 | ContA |
| 334 | 17Networks_RH_ContA_PFCl_5 | ContA |
| 335 | 17Networks_RH_ContA_Cingm_1 | ContA |
| 338 | 17Networks_RH_ContB_IPL_1 | ContB |
| 340 | 17Networks_RH_ContB_IPL_3 | ContB |
| 341 | 17Networks_RH_ContB_IPL_4 | ContB |
| 342 | 17Networks_RH_ContB_PFCld_1 | ContB |
| 343 | 17Networks_RH_ContB_PFCld_2 | ContB |
| 344 | 17Networks_RH_ContB_PFCld_3 | ContB |
| 345 | 17Networks_RH_ContB_PFCld_4 | ContB |
| 350 | 17Networks_RH_ContB_PFCmp_1 | ContB |
| 351 | 17Networks_RH_ContC_pCun_1 | ContC |
| 352 | 17Networks_RH_ContC_pCun_2 | ContC |
| 353 | 17Networks_RH_ContC_pCun_3 | ContC |
| 354 | 17Networks_RH_ContC_pCun_4 | ContC |
| 355 | 17Networks_RH_ContC_pCun_5 | ContC |
| 356 | 17Networks_RH_ContC_Cingp_1 | ContC |
| 357 | 17Networks_RH_ContC_Cingp_2 | ContC |
| 359 | 17Networks_RH_DefaultA_IPL_1 | DefaultA |
| 360 | 17Networks_RH_DefaultA_IPL_2 | DefaultA |
| 361 | 17Networks_RH_DefaultA_PFCd_1 | DefaultA |
| 362 | 17Networks_RH_DefaultA_PFCd_2 | DefaultA |
| 363 | 17Networks_RH_DefaultA_pCunPCC_1 | DefaultA |
| 364 | 17Networks_RH_DefaultA_pCunPCC_2 | DefaultA |
| 365 | 17Networks_RH_DefaultA_pCunPCC_3 | DefaultA |
| 366 | 17Networks_RH_DefaultA_pCunPCC_4 | DefaultA |
| 367 | 17Networks_RH_DefaultA_pCunPCC_5 | DefaultA |
| 370 | 17Networks_RH_DefaultA_PFCm_3 | DefaultA |
| 371 | 17Networks_RH_DefaultA_PFCm_4 | DefaultA |
| 373 | 17Networks_RH_DefaultA_PFCm_6 | DefaultA |
| 375 | 17Networks_RH_DefaultB_Temp_2 | DefaultB |
| 377 | 17Networks_RH_DefaultB_PFCd_1 | DefaultB |
| 378 | 17Networks_RH_DefaultB_PFCd_2 | DefaultB |
| 379 | 17Networks_RH_DefaultB_PFCd_3 | DefaultB |
| 382 | 17Networks_RH_DefaultB_PFCv_1 | DefaultB |
| 383 | 17Networks_RH_DefaultB_PFCv_2 | DefaultB |
| 384 | 17Networks_RH_DefaultB_PFCv_3 | DefaultB |
| 385 | 17Networks_RH_DefaultC_IPL_1 | DefaultC |
| 387 | 17Networks_RH_DefaultC_Rsp_1 | DefaultC |
| 388 | 17Networks_RH_DefaultC_Rsp_2 | DefaultC |
| 390 | 17Networks_RH_DefaultC_PHC_2 | DefaultC |
| 392 | 17Networks_RH_TempPar_2 | TempPar |
| 393 | 17Networks_RH_TempPar_3 | TempPar |
| 394 | 17Networks_RH_TempPar_4 | TempPar |
| 395 | 17Networks_RH_TempPar_5 | TempPar |
| 396 | 17Networks_RH_TempPar_6 | TempPar |
| 397 | 17Networks_RH_TempPar_7 | TempPar |
| 398 | 17Networks_RH_TempPar_8 | TempPar |
| 399 | 17Networks_RH_TempPar_9 | TempPar |
| 400 | 17Networks_RH_TempPar_10 | TempPar |
| n.a. | FreeSurfer_Left-Thalamus | Sub-Cortical |
| n.a. | FreeSurfer_Left-Caudate | Sub-Cortical |
| n.a. | FreeSurfer_Left-Putamen | Sub-Cortical |
| n.a. | FreeSurfer_Left-Pallidum | Sub-Cortical |
| n.a. | FreeSurfer_Left-Hippocampus | Sub-Cortical |
| n.a. | FreeSurfer_Right-Thalamus | Sub-Cortical |
| n.a. | FreeSurfer_Right-Caudate | Sub-Cortical |
| n.a. | FreeSurfer_Right-Putamen | Sub-Cortical |
| n.a. | FreeSurfer_Right-Pallidum | Sub-Cortical |
| n.a. | FreeSurfer_Right-Hippocampus | Sub-Cortical |
| n.a. | Buckner2011_17Networks_4 | Cerebellum |
| n.a. | lamygdala | Sub-Cortical |
| n.a. | lventralstriatum | Sub-Cortical |
| n.a. | ramygdala | Sub-Cortical |
| n.a. | rventralstriatum | Sub-Cortical |

**Supplementary Table S4.** Overview describing differences in age, sex and mean framewise displacement (FD) between compared groups for each contrast separately. Reported p-values for differences in age and mean FD were obtained using t-tests, and Chi-squared tests were used to asses differences in sex.

| **Contrast** | **Compared groups** | **N** | | **Age** | | | **Mean FD** | | | **% Male** | | |
| --- | --- | --- | --- | --- | --- | --- | --- | --- | --- | --- | --- | --- |
|  |  | **Group 1** | **Group 2** | **Group 1** | **Group 2** | **p-value** | **Group 1** | **Group 2** | **p-value** | **Group 1** | **Group 2** | **p-value** |
| **1** | OCD patients vs. healthy controls – both age groups combined (adult and pediatric samples) | 1028 | 1024 | 27.98 | 29.55 | <0.001 | 0.10 | 0.11 | <0.001 | 53 | 47 | 0.008 |
| **2** | OCD patients vs. healthy controls – adult samples | 101 | 103 | 13.97 | 14.14 | 0.62 | 0.11 | 0.11 | 0.659 | 50 | 50 | 0.998 |
| **3** | OCD patients vs. healthy controls – pediatric samples | 914 | 903 | 29.68 | 31.55 | <0.001 | 0.10 | 0.11 | <0.001 | 53 | 46 | 0.003 |
| **4** | Medicated OCD patients vs. healthy controls – both age groups combined | 509 | 342 | 26.52 | 27.74 | 0.079 | 0.10 | 0.11 | 0.044 | 54 | 44 | 0.002 |
| **5** | Unmedicated OCD patients vs. healthy controls – both age groups combined | 420 | 356 | 28.94 | 30.29 | 0.054 | 0.11 | 0.12 | 0.153 | 56 | 48 | 0.027 |
| **6** | Medicated OCD patients vs. Unmedicated OCD patients – both age groups combined | 193 | 158 | 31.75 | 31.78 | 0.971 | 0.12 | 0.12 | 0.716 | 49 | 45 | 0.424 |
| **7** | Early age of onset OCD patients vs. healthy controls – adult samples | 383 | 198 | 28.98 | 29.89 | 0.234 | 0.11 | 0.12 | 0.009 | 52 | 51 | 0.651 |
| **8** | Late age of onset OCD patients vs. healthy controls – adult samples | 473 | 300 | 28.54 | 31.89 | <0.001 | 0.11 | 0.11 | 0.390 | 53 | 43 | 0.006 |
| **9** | Early age of onset OCD patients vs. Late age of onset OCD patients – adults samples | 244 | 268 | 28.77 | 32.79 | <0.001 | 0.11 | 0.10 | 0.358 | 56 | 44 | 0.010 |
| **10** | Low severity OCD patients vs. healthy controls – both age groups combined | 598 | 376 | 26.73 | 27.19 | 0.494 | 0.11 | 0.11 | 0.067 | 52 | 50 | 0.611 |
| **11** | High severity OCD patients vs. healthy controls – both age groups combined | 470 | 281 | 27.99 | 31.06 | <0.001 | 0.10 | 0.12 | 0.001 | 52 | 42 | 0.010 |
| **12** | Low severity OCD patients vs. High severity OCD patients – both age groups combined | 391 | 376 | 29.17 | 31.36 | <0.001 | 0.11 | 0.11 | 0.989 | 53 | 44 | 0.010 |

**Supplementary Table S5**. Overview of obtained performances for diagnosis classifications and within-patient classifications using the different rsfMRI features. Asterisks depict significance (Bonferroni corrected with alpha=0.05/24) assessed with label permutation tests. AUC = area under the receiver operating characteristic curve; PPV = positive prediction value; NPV = negative prediction value.

| **Classification** | **Sample filter** | **Age Group** | **Feature** | **AUC** (95% CI) | **p-value** (uncorrected) | **p-value** (Bonferroni corrected) | **Balanced Accuracy** | **Sensitivity** | **Specificity** | **PPV** | **NPV** |
| --- | --- | --- | --- | --- | --- | --- | --- | --- | --- | --- | --- |
| Diagnosis | OCD vs HC | adult | ROI-to-ROI-FC | 0.684* (0.659 - 0.708) | 0.001 | 0.02398 | 0.634 | 0.658 | 0.609 | 0.625 | 0.644 |
| Diagnosis | OCD vs HC | adult | fALFF-ROI | 0.614* (0.588 - 0.640) | 0.001 | 0.02398 | 0.587 | 0.631 | 0.543 | 0.577 | 0.598 |
| Diagnosis | OCD vs HC | adult | fALFF-network | 0.596* (0.570 - 0.622) | 0.001 | 0.02398 | 0.568 | 0.538 | 0.597 | 0.569 | 0.567 |
| Diagnosis | OCD vs HC | adult | network-FC | 0.641* (0.615 - 0.666) | 0.001 | 0.02398 | 0.604 | 0.648 | 0.561 | 0.593 | 0.617 |
| Diagnosis | OCD vs HC | adult | reHo-ROI | 0.631* (0.606 - 0.657) | 0.001 | 0.02398 | 0.592 | 0.579 | 0.605 | 0.592 | 0.593 |
| Diagnosis | OCD vs HC | adult | reHo-network | 0.565* (0.539 - 0.591) | 0.001 | 0.02398 | 0.548 | 0.654 | 0.442 | 0.536 | 0.564 |
| Diagnosis | OCD vs HC | pediatric | ROI-to-ROI-FC | 0.552 (0.474 - 0.631) | 0.24975 | 1 | 0.544 | 0.596 | 0.492 | 0.547 | 0.545 |
| Diagnosis | OCD vs HC | pediatric | fALFF-ROI | 0.606 (0.528 - 0.683) | 0.03896 | 0.93506 | 0.576 | 0.57 | 0.581 | 0.585 | 0.572 |
| Diagnosis | OCD vs HC | pediatric | fALFF-network | 0.608 (0.530 - 0.685) | 0.02697 | 0.64735 | 0.574 | 0.54 | 0.608 | 0.583 | 0.565 |
| Diagnosis | OCD vs HC | pediatric | network-FC | 0.542 (0.462 - 0.621) | 0.23976 | 1 | 0.542 | 0.567 | 0.516 | 0.508 | 0.544 |
| Diagnosis | OCD vs HC | pediatric | reHo-ROI | 0.615 (0.537 - 0.692) | 0.02897 | 0.6953 | 0.589 | 0.537 | 0.642 | 0.609 | 0.577 |
| Diagnosis | OCD vs HC | pediatric | reHo-network | 0.602 (0.524 - 0.679) | 0.05495 | 1 | 0.589 | 0.552 | 0.626 | 0.596 | 0.58 |
| Diagnosis | OCD vs HC | pooled | ROI-to-ROI-FC | 0.673* (0.650 - 0.696) | 0.001 | 0.02398 | 0.626 | 0.655 | 0.598 | 0.619 | 0.635 |
| Diagnosis | OCD vs HC | pooled | fALFF-ROI | 0.611* (0.587 - 0.636) | 0.001 | 0.02398 | 0.585 | 0.632 | 0.538 | 0.577 | 0.595 |
| Diagnosis | OCD vs HC | pooled | fALFF-network | 0.598* (0.574 - 0.623) | 0.001 | 0.02398 | 0.568 | 0.544 | 0.591 | 0.57 | 0.566 |
| Diagnosis | OCD vs HC | pooled | network-FC | 0.633* (0.609 - 0.657) | 0.001 | 0.02398 | 0.596 | 0.644 | 0.548 | 0.587 | 0.607 |
| Diagnosis | OCD vs HC | pooled | reHo-ROI | 0.625* (0.601 - 0.649) | 0.001 | 0.02398 | 0.59 | 0.584 | 0.596 | 0.591 | 0.59 |
| Diagnosis | OCD vs HC | pooled | reHo-network | 0.567* (0.542 - 0.592) | 0.001 | 0.02398 | 0.552 | 0.654 | 0.45 | 0.543 | 0.567 |
| Diagnosis | Med OCD vs HC | pooled | ROI-to-ROI-FC | 0.702* (0.666 - 0.737) | 0.001 | 0.02398 | 0.647 | 0.62 | 0.674 | 0.562 | 0.727 |
| Diagnosis | Unmed OCD vs HC | pooled | ROI-to-ROI-FC | 0.608* (0.568 - 0.647) | 0.001 | 0.02398 | 0.572 | 0.517 | 0.626 | 0.541 | 0.605 |
| Diagnosis | Med OCD vs HC | pooled | fALFF-ROI | 0.650* (0.613 - 0.687) | 0.001 | 0.02398 | 0.61 | 0.581 | 0.639 | 0.52 | 0.695 |
| Diagnosis | Unmed OCD vs HC | pooled | fALFF-ROI | 0.585* (0.545 - 0.625) | 0.001 | 0.02398 | 0.555 | 0.506 | 0.605 | 0.525 | 0.586 |
| Diagnosis | Med OCD vs HC | pooled | fALFF-network | 0.635* (0.597 - 0.672) | 0.001 | 0.02398 | 0.595 | 0.52 | 0.67 | 0.514 | 0.676 |
| Diagnosis | Unmed OCD vs HC | pooled | fALFF-network | 0.587* (0.547 - 0.627) | 0.002 | 0.04795 | 0.558 | 0.439 | 0.677 | 0.537 | 0.588 |
| Diagnosis | Med OCD vs HC | pooled | network-FC | 0.648* (0.611 - 0.685) | 0.001 | 0.02398 | 0.613 | 0.63 | 0.596 | 0.512 | 0.707 |
| Diagnosis | Unmed OCD vs HC | pooled | network-FC | 0.534 (0.493 - 0.574) | 0.11089 | 1 | 0.517 | 0.524 | 0.511 | 0.462 | 0.559 |
| Diagnosis | Med OCD vs HC | pooled | reHo-ROI | 0.638* (0.601 - 0.675) | 0.001 | 0.02398 | 0.595 | 0.584 | 0.606 | 0.499 | 0.685 |
| Diagnosis | Unmed OCD vs HC | pooled | reHo-ROI | 0.580* (0.540 - 0.621) | 0.001 | 0.02398 | 0.558 | 0.535 | 0.58 | 0.52 | 0.596 |
| Diagnosis | Med OCD vs HC | pooled | reHo-network | 0.622* (0.585 - 0.660) | 0.001 | 0.02398 | 0.591 | 0.585 | 0.596 | 0.494 | 0.682 |
| Diagnosis | Unmed OCD vs HC | pooled | reHo-network | 0.515 (0.474 - 0.555) | 0.49151 | 1 | 0.504 | 0.431 | 0.577 | 0.366 | 0.468 |
| Diagnosis | Early AO OCD vs HC | pooled | ROI-to-ROI-FC | 0.651* (0.612 - 0.689) | 0.001 | 0.02398 | 0.6 | 0.516 | 0.684 | 0.509 | 0.692 |
| Diagnosis | Late AO OCD vs HC | pooled | ROI-to-ROI-FC | 0.682* (0.644 - 0.720) | 0.001 | 0.02398 | 0.632 | 0.535 | 0.729 | 0.558 | 0.712 |
| Diagnosis | Early AO OCD vs HC | pooled | fALFF-ROI | 0.598* (0.558 - 0.638) | 0.001 | 0.02398 | 0.569 | 0.568 | 0.569 | 0.454 | 0.677 |
| Diagnosis | Late AO OCD vs HC | pooled | fALFF-ROI | 0.656* (0.617 - 0.694) | 0.001 | 0.02398 | 0.614 | 0.573 | 0.654 | 0.514 | 0.707 |
| Diagnosis | Early AO OCD vs HC | pooled | fALFF-network | 0.571 (0.531 - 0.611) | 0.00599 | 0.14386 | 0.553 | 0.547 | 0.559 | 0.441 | 0.662 |
| Diagnosis | Late AO OCD vs HC | pooled | fALFF-network | 0.618* (0.578 - 0.657) | 0.001 | 0.02398 | 0.581 | 0.57 | 0.592 | 0.471 | 0.685 |
| Diagnosis | Early AO OCD vs HC | pooled | network-FC | 0.601* (0.562 - 0.641) | 0.001 | 0.02398 | 0.577 | 0.57 | 0.584 | 0.465 | 0.683 |
| Diagnosis | Late AO OCD vs HC | pooled | network-FC | 0.637* (0.598 - 0.676) | 0.001 | 0.02398 | 0.601 | 0.597 | 0.605 | 0.491 | 0.703 |
| Diagnosis | Early AO OCD vs HC | pooled | reHo-ROI | 0.612* (0.572 - 0.651) | 0.001 | 0.02398 | 0.581 | 0.55 | 0.613 | 0.473 | 0.684 |
| Diagnosis | Late AO OCD vs HC | pooled | reHo-ROI | 0.612* (0.573 - 0.652) | 0.001 | 0.02398 | 0.584 | 0.52 | 0.648 | 0.486 | 0.68 |
| Diagnosis | Early AO OCD vs HC | pooled | reHo-network | 0.576 (0.536 - 0.616) | 0.003 | 0.07193 | 0.559 | 0.556 | 0.563 | 0.445 | 0.668 |
| Diagnosis | Late AO OCD vs HC | pooled | reHo-network | 0.620* (0.581 - 0.659) | 0.001 | 0.02398 | 0.584 | 0.553 | 0.616 | 0.479 | 0.685 |
| Diagnosis | High Sev OCD vs HC | pooled | ROI-to-ROI-FC | 0.660* (0.620 - 0.699) | 0.001 | 0.02398 | 0.613 | 0.516 | 0.71 | 0.517 | 0.711 |
| Diagnosis | Low Sev OCD vs HC | pooled | ROI-to-ROI-FC | 0.629* (0.594 - 0.664) | 0.001 | 0.02398 | 0.583 | 0.473 | 0.694 | 0.494 | 0.677 |
| Diagnosis | High Sev OCD vs HC | pooled | fALFF-ROI | 0.612* (0.572 - 0.652) | 0.001 | 0.02398 | 0.573 | 0.572 | 0.574 | 0.445 | 0.693 |
| Diagnosis | Low Sev OCD vs HC | pooled | fALFF-ROI | 0.581* (0.545 - 0.617) | 0.001 | 0.02398 | 0.558 | 0.624 | 0.492 | 0.437 | 0.678 |
| Diagnosis | High Sev OCD vs HC | pooled | fALFF-network | 0.614* (0.573 - 0.654) | 0.001 | 0.02398 | 0.584 | 0.59 | 0.578 | 0.456 | 0.703 |
| Diagnosis | Low Sev OCD vs HC | pooled | fALFF-network | 0.567 (0.531 - 0.603) | 0.00599 | 0.14386 | 0.554 | 0.64 | 0.468 | 0.431 | 0.675 |
| Diagnosis | High Sev OCD vs HC | pooled | network-FC | 0.623* (0.583 - 0.663) | 0.001 | 0.02398 | 0.594 | 0.593 | 0.595 | 0.468 | 0.71 |
| Diagnosis | Low Sev OCD vs HC | pooled | network-FC | 0.605* (0.570 - 0.641) | 0.001 | 0.02398 | 0.577 | 0.575 | 0.58 | 0.464 | 0.684 |
| Diagnosis | High Sev OCD vs HC | pooled | reHo-ROI | 0.625* (0.585 - 0.665) | 0.001 | 0.02398 | 0.591 | 0.535 | 0.648 | 0.476 | 0.701 |
| Diagnosis | Low Sev OCD vs HC | pooled | reHo-ROI | 0.590* (0.554 - 0.626) | 0.001 | 0.02398 | 0.568 | 0.525 | 0.612 | 0.46 | 0.671 |
| Diagnosis | High Sev OCD vs HC | pooled | reHo-network | 0.538 (0.497 - 0.579) | 0.21678 | 1 | 0.517 | 0.526 | 0.508 | 0.381 | 0.56 |
| Diagnosis | Low Sev OCD vs HC | pooled | reHo-network | 0.546 (0.510 - 0.582) | 0.09191 | 1 | 0.529 | 0.583 | 0.476 | 0.411 | 0.646 |
| Medication status | Med vs Unmed OCD | pooled | ROI-to-ROI-FC | 0.563 (0.503 - 0.623) | 0.07293 | 1 | 0.55 | 0.474 | 0.626 | 0.51 | 0.593 |
| Medication status | Med vs Unmed OCD | pooled | fALFF-ROI | 0.644* (0.586 - 0.702) | 0.001 | 0.02398 | 0.591 | 0.563 | 0.618 | 0.547 | 0.636 |
| Medication status | Med vs Unmed OCD | pooled | fALFF-network | 0.625* (0.566 - 0.683) | 0.001 | 0.02398 | 0.592 | 0.622 | 0.562 | 0.538 | 0.647 |
| Medication status | Med vs Unmed OCD | pooled | network-FC | 0.524 (0.463 - 0.584) | 0.29171 | 1 | 0.505 | 0.441 | 0.57 | 0.445 | 0.539 |
| Medication status | Med vs Unmed OCD | pooled | reHo-ROI | 0.623* (0.564 - 0.681) | 0.002 | 0.04795 | 0.608 | 0.574 | 0.642 | 0.57 | 0.648 |
| Medication status | Med vs Unmed OCD | pooled | reHo-network | 0.636* (0.578 - 0.694) | 0.001 | 0.02398 | 0.604 | 0.622 | 0.585 | 0.553 | 0.653 |
| Age of Onset | Early AO vs Late AO OCD | pooled | ROI-to-ROI-FC | 0.615* (0.566 - 0.664) | 0.001 | 0.02398 | 0.584 | 0.622 | 0.546 | 0.601 | 0.569 |
| Age of Onset | Early AO vs Late AO OCD | pooled | fALFF-ROI | 0.597 (0.548 - 0.646) | 0.003 | 0.07193 | 0.57 | 0.62 | 0.52 | 0.588 | 0.555 |
| Age of Onset | Early AO vs Late AO OCD | pooled | fALFF-network | 0.589 (0.540 - 0.638) | 0.00599 | 0.14386 | 0.567 | 0.639 | 0.496 | 0.582 | 0.556 |
| Age of Onset | Early AO vs Late AO OCD | pooled | network-FC | 0.584 (0.534 - 0.633) | 0.00799 | 0.19181 | 0.557 | 0.56 | 0.554 | 0.581 | 0.534 |
| Age of Onset | Early AO vs Late AO OCD | pooled | reHo-ROI | 0.599* (0.550 - 0.648) | 0.002 | 0.04795 | 0.573 | 0.679 | 0.468 | 0.583 | 0.578 |
| Age of Onset | Early AO vs Late AO OCD | pooled | reHo-network | 0.624 (0.575 - 0.672) | 0.003 | 0.07193 | 0.584 | 0.659 | 0.508 | 0.596 | 0.578 |
| Severity | Low Sev vs High Sev OCD | pooled | ROI-to-ROI-FC | 0.510 (0.469 - 0.550) | 0.50749 | 1 | 0.507 | 0.502 | 0.512 | 0.497 | 0.517 |
| Severity | Low Sev vs High Sev OCD | pooled | fALFF-ROI | 0.544 (0.503 - 0.584) | 0.07692 | 1 | 0.538 | 0.501 | 0.576 | 0.532 | 0.546 |
| Severity | Low Sev vs High Sev OCD | pooled | fALFF-network | 0.574* (0.533 - 0.614) | 0.002 | 0.04795 | 0.551 | 0.566 | 0.537 | 0.54 | 0.563 |
| Severity | Low Sev vs High Sev OCD | pooled | network-FC | 0.511 (0.470 - 0.552) | 0.44955 | 1 | 0.498 | 0.663 | 0.333 | 0.464 | 0.49 |
| Severity | Low Sev vs High Sev OCD | pooled | reHo-ROI | 0.573 (0.533 - 0.614) | 0.00999 | 0.23976 | 0.556 | 0.505 | 0.606 | 0.552 | 0.561 |
| Severity | Low Sev vs High Sev OCD | pooled | reHo-network | 0.519 (0.478 - 0.559) | 0.49151 | 1 | 0.502 | 0.544 | 0.459 | 0.453 | 0.44 |


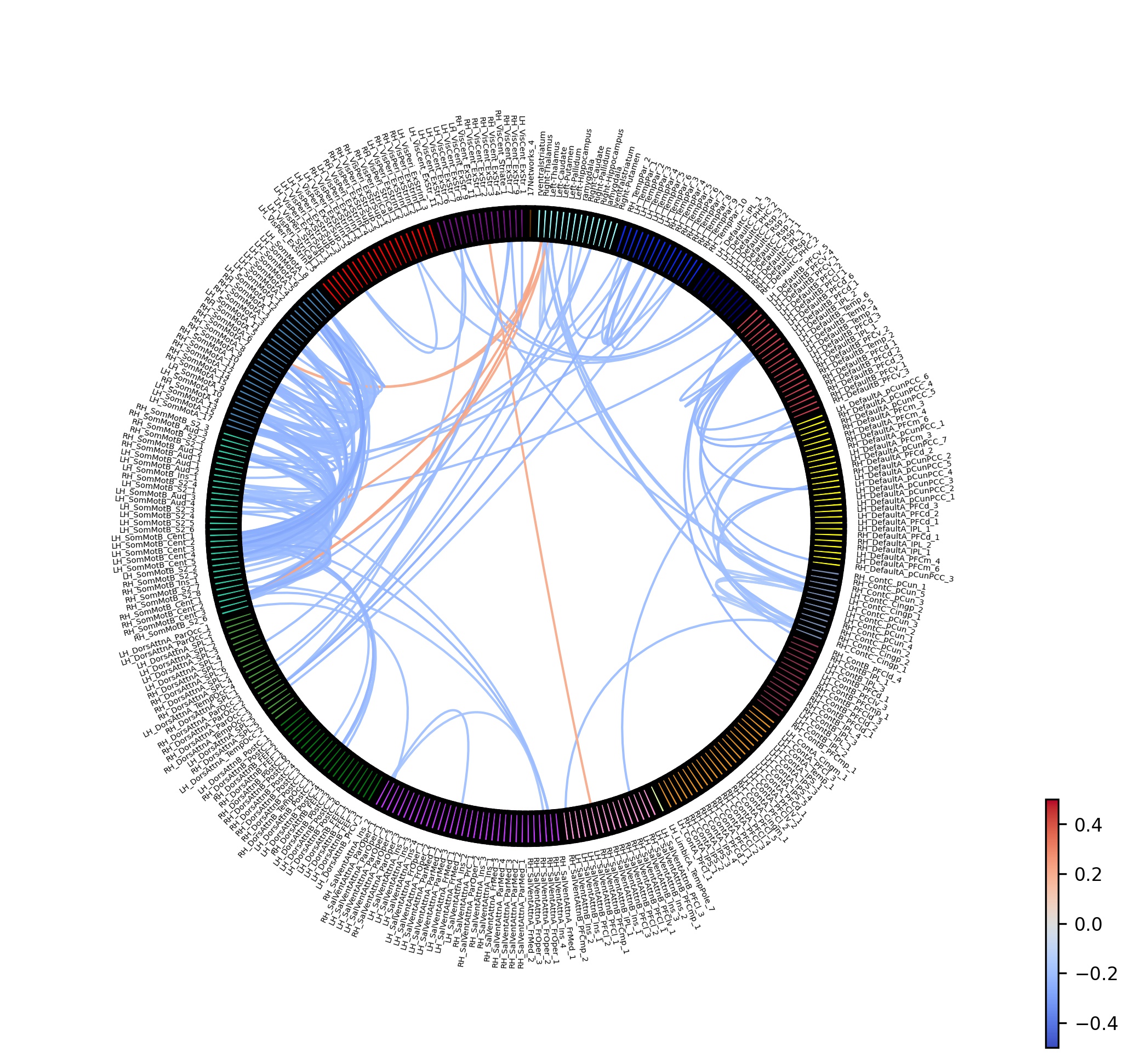


**Supplementary Figure 2.** Effect sizes (Cohen’s *d*) for group differences in ROI-to-ROI functional connectivity between OCD patients and controls from pooled samples across age groups.


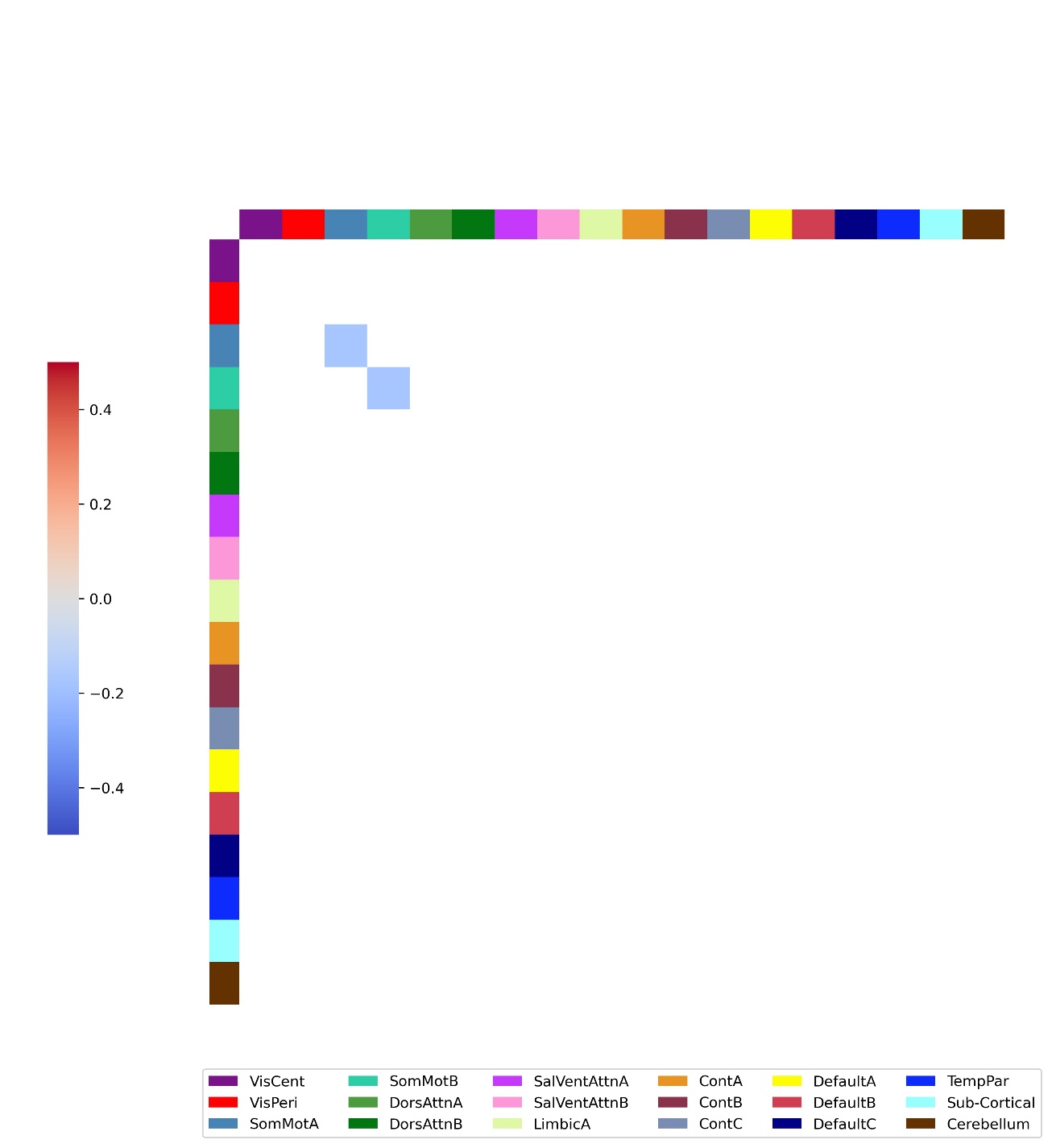


**Supplementary Figure 3.** Effect sizes (Cohen’s *d*) for group differences in between and within-network functional connectivity between OCD patients and controls from pooled samples across age groups.


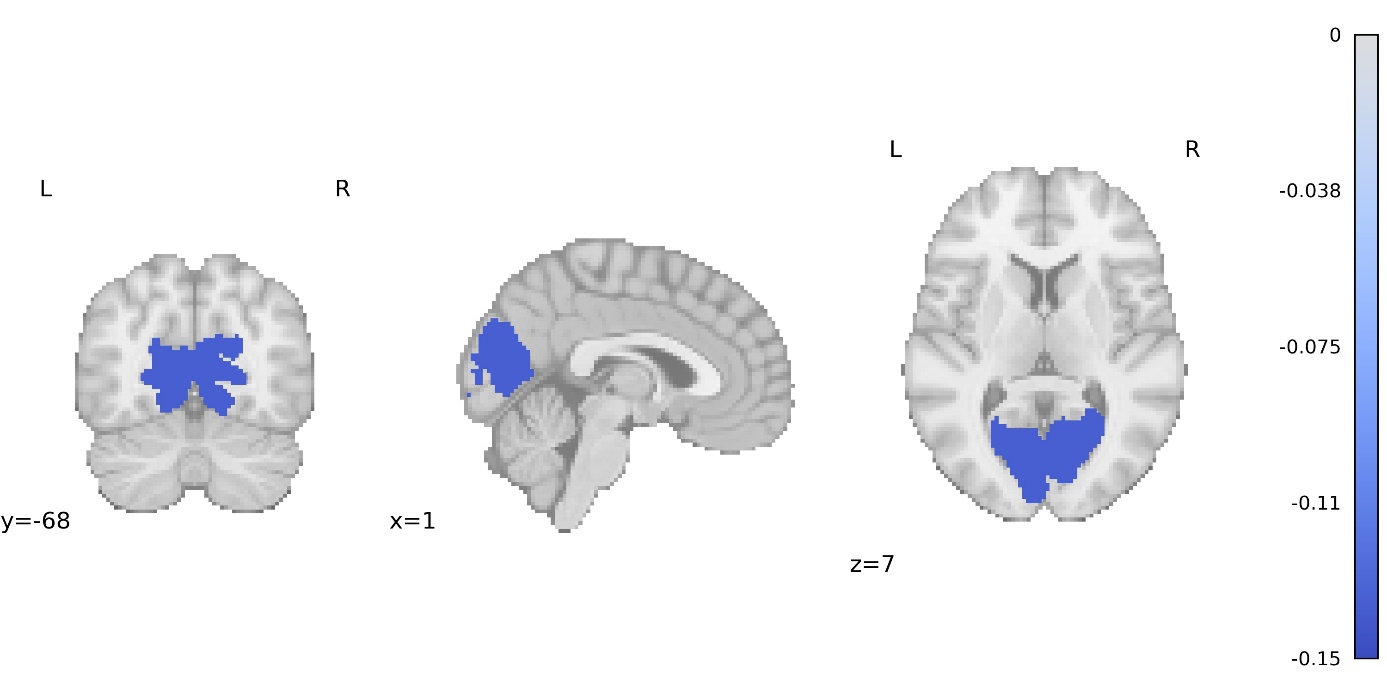


**Supplementary Figure 4.** Effect sizes (Cohen’s *d*) for group differences in network-wise reHo between OCD patients and controls from pooled samples across age groups.


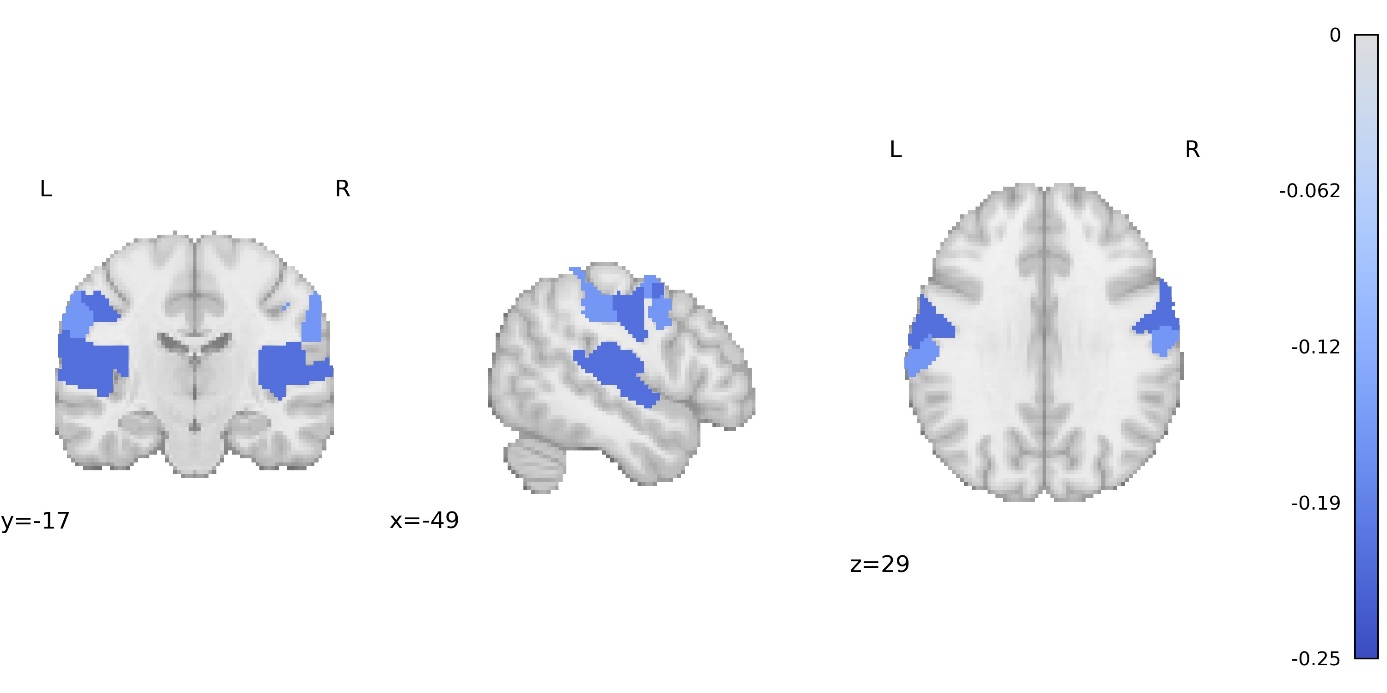


**Supplementary Figure 5.** Effect sizes (Cohen’s *d*) for group differences in network-wise fALFF between OCD patients and controls from pooled samples across age groups.


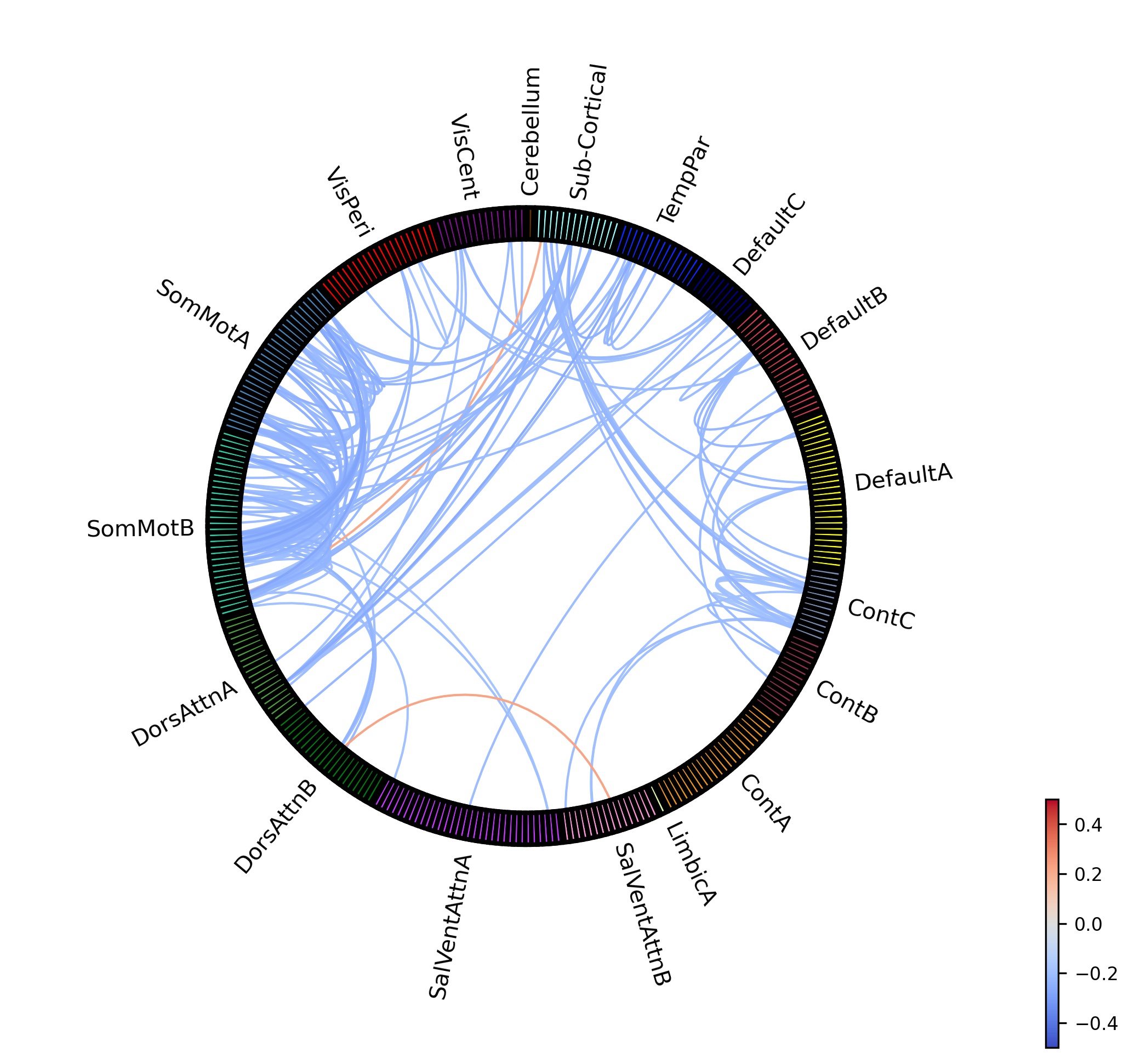


**Supplementary Figure 6.** Effect sizes (Cohen’s *d*) for group differences in ROI-to-ROI functional connectivity between adult OCD patients and controls.
TempPar = Temporal Parietal, Cont = Frontoparietal Control, SalVentAttn = Salience/Ventral Attention, DorsAttn = Dorsal Attention, SomMot = Sensorimotor, VisCent = Visual Central (Visual A), VisPeri = Visual Peripheral (Visual B).


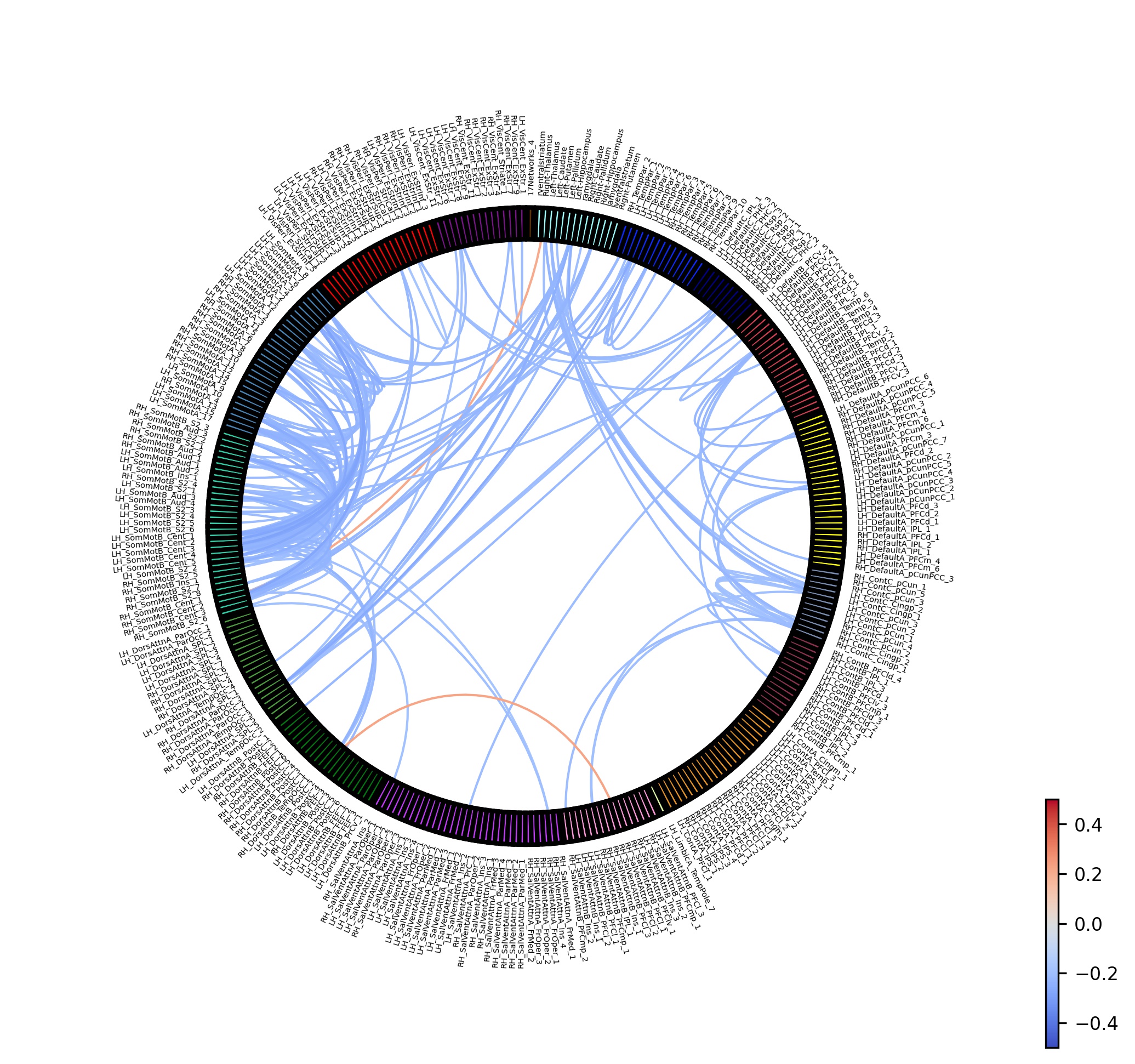


**Supplementary Figure 7.** Effect sizes (Cohen’s *d*) for group differences in ROI-to-ROI functional connectivity between adult OCD patients and controls.


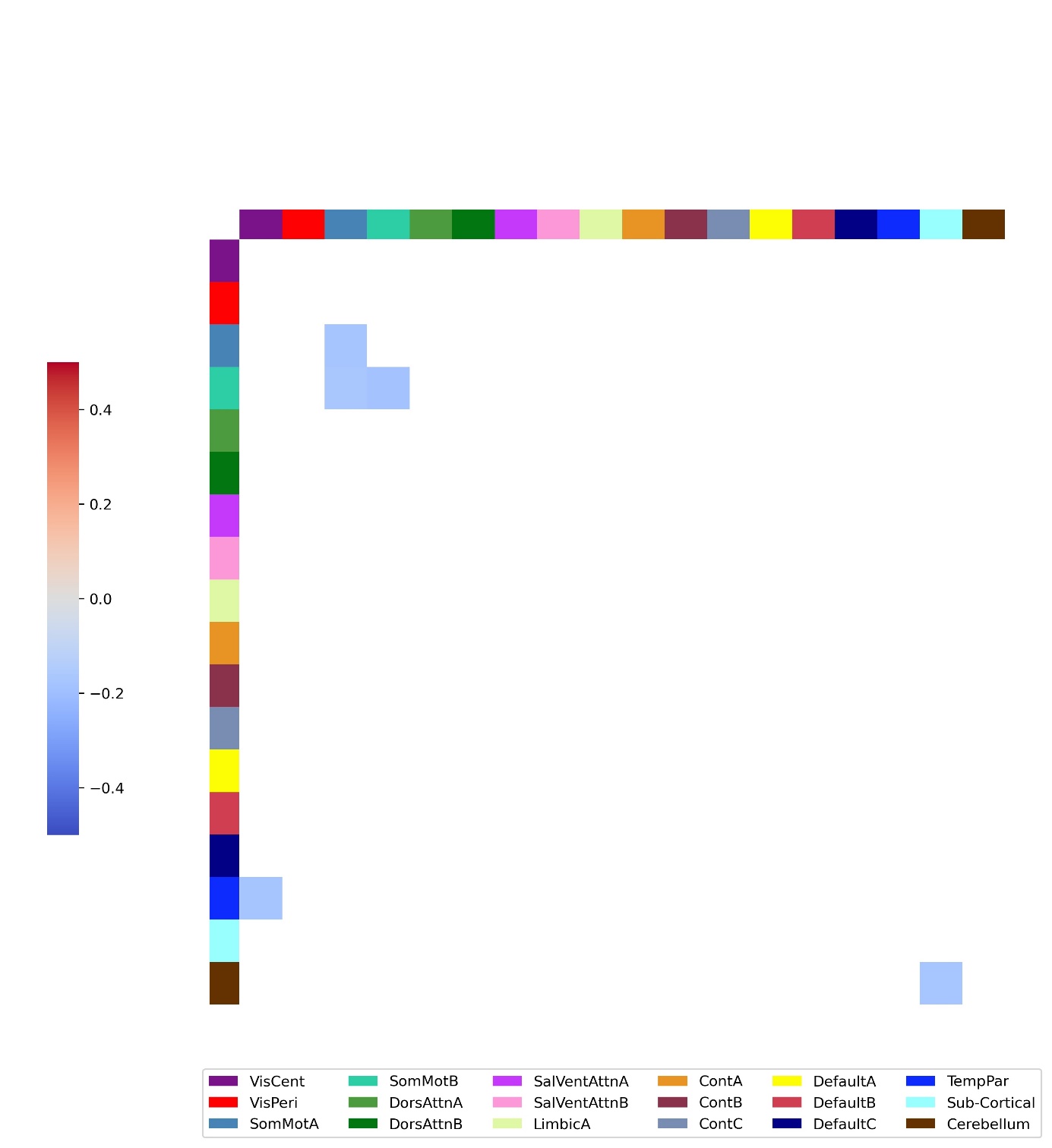


**Supplementary Figure 8.** Effect sizes for group differences in between and within-network functional connectivity between adult OCD patients and controls.


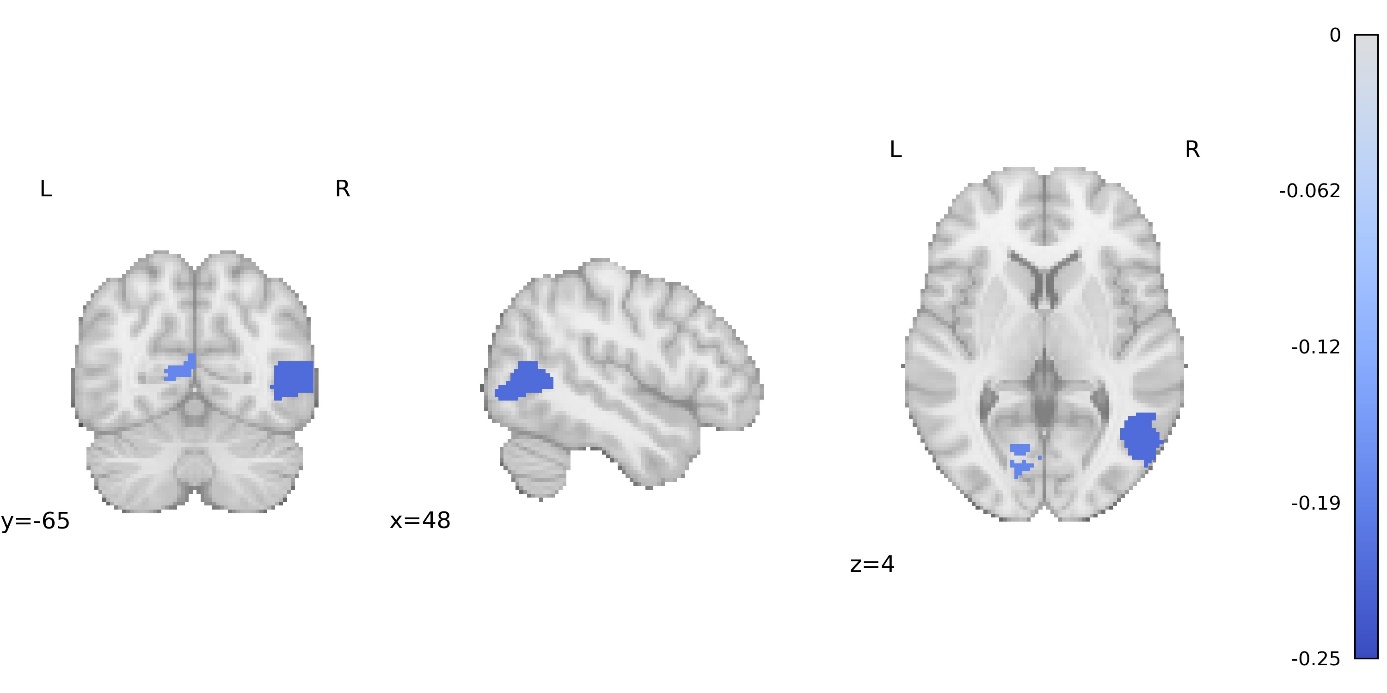


**Supplementary Figure 9.** Effect sizes for group differences in regional reHo between adult OCD patients and controls.


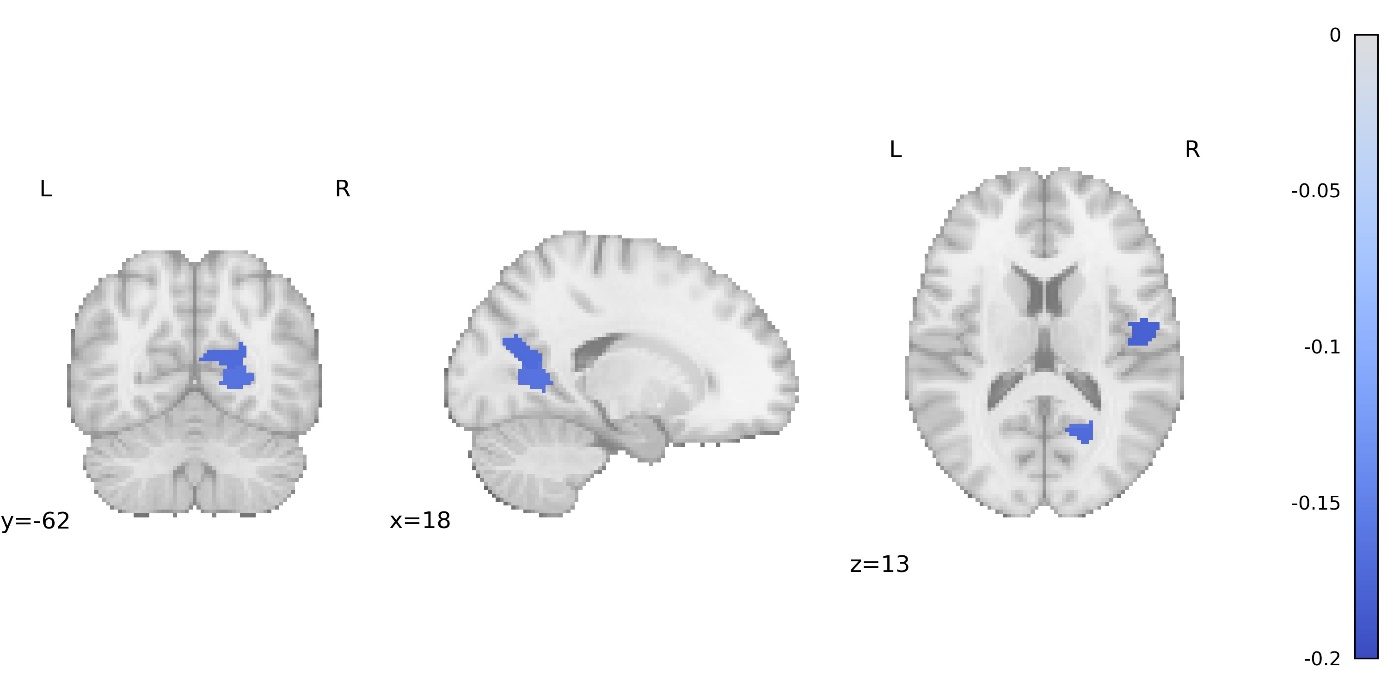


**Supplementary Figure 10.** Effect sizes for group differences in regional fALFF between adult OCD patients and controls.


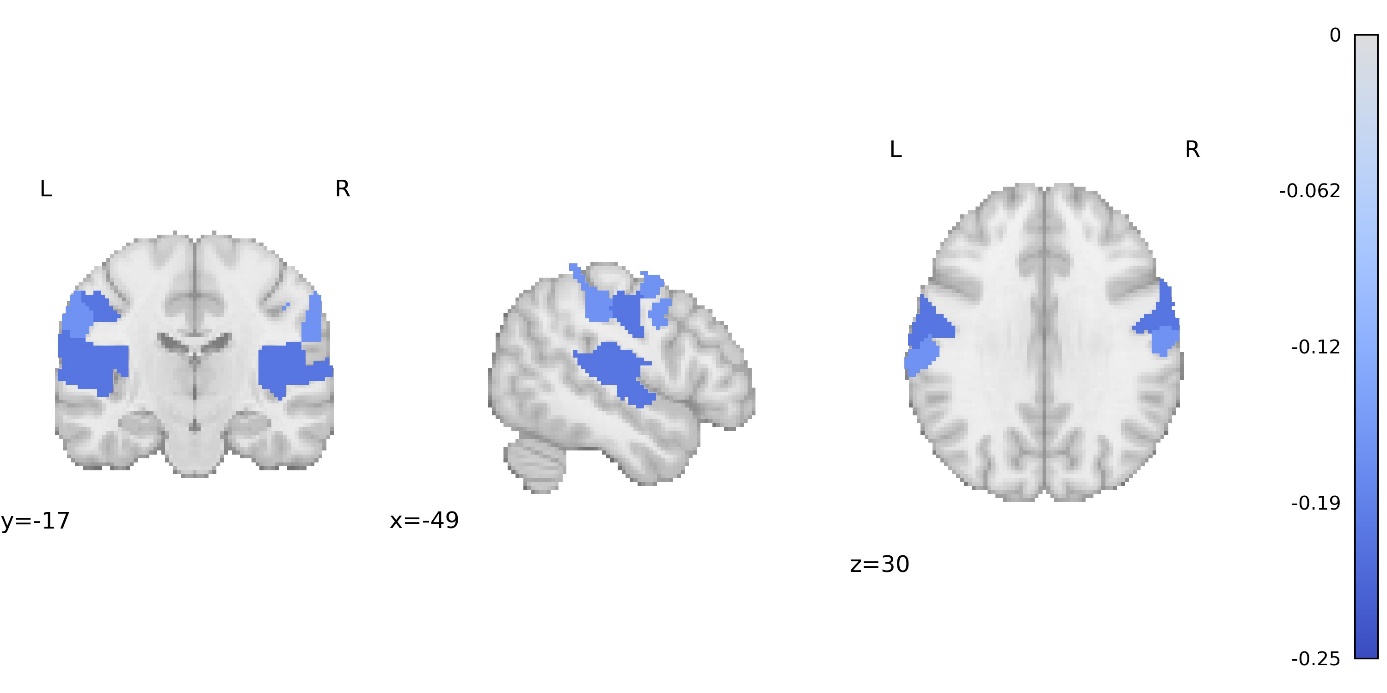


**Supplementary Figure 11.** Effect sizes for group differences in network-wise fALFF between adult OCD patients and controls.


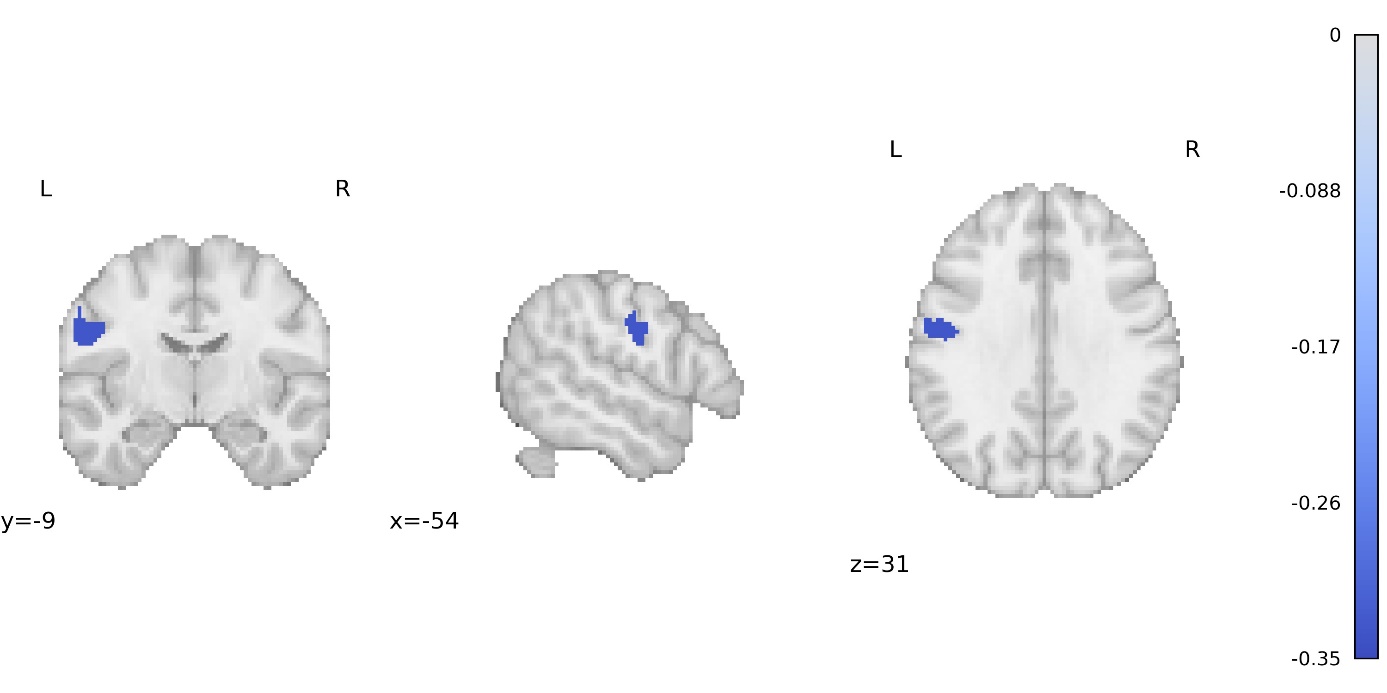


**Supplementary Figure 12.** Effect size for group difference in regional reHo between medicated OCD patients and controls.


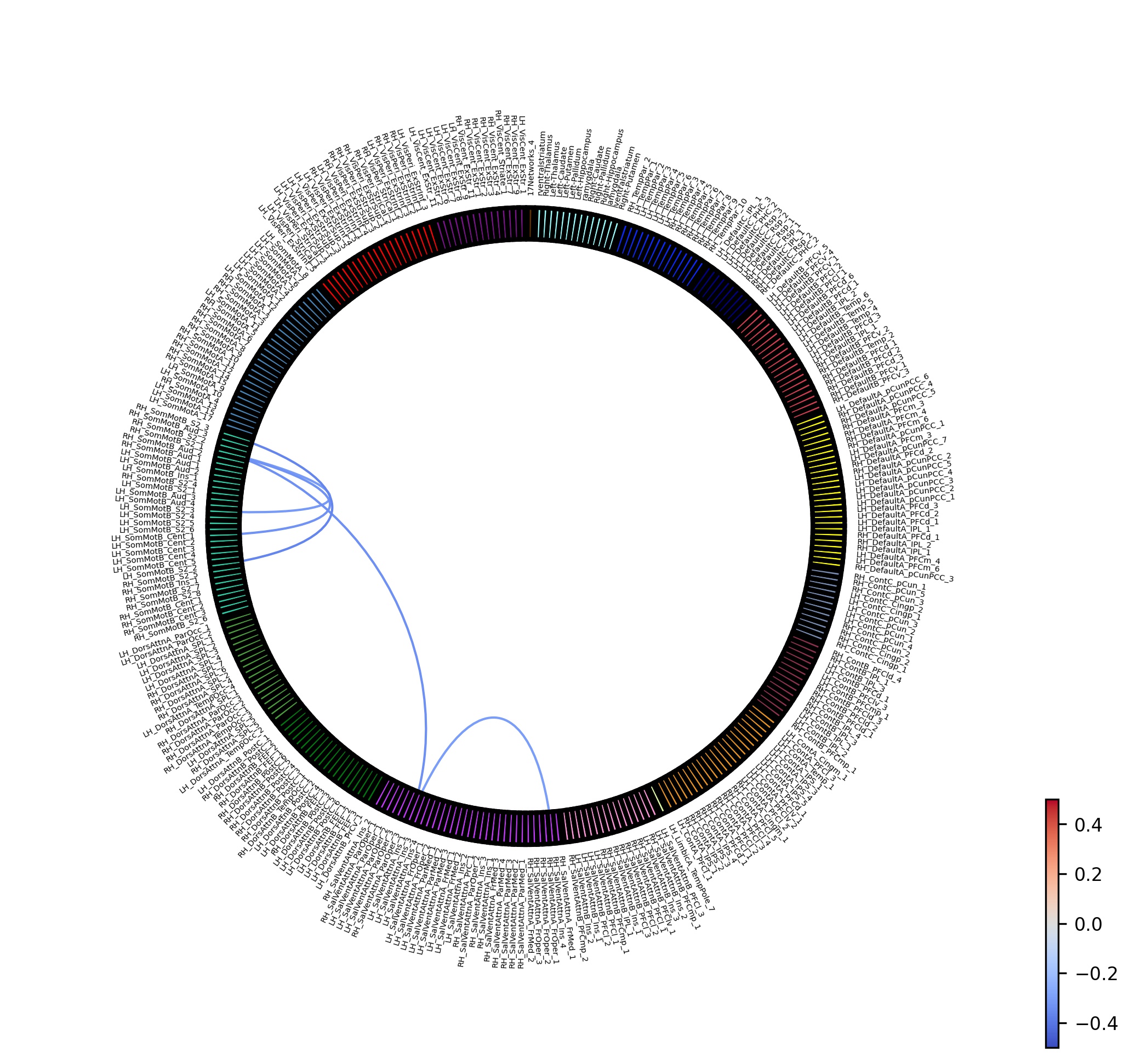


**Supplementary Figure 13.** Effect sizes (Cohen’s *d*) for group differences in ROI-to-ROI functional connectivity between high-severity OCD patients and controls.


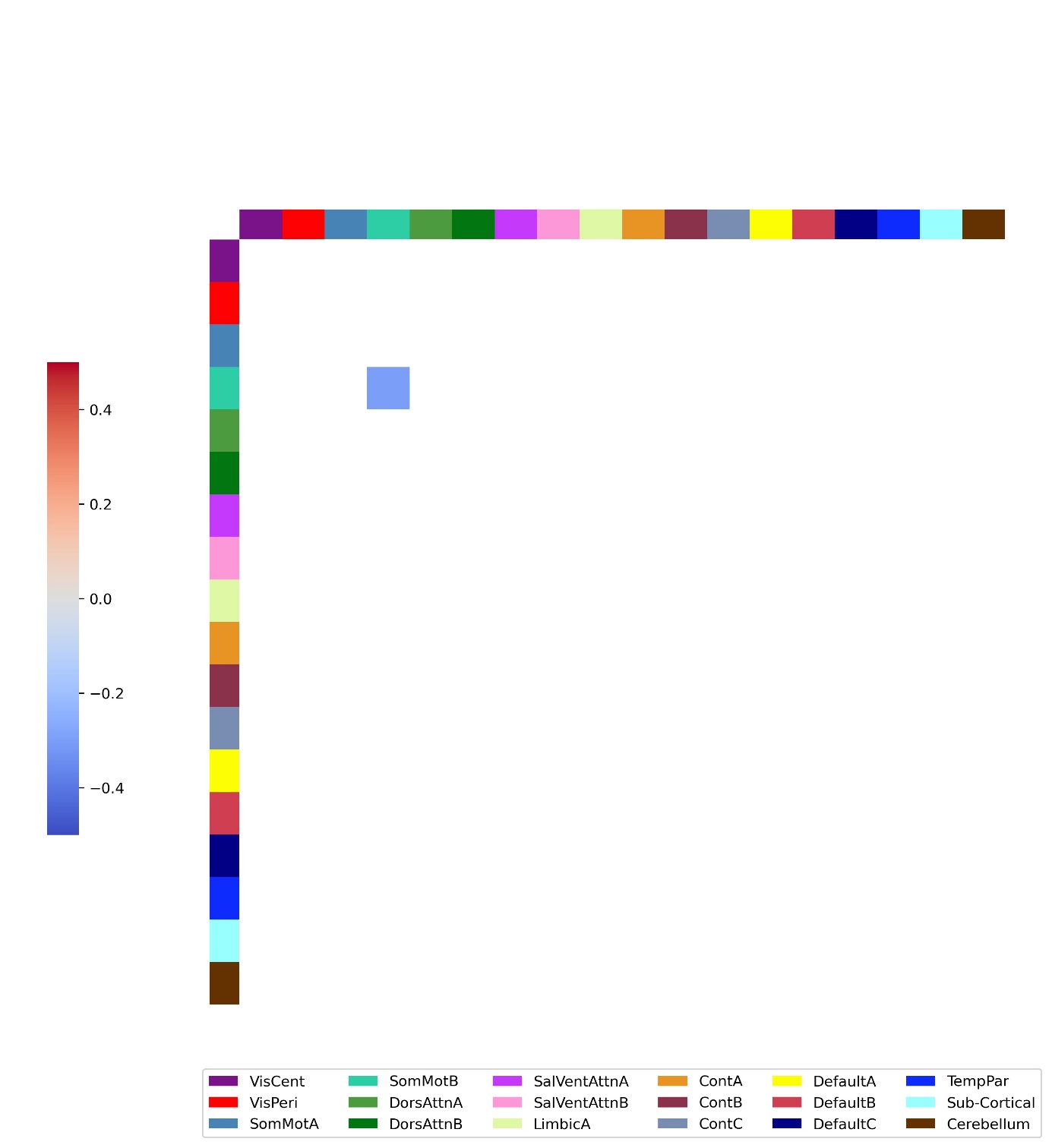


**Supplementary Figure 14.** Effect sizes (Cohen’s *d*) for group differences in between and within-network functional connectivity between high-severity OCD patients and controls.


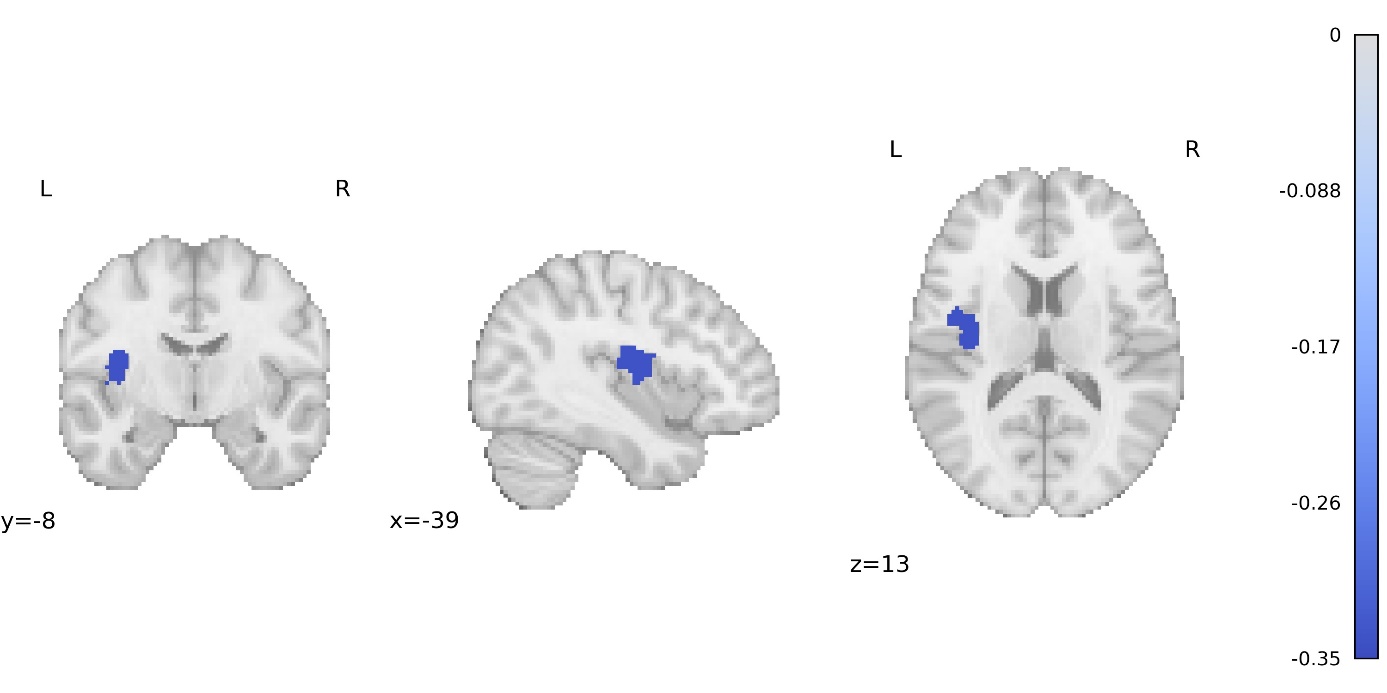


**Supplementary Figure 15.** Effect sizes for group differences in regional fALFF between high-severity OCD patients and controls.


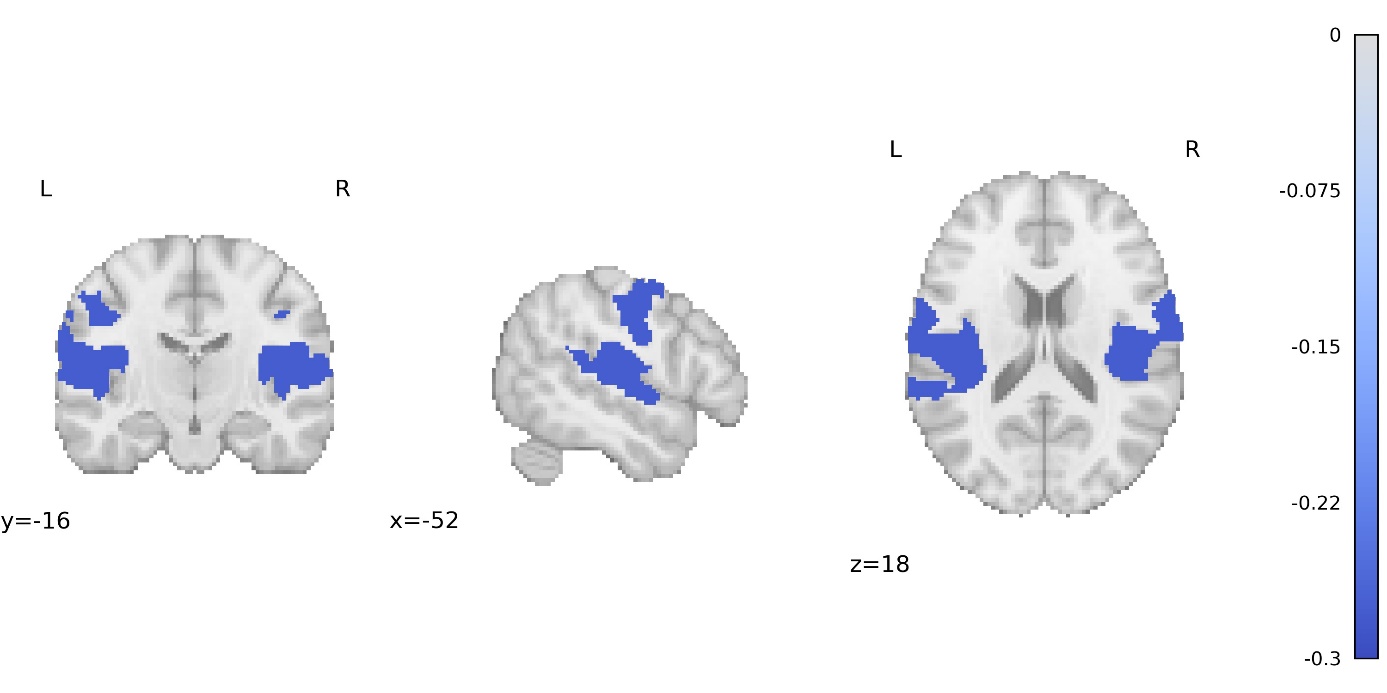


**Supplementary Figure 16.** Effect size for group difference in network-wise fALFF between high-severity OCD patients and controls.


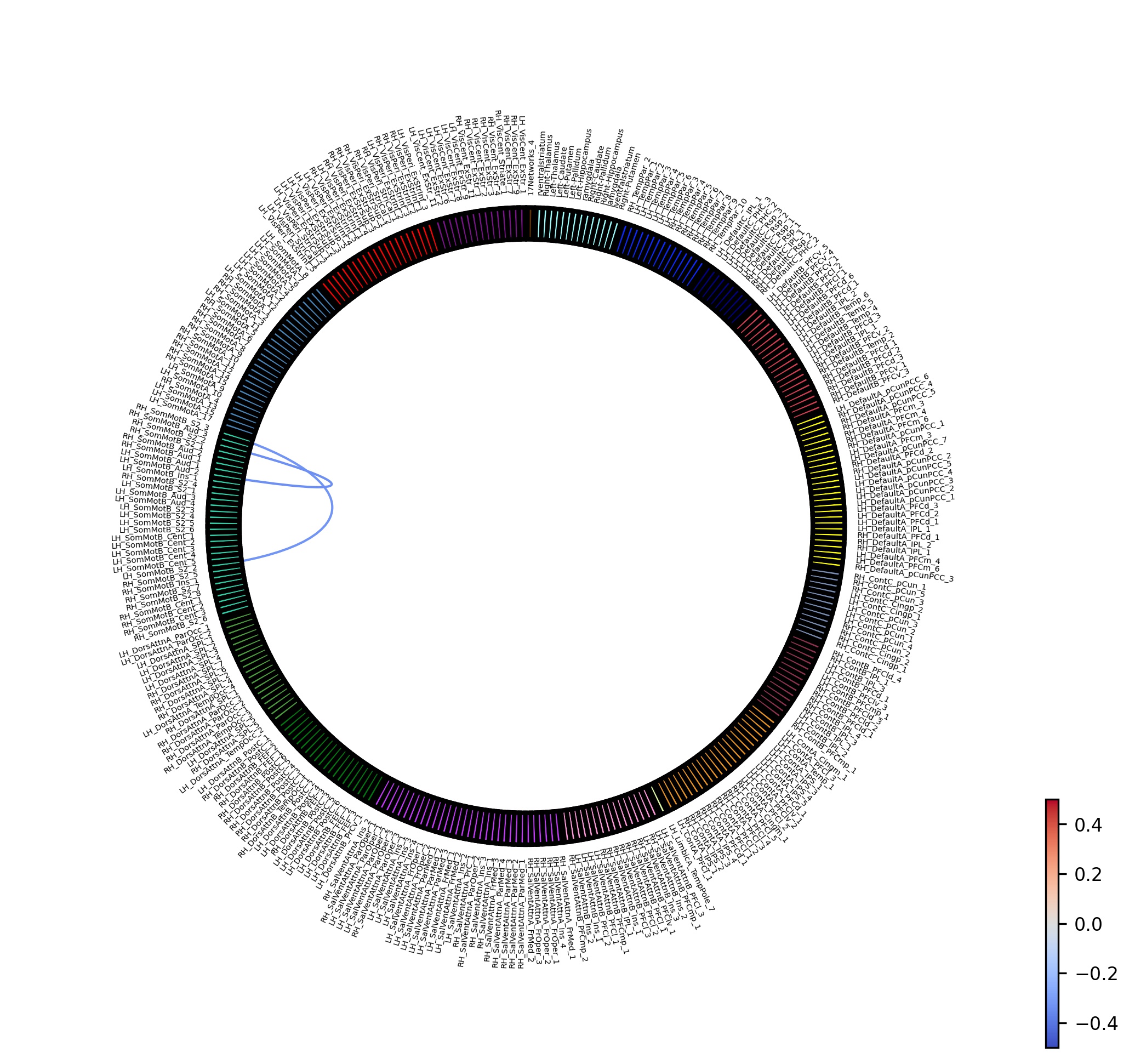


**Supplementary Figure 17.** Effect sizes (Cohen’s *d*) for group differences in ROI-to-ROI functional connectivity between adult, late age of onset OCD patients and controls.


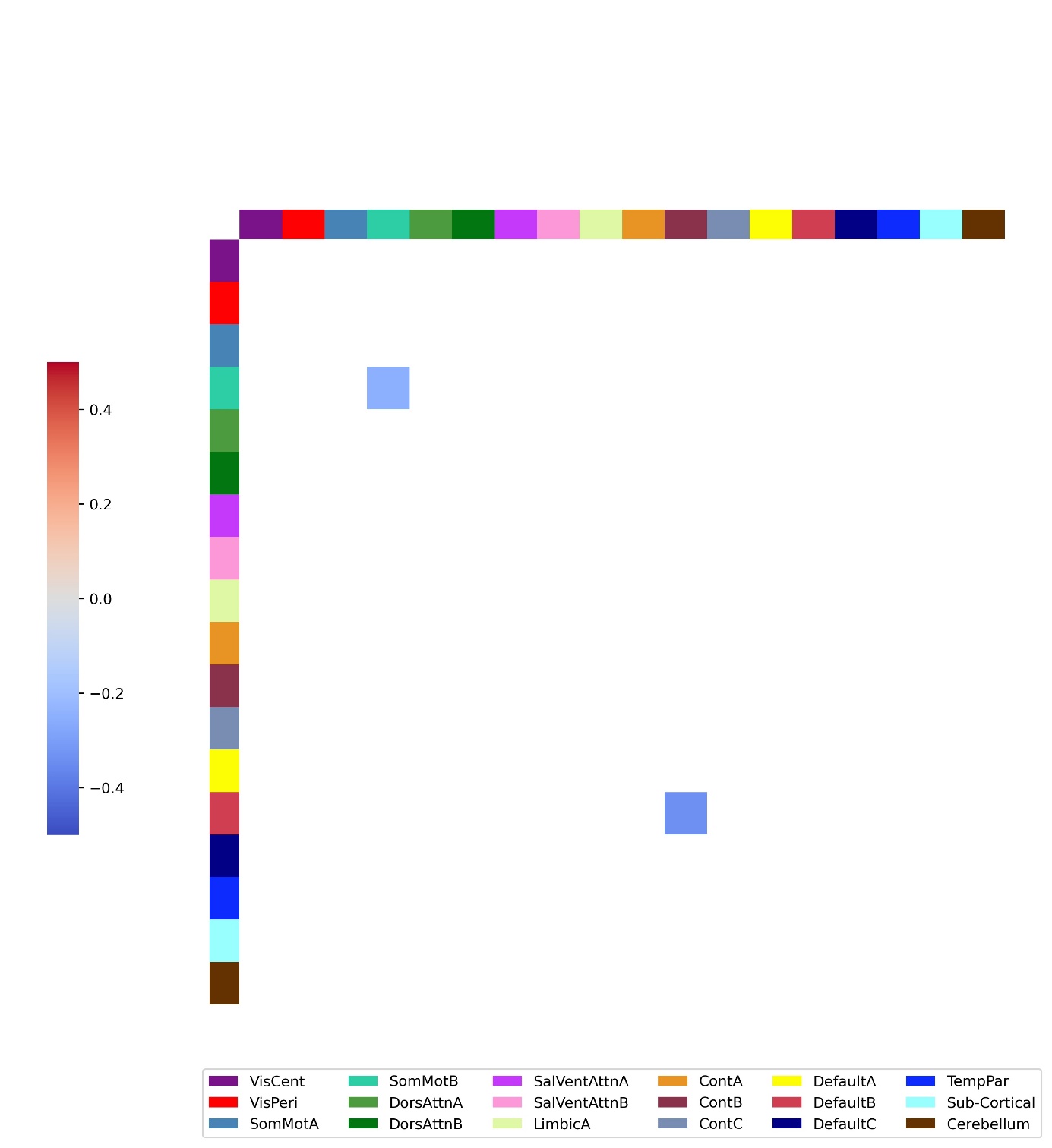


**Supplementary Figure 18.** Effect sizes (Cohen’s *d*) for group differences in between and within-network functional connectivity between adult, late age of onset OCD patients and controls.


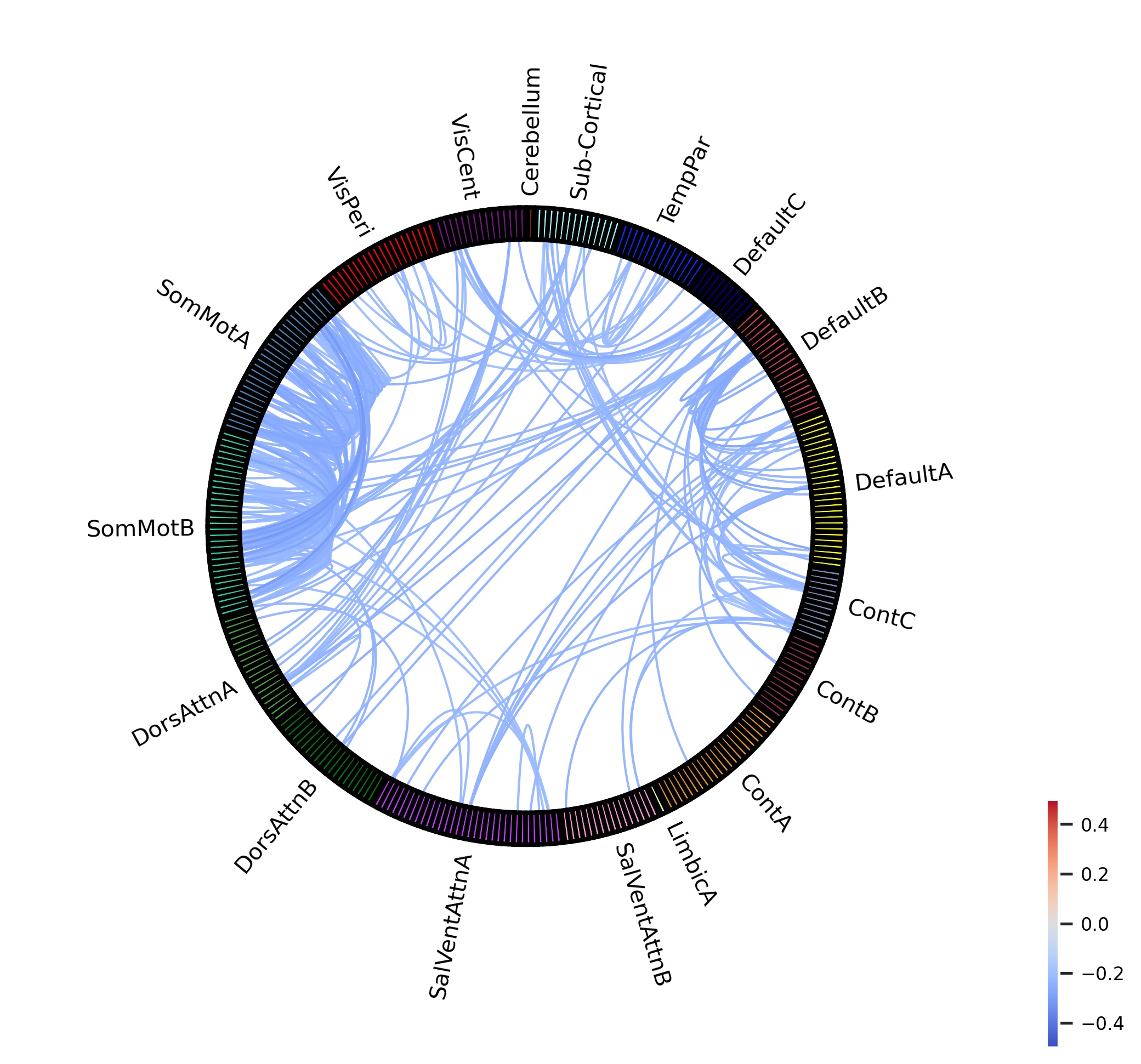


**Supplementary Figure 19.** Effect sizes (Cohen’s *d*) for group differences in ROI-to-ROI functional connectivity between OCD patients and controls using a subsample of data matched for age, sex and average framewise displacement. TempPar = Temporal Parietal, Cont = Frontoparietal Control, SalVentAttn = Salience/Ventral Attention, DorsAttn = Dorsal Attention, SomMot = Sensorimotor, VisCent = Visual Central (Visual A), VisPeri = Visual Peripheral (Visual B).


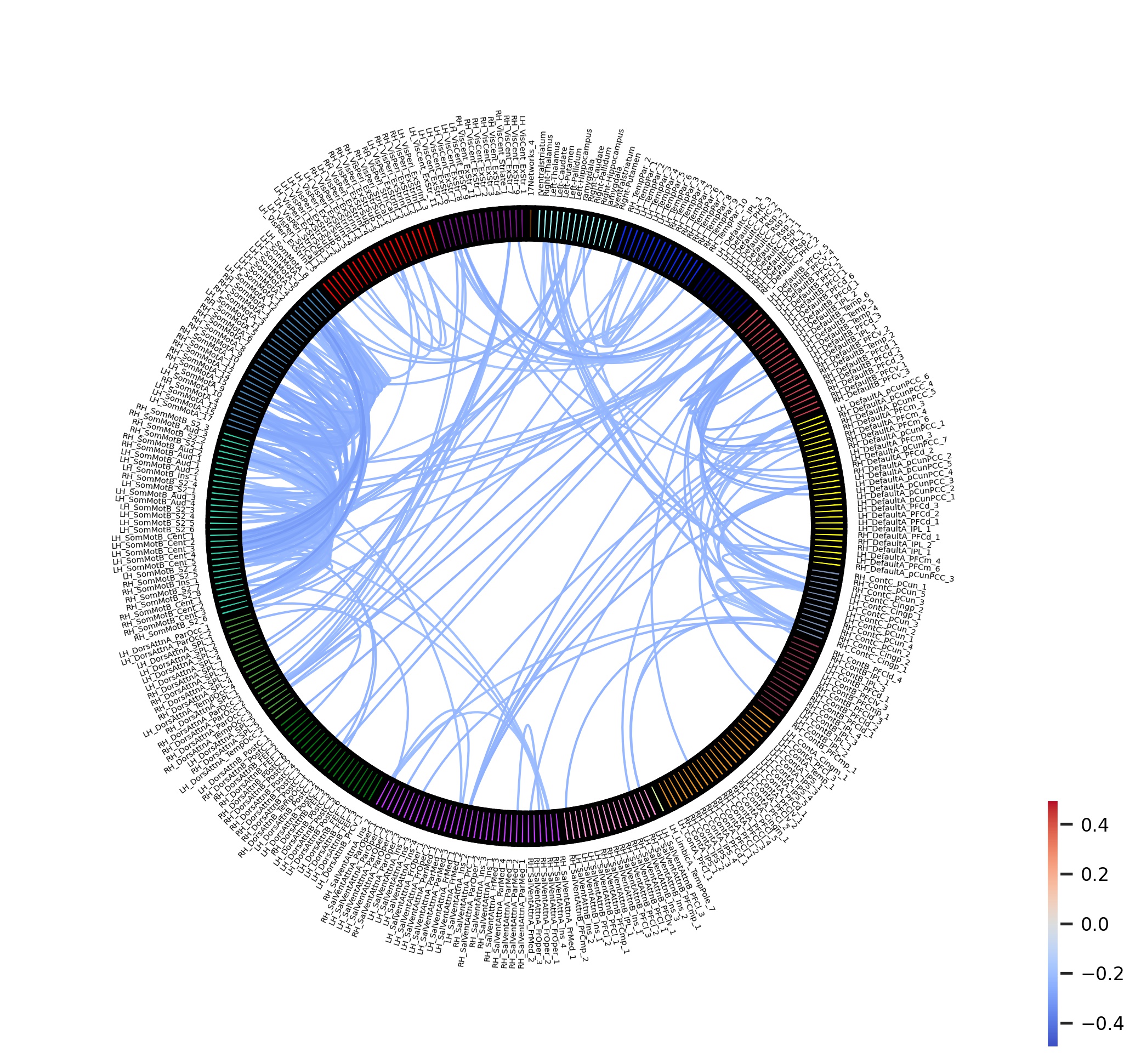


**Supplementary Figure 20.** Effect sizes (Cohen’s *d*) for group differences in ROI-to-ROI functional connectivity between OCD patients and controls using a subsample of data matched for age, sex and average framewise displacement.


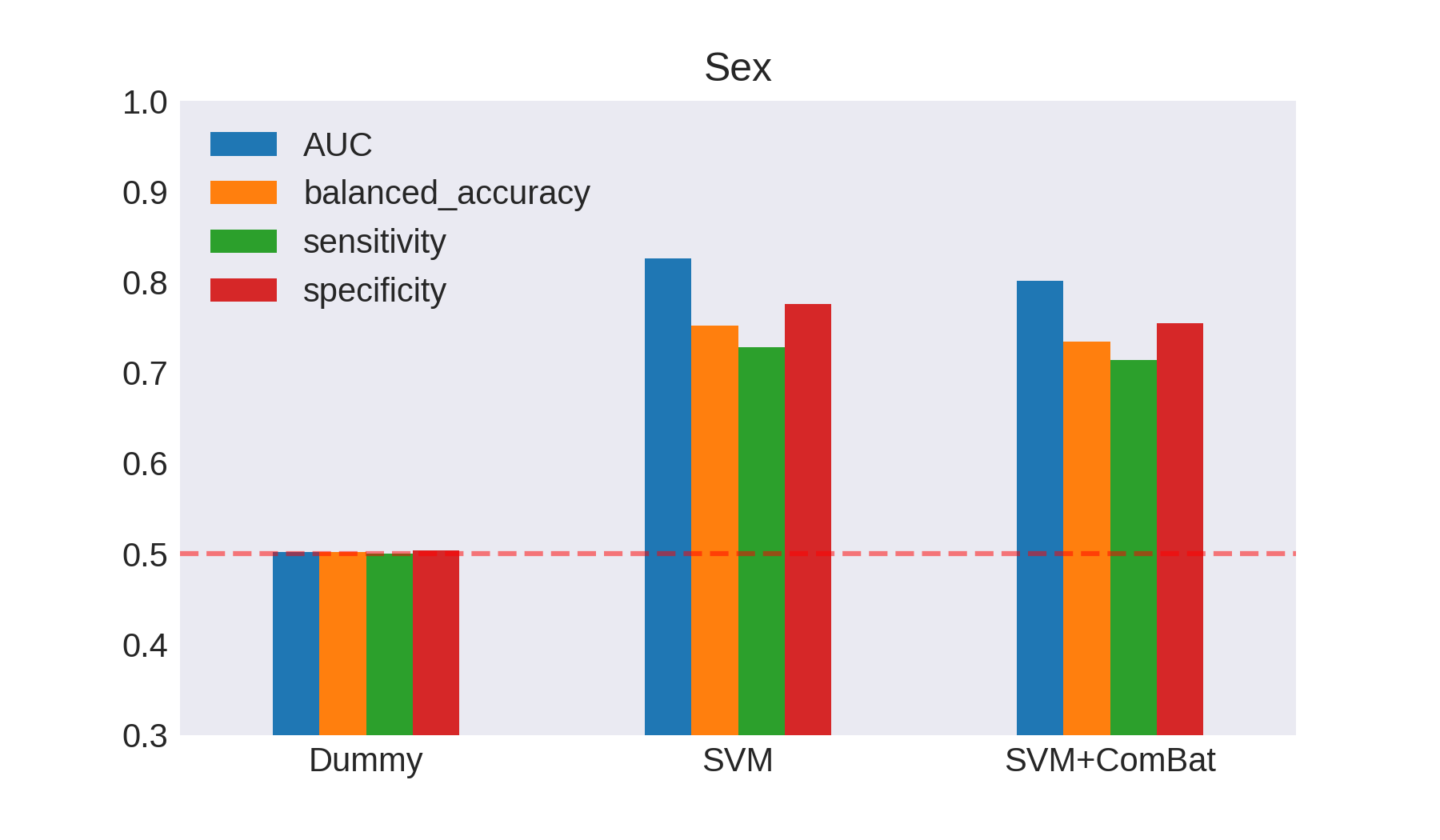


**Supplementary Figure 21.** Performances obtained for sex classification using regional functional connectivity for dummy classifier and support vector machines combined with- and without ComBat harmonization. Dashed line depicts chance-level performance.


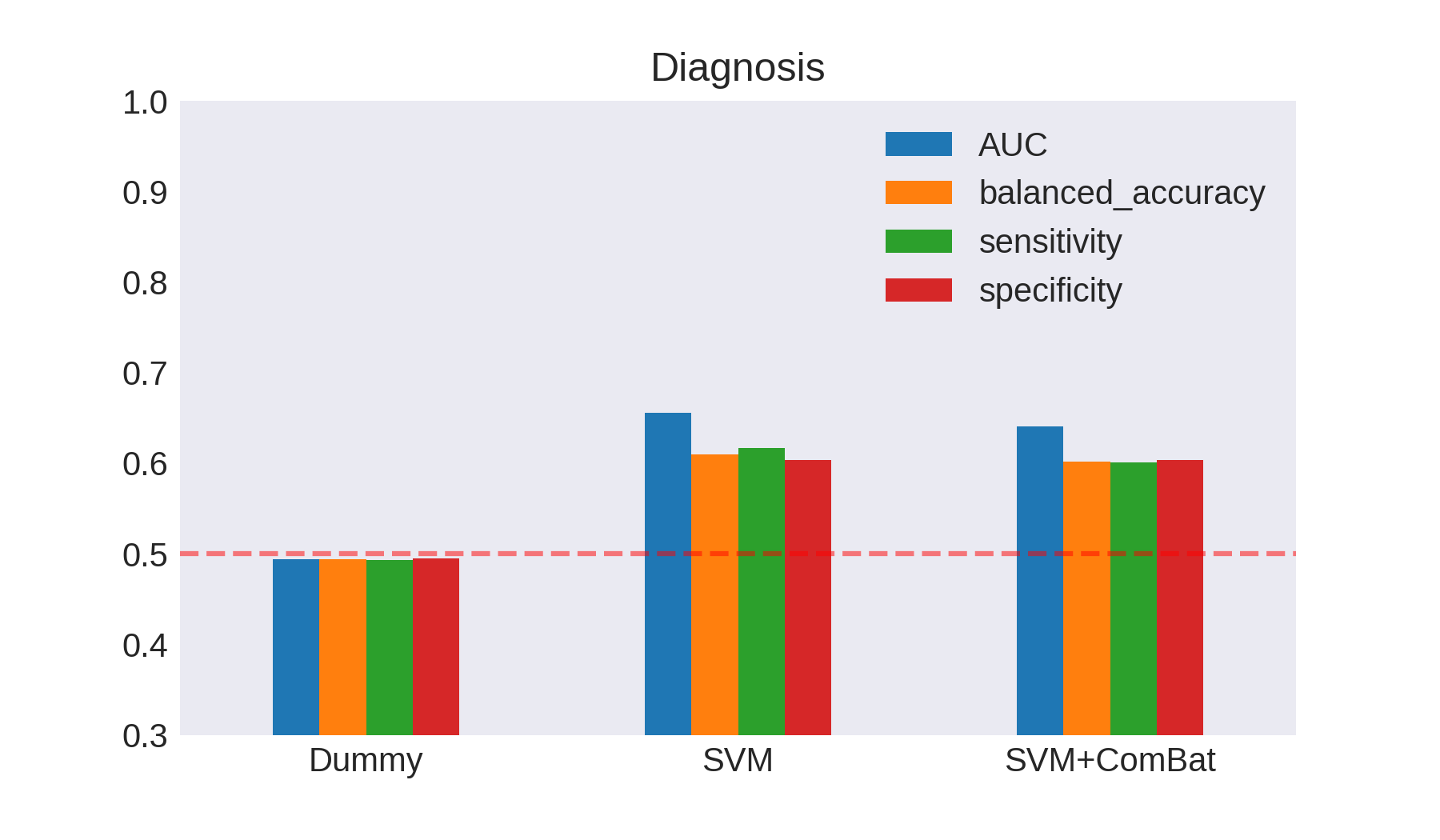


**Supplementary Figure 22.** Performances obtained for diagnosis classification using regional functional connectivity for dummy classifier and support vector machines combined with- and without ComBat harmonization. Dashed line depicts chance-level performance.


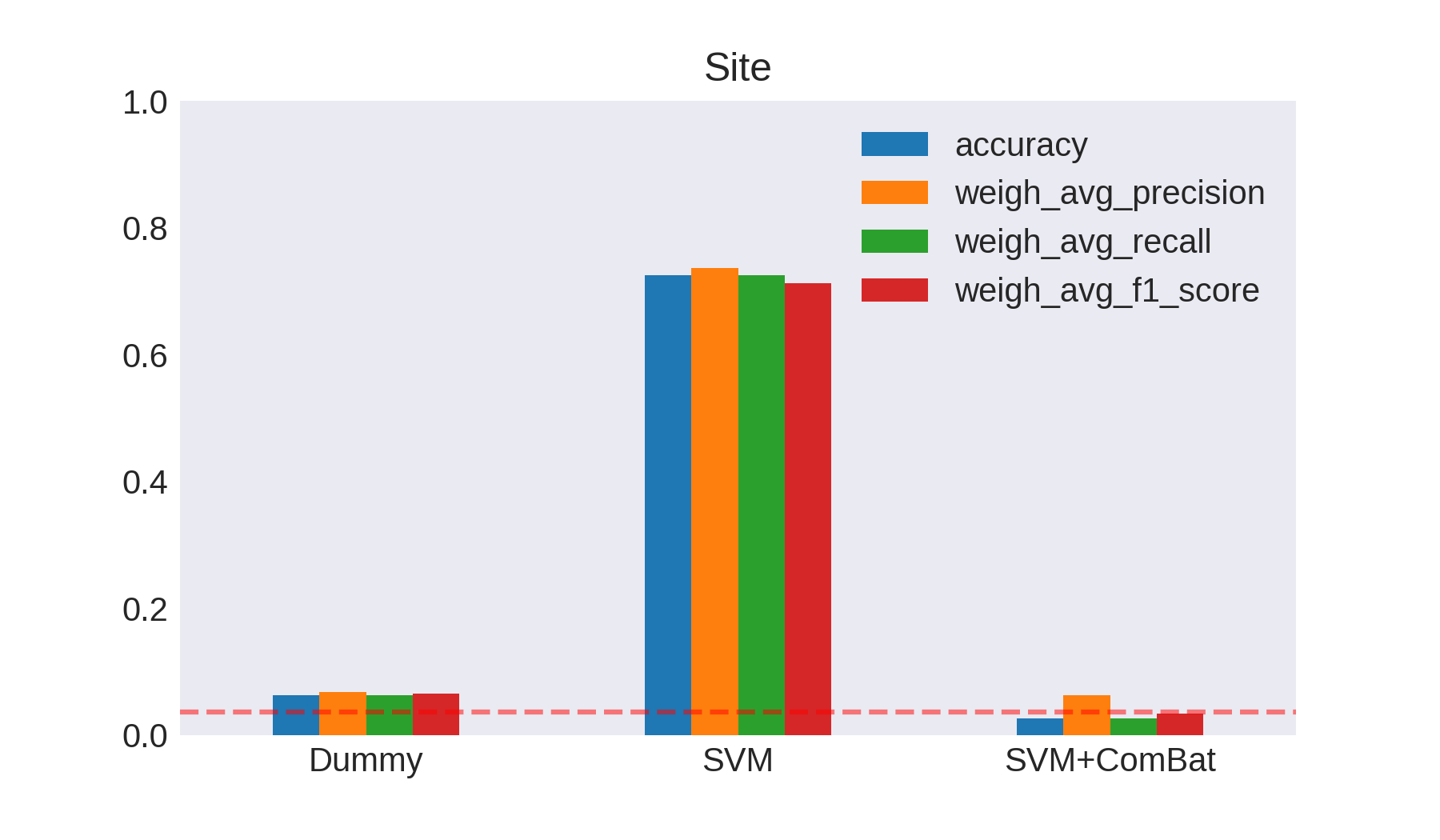


**Supplementary Figure 23.** Performances obtained for multiclass sample ID classification using regional functional connectivity for dummy classifier and support vector machines combined with- and without ComBat harmonization. Dashed line depicts chance-level performance.

**References**

1. First MB. Structured clinical interview for DSM-IV axis I disorders. Biometrics Research Department. 1997.

2. Sheehan DV, Lecrubier Y, Sheehan KH, Amorim P, Janavs J, Weiller E, et al. The Mini-International Neuropsychiatric Interview (MINI): the development and validation of a structured diagnostic psychiatric interview for DSM-IV and ICD-10. Journal of clinical psychiatry. 1998;59(20):22-33.

3. Silverman WK. Anxiety disorders interview schedule for DSM-IV.: parent interview schedule: Oxford University Press; 1996.

4. Kaufman J, Birmaher B, Brent D, Rao U, Flynn C, Moreci P, et al. Schedule for affective disorders and schizophrenia for school-age children-present and lifetime version (K-SADS-PL): initial reliability and validity data. Journal of the American Academy of Child & Adolescent Psychiatry. 1997;36(7):980-8.

5. Sheehan DV, Sheehan KH, Shytle RD, Janavs J, Bannon Y, Rogers JE, et al. Reliability and validity of the mini international neuropsychiatric interview for children and adolescents (MINI-KID). The Journal of clinical psychiatry. 2010;71(3):17393.

6. Waller L, Erk S, Pozzi E, Toenders YJ, Haswell CC, Büttner M, et al. ENIGMA HALFpipe: Interactive, reproducible, and efficient analysis for resting-state and task-based fMRI data. bioRxiv. 2021.

7. Esteban O, Markiewicz CJ, Blair RW, Moodie CA, Isik AI, Erramuzpe A, et al. fMRIPrep: a robust preprocessing pipeline for functional MRI. Nat Methods. 2019;16(1):111-6.

8. Pruim RHR, Mennes M, van Rooij D, Llera A, Buitelaar JK, Beckmann CF. ICA-AROMA: A robust ICA-based strategy for removing motion artifacts from fMRI data. Neuroimage. 2015;112:267-77.

9. Ciric R, Wolf DH, Power JD, Roalf DR, Baum GL, Ruparel K, et al. Benchmarking of participant-level confound regression strategies for the control of motion artifact in studies of functional connectivity. Neuroimage. 2017;154:174-87.

10. Pruim RHR, Mennes M, Buitelaar JK, Beckmann CF. Evaluation of ICA-AROMA and alternative strategies for motion artifact removal in resting state fMRI. Neuroimage. 2015;112:278-87.

11. Muschelli J, Nebel MB, Caffo BS, Barber AD, Pekar JJ, Mostofsky SH. Reduction of motion-related artifacts in resting state fMRI using aCompCor. Neuroimage. 2014;96:22-35.

12. Parkes L, Fulcher B, Yucel M, Fornito A. An evaluation of the efficacy, reliability, and sensitivity of motion correction strategies for resting-state functional MRI. Neuroimage. 2018;171:415-36.

13. Schaefer A, Kong R, Gordon EM, Laumann TO, Zuo XN, Holmes AJ, et al. Local-Global Parcellation of the Human Cerebral Cortex from Intrinsic Functional Connectivity MRI. Cereb Cortex. 2018;28(9):3095-114.

14. Desikan RS, Segonne F, Fischl B, Quinn BT, Dickerson BC, Blacker D, et al. An automated labeling system for subdividing the human cerebral cortex on MRI scans into gyral based regions of interest. Neuroimage. 2006;31(3):968-80.

15. Buckner RL, Krienen FM, Castellanos A, Diaz JC, Yeo BT. The organization of the human cerebellum estimated by intrinsic functional connectivity. J Neurophysiol. 2011;106(5):2322-45.

16. Wu J, Eickhoff SB, Hoffstaedter F, Patil KR, Schwender H, Yeo BTT, et al. A Connectivity-Based Psychometric Prediction Framework for Brain-Behavior Relationship Studies. Cereb Cortex. 2021;31(8):3732-51.

17. El Gazzar A, Cerliani L, van Wingen G, Thomas RM, editors. Simple 1-D convolutional networks for resting-state fMRI based classification in autism. 2019 International Joint Conference on Neural Networks (IJCNN); 2019: IEEE.

18. Yarkoni T, Poldrack RA, Nichols TE, Van Essen DC, Wager TD. Large-scale automated synthesis of human functional neuroimaging data. Nat Methods. 2011;8(8):665-70.

19. Zang Y, Jiang T, Lu Y, He Y, Tian L. Regional homogeneity approach to fMRI data analysis. Neuroimage. 2004;22(1):394-400.

20. Zou QH, Zhu CZ, Yang Y, Zuo XN, Long XY, Cao QJ, et al. An improved approach to detection of amplitude of low-frequency fluctuation (ALFF) for resting-state fMRI: fractional ALFF. J Neurosci Methods. 2008;172(1):137-41.

21. Jolly E. Pymer4: Connecting R and Python for Linear Mixed Modeling. Journal of Open Source Software. 2018;3(31).

22. Kuznetsova A, Brockhoff PB, Christensen RHB. lmerTest Package: Tests in Linear Mixed Effects Models. Journal of Statistical Software. 2017;82(13).

23. Bates D, Mächler M, Bolker B, Walker S. Fitting linear mixed-effects models using lme4. arXiv preprint arXiv:14065823. 2014.

24. Nakagawa S, Cuthill IC. Effect size, confidence interval and statistical significance: a practical guide for biologists. Biol Rev Camb Philos Soc. 2007;82(4):591-605.

25. Storch EA, De Nadai AS, Conceicao do Rosario M, Shavitt RG, Torres AR, Ferrao YA, et al. Defining clinical severity in adults with obsessive-compulsive disorder. Compr Psychiatry. 2015;63:30-5.

26. Boedhoe PSW, Schmaal L, Abe Y, Alonso P, Ameis SH, Anticevic A, et al. Cortical Abnormalities Associated With Pediatric and Adult Obsessive-Compulsive Disorder: Findings From the ENIGMA Obsessive-Compulsive Disorder Working Group. Am J Psychiatry. 2018;175(5):453-62.

27. Boedhoe PS, Schmaal L, Abe Y, Ameis SH, Arnold PD, Batistuzzo MC, et al. Distinct Subcortical Volume Alterations in Pediatric and Adult OCD: A Worldwide Meta- and Mega-Analysis. Am J Psychiatry. 2017;174(1):60-9.

28. Chang C-c, Lin C-j. LIBSVM : A Library for Support Vector Machines. ACM Transactions on Intelligent Systems and Technology (TIST). 2013;2:1-39.

29. Ojala M, Garriga GC. Permutation Tests for Studying Classi er Performance. Journal ofMachine Learning Research. 2010;11:1833-63.

30. Kottas M, Kuss O, Zapf A. A modified Wald interval for the area under the ROC curve (AUC) in diagnostic case-control studies. BMC Med Res Methodol. 2014;14:26.

31. Fortin JP, Cullen N, Sheline YI, Taylor WD, Aselcioglu I, Cook PA, et al. Harmonization of cortical thickness measurements across scanners and sites. Neuroimage. 2018;167:104-20.

32. Kline A, Luo Y, editors. PsmPy: a package for retrospective cohort matching in python. 2022 44th Annual International Conference of the IEEE Engineering in Medicine & Biology Society (EMBC); 2022: IEEE.
